# Supplementary material for: Developing an intervention to increase REferral and uptake TO pulmonary REhabilitation in primary care in patients with chronic obstructive pulmonary disease (the REsTORE study): mixed methods study protocol
Source: BMJ Open. 2019 Jan 21;9(1):e024806. doi: 10.1136/bmjopen-2018-024806 (PMC6347857; doi:10.1136/bmjopen-2018-024806)
Supplement: Supplementary data [file bmjopen-2018-024806supp009.pdf]

INCREASING REFERRAL AND UPTAKE TO PULMONARY REHABILITATION: A NEW WAY TO HELP PRIMARY CARE TEAMS

DRAFT Patient Questionnaire

Thank you for taking part in our research about patients' with breathing problems and their experiences of care in General Practices in Cambridgeshire. In particular we are interested in how you found your consultation e.g. how long it took, if you understood things and if it was helpful. You can also give your comments about things you like, dislike or would like to see improved. Your answers remain anonymous and will not be directly reported to the staff at the practice.

Please answer all the questions and return the questionnaire to the University of Cambridge using the stamped addressed envelope.

Thank you for taking part in the survey

Dr Frances Early, Research & Evaluation Lead, Centre for Self-Management Support ,

contact: Tel: 01223 245151, Ext: 4858 e-mail: frances.early@medschl.cam.ac.uk

Section 1 Some questions about you

1.1 Are you Female / Male / Prefer not to answer (please delete as applicable)

1.2 Are you aged (please tick)

under 40 years ……..

40 – 50 years ……..

50 – 60 years ………

60 to 70 years ……….

70 to 80 years ………..

Over 80 years ………..

or Prefer not to answer……….. (tick if applicable)

1.3 About how many years have you had problems with your breathing? …….. years

1.4 In the past have you ever attended Pulmonary Rehabilitation classes? (please delete as applicable)

Yes / No / Don't Know

Section 2. Some questions about your experience of the consultation

2.1 Overall, how easy or difficult did you find your consultation? (please tick)

Very easy

Quite easy

Neither easy or difficult

Quite difficult

Very difficult

2.2 Did you find the length of the consultation….(please tick)

Too long……………… About right………… Too short……….

2.3 During the consultation did any of the following happen? (tick those that apply to you)

Had some breathing tests ……….

Had a discussion with the nurse ……….

Watched a short video film ……….

Received a leaflet or written information ………

Received forms to fill out ………

Other (please specify below) ……….

……………………………………………………….

2.4 How helpful did you find the consultation? (please tick)

Very Helpful

Quite helpful

Neither helpful or unhelpful

Quite unhelpful

Very unhelpful

2.5 How easy or difficult was it to understand the consultation? (please tick)

Very easy

Quite easy

Neither easy or difficult

Quite difficult

Very difficult

2.6 Do you have any comments about the consultation or our survey? e.g. anything you liked, anything you disliked, any suggestions of what we could do better?

…………………………………………………………………………..

………………………………………………………………………….

Thank You Again for Taking Part in the Survey

Frances Early, PhD, CPsychol , Research & Evaluation Lead, Centre for Self-Management Support ,

contact: Tel: 01223 245151, Ext: 4858 e-mail: frances.early@medschl.cam.ac.uk
