# Supplementary material for: Developing an intervention to increase REferral and uptake TO pulmonary REhabilitation in primary care in patients with chronic obstructive pulmonary disease (the REsTORE study): mixed methods study protocol
Source: BMJ Open. 2019 Jan 21;9(1):e024806. doi: 10.1136/bmjopen-2018-024806 (PMC6347857; doi:10.1136/bmjopen-2018-024806)
Supplement: Supplementary data [file bmjopen-2018-024806supp001.pdf]

Cambridge University Hospitals NHS
NHS Foundation Trust

The REsTORE project: Increasing REferral and uptake TO Pulmonary REhabilitation (general practice survey)

Page 1: Introduction

Thank you for taking part in this survey. The aim is to understand blocks and facilitators to referral and uptake of pulmonary rehabilitation (PR) in primary care and how referral and uptake can be improved.

The survey is part of a research study funded by the National Institute for Health Research (Research for Patient Benefit Programme) that aims to increase the number of people taking up PR. The study also includes interviews and focus groups with patients and clinicians to help us understand what happens during the referral process. We will use what we have learned to develop a toolkit of resources to support referral in primary care, working collaboratively with patients and clinicians.

We request that the survey is completed by a member of clinical staff in your practice who is involved with PR referrals.

The survey should take about 10 to 20 minutes. There are four parts:

- Your role
- Your experience of the PR referral process
- Improving referral and uptake of PR
- Getting in touch

There are no right or wrong answers. All responses and comments are valuable to us. At the end you will be able to leave any additional comments you wish to make.

Your response is valuable to us and so your practice will be paid £10 for the clinical staff

time needed to complete it. In addition, we invite you to enter a prize draw to win £200 in online shopping vouchers as an incentive to take part. The project is registered on the NIHR CRN Portfolio and completion will count as an accrual for your practice.

On the next two pages you will find participant information about the survey and a consent form for your participation. Please complete the consent form before continuing.

Please complete the survey as soon as possible.

Thank you for your participation.

I confirm that I am a member of clinical staff in my practice who is involved with referral to pulmonary rehabilitation ✱ *Required*

- ○ Yes

Page 2: Participant Information

You are invited to take part in an online survey as part of a project to help increase referral and uptake to pulmonary rehabilitation for people with Chronic Obstructive Pulmonary Disease (COPD).

1. What is the purpose of the study?

Our study aims to increase the number of people with COPD who attend pulmonary rehabilitation by developing an online toolkit to help GP practices refer patients successfully. Pulmonary rehabilitation offers supervised exercise and education and helps people to manage their COPD symptoms, improve their quality of life and avoid admission to hospital. It is recommended by NICE. However, the National COPD Audit Programme reported that in 2013/14 an estimated 446,000 COPD patients in England and Wales were eligible for pulmonary rehabilitation but that only 68,000 referrals were made. Of those, only 69% took up the referral offer.

This survey of healthcare professionals (HCPs) in primary care who care for people with COPD is the first step in developing the toolkit. We are also surveying pulmonary rehabilitation providers. Following the survey we will hold interviews and focus groups with patients and HCPs to gather ideas about how to make referral easier and how to help patients make informed decisions about whether to attend pulmonary rehabilitation. We will combine these ideas with evidence from published research and then work with patients and HCPs to assemble the information in an online toolkit. The toolkit could contain, for example, patient-friendly information, electronic reminders on patient records or simplified referral processes. It will be designed to integrate with primary care working practices and to be used, for example, during an annual COPD review. We will test the toolkit in primary care to ensure it is practical for the NHS and has the potential to make a difference.

2. Why have I been chosen to take part in the survey?

We are inviting members of clinical staff in general practices in the East of England who are involved in referral to pulmonary rehabilitation.

3. What will I need to do?

The survey will take about 10 to 20 minutes to complete and is designed to be quick and easy. It will ask about your experiences of referring patients to pulmonary rehabilitation and for your ideas about how to improve referral and uptake. At the end of the survey, we will invite you to provide your contact details if you would like to take part in a subsequent interview or focus group on this topic.

4. How will the findings be used?

The survey results will be reported in a way that preserves confidentiality and you will not be identified in any way. The results may be published on the Addenbrooke's Hospital intranet, in peer reviewed medical journals, and used for medical presentations, conferences or presentations to patient groups. If you would like to be kept informed of the results please contact the Research Manager, Dr Frances Early, using the details below.

5. What will happen to the information I provide?

All information that is collected is strictly confidential. Only members of the research team will have access to the information. Where data are referred to in published material participants will not be identifiable. Data will be held in secure storage and destroyed after five years. Any information that you give will be used for research purposes only and you may ask to see your personal information at any time.

6. Can I withdraw from the project?

You may withdraw from the study at any time without giving a reason. If you withdraw we will only retain and use any personal information you have provided up to that point if you give us permission to do so.

7. Who has organised the research?

The Lead Investigator for the study is Dr Jonathan Fuld at Cambridge University Hospitals NHS Foundation Trust. Other members of the research team are:

Dr Frances Early (Cambridge University Hospitals NHS Foundation Trust)

Professor Christi Deaton, Dr Ian Wellwood, Dr John Benson, Dr Lois Kim (Cambridge Institute of Public Health)

Ruth Barlow, Lianne Jongepier (East of England Pulmonary Rehabilitation Network)

Professor Patricia Wilson (University of Kent)

Professor Sally Singh (University Hospitals of Leicester NHS Foundation Trust)

The British Lung Foundation

8. How has the study been funded?

The research is funded by the National Institute for Health Research as part of their Research for Patient Benefit programme (award no: PB-PG-1215-20034).

9. Further information and contact details

If you would like more information please contact Dr Frances Early, Box 146, Cambridge University Hospitals NHS Foundation Trust, Hills Road, Cambridge, CB2 0QQ. Tel 01223 274858. Email: frances.early@addenbrookes.nhs.uk

Ref (WP2 A2 PIS Survey Primary Care v1.1 260617) IRAS ID 209597

Page 3: Consent Form

Please tick each box below to indicate that you understand and agree with the statements

- ☐ I confirm that I have read and understood the information sheet on page 2 of this survey. I have had the opportunity to consider the information, ask questions and have had questions answered satisfactorily
- ☐ I understand my participation is voluntary and I may withdraw at any time without giving any reason
- ☐ I agree that the information I provide can be used solely for the purpose of this reasearch
- ☐ I agree to the use of anonymised data in publications
- ☐ I understand that all personal information will remain confidential
- ☐ I agree to take part in the above study

I confirm my agreement to the above statements ✱ *Required*

Page 4: Your role

Which best describes your professional job category?

- ◯ Practice nurse
- ◯ Nurse practitioner
- ◯ Advanced nurse practitioner
- ◯ Nurse consultant
- ◯ Specialist respiratory nurse
- ◯ Postgraduate diploma in respiratory care
- ◯ GP
- ◯ Other

If other, please give details

What responsibilities do you have for the care of people with COPD? Tick as many as apply.

- ☐ Annual reviews
- ☐ Urgent assessments
- ☐ Ongoing management
- ☐ Spirometry
- ☐ Oxygen therapy
- ☐ Admission prevention
- ☐ Early discharge facilitation
- ☐ Prescribing
- ☐ Medication checks
- ☐ Opportunistic non-urgent care
- ☐ Other

If other, please give details

How many years' experience do you have of caring for people with respiratory problems?

Please enter a whole number (integer).

How many years have you worked in primary care?

Please enter a whole number (integer).

Please tell us the name and postcode of your general practice so that we can manage the payment to your practice and identify the list size and COPD register details from public sources

Page 5: The pulmonary rehabilitation (PR) referral process (i)

To which PR services does your general practice refer patients? Tick as many as apply.

- ☐ Norfolk Community (Norfolk Community Health and Care NHS Trust)
- ☐ West Norfolk (BOC Clinical Services)
- ☐ Norfolk & Norwich (Norfolk & Norwich University Hospital NHS Trust)
- ☐ Suffolk (Suffolk Community Healthcare)
- ☐ Great Yarmouth & Waveney: BEET (James Paget University Hospitals NHS FT)
- ☐ West Essex (Essex Partnership University NHS FT)
- ☐ South West Essex (NE London NHS FT)
- ☐ Mid Essex (Provide)
- ☐ North East Essex (Anglian Community Enterprise)
- ☐ Southend (Southend University Hospital NHS FT)
- ☐ Colchester Hospital (Colchester Hospital University NHS FT)
- ☐ Huntingdon & Brookfield (Cambridgeshire & Peterborough NHS FT)
- ☐ Cambridgeshire & Peterborough (Provide)
- ☐ Peterborough and Stamford (Peterborough and Stamford Hospitals NHS FT)
- ☐ Papworth Hospital (Papworth Hospital NHS FT)
- ☐ Luton & Dunstable (Luton & Dunstable University Hospital NHS FT)
- ☐ Luton Community (Cambridgeshire Community NHS FT)
- ☐ Bedford Hospital (Bedford Hospital NHS Trust)
- ☐ East & North Herts (Hertfordshire Community NHS Trust)
- ☐ West Herts (Central London Community Healthcare NHS Trust)
- ☐ Other

If other, please give details

I feel sure that I understand the eligibility criteria for PR contained in the BTS Guideline on Pulmonary Rehabilitation in Adults

Not at all	☐	☐	☐	☐	☐	☐	☐	☐	☐	☐	☐	Completely

I feel adequately prepared to refer patients to PR

Not at all	☐	☐	☐	☐	☐	☐	☐	☐	☐	☐	☐	Completely

Referring patients to PR is currently a normal part of my work

Not at all	☐	☐	☐	☐	☐	☐	☐	☐	☐	☐	☐	Completely

I find the process of referring patients to PR is easy

Not at all easy	☐	☐	☐	☐	☐	☐	☐	☐	☐	☐	☐	Very easy

What resources (e.g. tools, systems or processes) are available in your general practice to support clinicians in making PR referrals? Tick all that apply.

- ☐ Prompts in clinical system
- ☐ Information about PR for clinicians
- ☐ Clear referral criteria

- [ ] Electronic referral
- [ ] Skills training for communicating effectively with patients
- [ ] Other

If other, please give details

What resources (e.g. tools, systems or processes) are available in your general practice to support patients in deciding whether to take up a referral? Tick all that apply.

- [ ] Information about PR for patients
- [ ] Shared decision-making tools
- [ ] Other

If other, please give details

Page 6: The pulmonary rehabilitation (PR) referral process (ii)

What factors influence your decision to refer a patient to PR?

	No influence	Some influence	Strong influence
Increasing shortness of breath	☐	☐	☐
MRC Dyspnoea Scale ≥3	☐	☐	☐
Mobility is affected by breathlessness	☐	☐	☐
Decreasing activity levels	☐	☐	☐
Frequent exacerbations	☐	☐	☐
Recent hospital discharge after exacerbation	☐	☐	☐
Patient is deconditioned	☐	☐	☐
Low exercise tolerance	☐	☐	☐
Patient anxiety	☐	☐	☐
Poor self-management	☐	☐	☐
Easy to use referral process	☐	☐	☐
Other	☐	☐	☐

If other, please give details

What factors might influence your decision *not* to refer an eligible patient to PR?

	No influence	Some influence	A strong influence
I don't have enough information about PR	☐	☐	☐

I'm uncertain that PR is worthwhile	☐	☐	☐
Patient has doubts that PR is worthwhile	☐	☐	☐
Patient refuses referral	☐	☐	☐
Patient co-morbidities	☐	☐	☐
Poor mobility	☐	☐	☐
Patient does not fit acceptance criteria for a programme	☐	☐	☐
Patient has attended PR previously	☐	☐	☐
Transportation problems	☐	☐	☐
Distance of class from patient's home	☐	☐	☐
Lack of time to make referral	☐	☐	☐
Timing of classes not convenient for patient	☐	☐	☐
I'm unaware of how to refer	☐	☐	☐
Difficult referral process	☐	☐	☐
Other	☐	☐	☐

If other, please give details

Page 7: The pulmonary rehabilitation (PR) referral process (iii)

For each statement please select the answer that best suits your experience

	Strongly disagree	Disagree	Not sure	Agree	Strongly agree
Staff in my general practice involved in the care of people with respiratory problems have a shared understanding of the purpose of PR	☐	☐	☐	☐	☐
I can see the potential value of referring patients to PR	☐	☐	☐	☐	☐
In my general practice there are key people who drive PR referral forwards and get others involved	☐	☐	☐	☐	☐
I believe that referring patients to PR is a legitimate part of my role	☐	☐	☐	☐	☐
I'm open to working with colleagues in new ways to enable PR referral to happen	☐	☐	☐	☐	☐

I can easily integrate PR referral into my work	☐	☐	☐	☐	☐
I have confidence in the ability of other colleagues who care for people with respiratory problems to refer patients to PR	☐	☐	☐	☐	☐
In my general practice PR referral is assigned to those with appropriate skills	☐	☐	☐	☐	☐
Sufficient training is provided in my practice so that staff know who to refer to PR	☐	☐	☐	☐	☐
Sufficient training is provided in my practice so that staff know how to refer to PR	☐	☐	☐	☐	☐
Sufficient resources are available to support PR referral in my practice	☐	☐	☐	☐	☐
I am aware of reports in my practice about the outcomes for patients following a PR referral	☐	☐	☐	☐	☐

The staff in my practice agree that referring patients to PR is worthwhile	☐	☐	☐	☐	☐
I am able to overcome any difficulties I have with regard to referring patients to PR	☐	☐	☐	☐	☐

Page 8: The pulmonary rehabilitation (PR) referral process (iv)

In your experience what are the main reasons that patients take up a referral to PR?

In your experience what are the main reasons why patients decline an offer of PR and do not wish to be referred?

In your experience what are the main reasons that patients referred to PR do not attend their assessment?

Page 9: Improving referral and uptake of pulmonary rehabilitation (PR)

What would make it easier for you to refer patients to PR?

What could be done to encourage more patients to take up a referral to PR?

Do you receive adequate communication from providers of PR about their PR programmes?

- Yes
- No

Please add comments to clarify if necessary

What do you think is important for effective communication between PR providers

and primary care?

Which communication methods are effective for you when communicating with PR providers? Select all that apply.

- ☐ Email
- ☐ Letter
- ☐ Telephone
- ☐ Other

If other, please give details

This research project aims to design and build an online toolkit of resources for use in primary care to support referral to PR. What do you think such a toolkit would need to contain or do in order to be successfully adopted by clinicians in primary care?

Page 10: Other comments

Please leave any other comments you would like to add

Page 11: Getting in touch

Do you have any resources that support PR referral or uptake that you would be willing to share with the research team?

- Yes
- No

Would you like to hear about other ways to get involved in this research study, e.g. developing the toolkit or testing it in practice?

- Yes
- No

Thank you for completing this survey. All responses are confidential and results will be reported anonymously. Would you like us to share the results with you?

- Yes
- No

Would you like to be entered for the prize draw to win £200 of online shopping vouchers?

- Yes
- No

If you answered 'yes' to any of the above questions please leave your contact details here or, if you prefer to keep your responses anonymous, please email the research team directly: frances.early@addenbrookes.nhs.uk

Page 12: The survey is now complete. Thank you for your time.

Key for selection options

3 - I confirm my agreement to the above statements

Yes
No
