# Supplementary material for: Developing an intervention to increase REferral and uptake TO pulmonary REhabilitation in primary care in patients with chronic obstructive pulmonary disease (the REsTORE study): mixed methods study protocol
Source: BMJ Open. 2019 Jan 21;9(1):e024806. doi: 10.1136/bmjopen-2018-024806 (PMC6347857; doi:10.1136/bmjopen-2018-024806)
Supplement: Supplementary data [file bmjopen-2018-024806supp002.pdf]

Cambridge University Hospitals NHS
NHS Foundation Trust

The REsTORE project: Increasing REferral and uptake TO pulmonary REhabilitation (PR provider survey)

Page 1: Introduction

Thank you for taking part in this survey of pulmonary rehabilitation (PR) providers. The aims are to understand the PR referral process, the experiences of PR providers, barriers and facilitators to referral and uptake of PR from primary care and how patients can be encouraged to take up a referral.

The survey is part of a research study funded by the National Institute for Health Research (Research for Patient Benefit Programme) that aims to increase the number of people taking up PR. The study also includes a survey of general practices, and interviews and focus groups with patients and clinicians to help us understand what happens during the referral process. We will use what we have learned to develop a set of resources (a 'toolkit') to support referral in primary care, working collaboratively with patients and clinicians.

We request that the survey is completed by a member of clinical staff in your organisation who is most involved with receiving referrals and assessing respiratory patients.

Your responses are valuable to us and so your service will be paid £10 for the clinical staff time to complete it. In addition, we invite you to enter a prize draw to win £200 in online shopping vouchers as an incentive to take part. The project is registered on the NIHR CRN Portfolio and completion will count as an accrual for your organisation if you are within the NHS.

The survey should take about 10 to 20 minutes. Items include:

- Participant information and consent
- Your role
- Your pulmonary rehabilitation service
- Your experience of the PR referral process
- Improving referral and uptake of PR
- Getting in touch

There are no right or wrong answers. All responses and comments are helpful. At the end you will be able to leave any additional comments you wish to make.

On the next two pages you will find participant information about the survey and a consent form for your participation. Please complete the consent form before continuing.

Thank you for your participation.

I confirm that I am a member of clinical staff involved in receiving referrals and assessing patients for pulmonary rehabilitation

○ Yes

Page 2: Participant Information

You are invited to take part in an online survey as part of a project to help increase referral and uptake to Pulmonary Rehabilitation (PR) for people with Chronic Obstructive Pulmonary Disease (COPD).

1. What is the purpose of the study?

Our study aims to increase the number of people with COPD who attend PR by developing an online toolkit to help GP practices refer patients successfully. The National COPD Audit Programme reported that in 2013/14 an estimated 446,000 COPD patients in England and Wales were eligible for PR but that only 68,000 referrals were made. Of those, only 69% took up the referral offer.

This survey of PR providers is the first step in developing the toolkit. We are also surveying healthcare professionals (HCPs) in primary care who care for people with COPD. Following the survey we will hold interviews and focus groups with patients and HCPs to gather ideas about how to make referral easier and how to help patients make informed decisions about whether to attend PR. We will combine these ideas with evidence from published research and then work with patients and HCPs to assemble the information in an online toolkit. The toolkit could contain, for example, patient-friendly information, electronic reminders on patient records or simplified referral processes. It will be designed to integrate with primary care working practices and to be used, for example, during an annual COPD review. We will test the toolkit in primary care to ensure it is practical for the NHS and has the potential to make a difference.

2. Why have I been chosen?

We are inviting PR providers in the East of England to take part in the survey.

3. What will I need to do?

The survey will take about 10 to 20 minutes to complete and is designed to be quick and easy. It will ask about your experiences of referring patients to PR and for your ideas about how to improve referral and uptake. At the end of the survey, we will invite you to provide your contact details if you would like to take part in a subsequent interview or focus group on this topic.

4. How will the findings be used?

The survey results will be reported in a way that preserves confidentiality and you will not be identified in any way. The results may be published on the Addenbrooke's Hospital intranet, in peer reviewed medical journals, and used for medical presentations, conferences or presentations to patient groups. If you would like to be kept informed of the results please contact the Research Manager, Dr Frances Early, using the details below.

5. What will happen to the information I provide?

All information that is collected is strictly confidential. Only members of the research team will have access to the information. Where data are referred to in published material participants will not be identifiable. Data will be held in secure storage and destroyed after five years. Any information that you give will be used for research purposes only and you may ask to see your personal information at any time.

6. Can I withdraw from the project?

You may withdraw from the study at any time without giving a reason. If you withdraw we will only retain and use any personal information you have provided up to that point if you give us permission to do so.

7. Who has organised the research?

The Lead Investigator for the study is Dr Jonathan Fuld at Cambridge University Hospitals NHS Foundation Trust. Other members of the research team are:

Dr Frances Early (Cambridge University Hospitals NHS Foundation Trust)

Professor Christi Deaton, Dr Ian Wellwood, Dr John Benson, Dr Lois Kim (Cambridge Institute of Public Health)

Ruth Barlow, Lianne Jongepier (East of England Pulmonary Rehabilitation Network)

Professor Patricia Wilson (University of Kent)

Professor Sally Singh (University Hospitals of Leicester NHS Foundation Trust)

The British Lung Foundation

8. How has the study been funded?

The research is funded by the National Institute for Health Research as part of their Research for Patient Benefit programme (award no: PB-PG-1215-20034).

9. Further information and contact details

If you would like more information please contact Dr Frances Early, Box 146, Cambridge University Hospitals NHS Foundation Trust, Hills Road, Cambridge, CB2 0QQ. Tel 01223 274858. Email@ frances.early@addenbrookes.nhs.uk

(Participant information sheet WP2 A4 PIS Survey Pul Rehab v1.1 090717) IRAS ID 209597

Page 3: Participant Consent

Please tick each box below to indicate that you understand and agree with the statements

- ☐ I confirm that I have read and understood the above information sheet (WP2 A4 PIS Survey Pul Rehab v1.1 090717). I have had the opportunity to consider the information, ask questions and have had questions answered satisfactorily.
- ☐ I understand my participation is voluntary and I may withdraw at any time without giving any reason
- ☐ I agree that the information I provide can be used solely for the purpose of this research
- ☐ I agree to the use of the anonymised data in publications
- ☐ I understand that all personal information will remain confidential
- ☐ I agree to take part in the above study

I confirm my agreement to the above statements ✱ *Required*

Page 4: Your organisation

Please tell us the name and contact details of your PR provider organisation so that we can manage the payment for completion of this survey

Page 5: Please tell us about your role so that we can understand who is involved in delivering pulmonary rehabilitation

What best describes your professional job category?

- Physiotherapist
- Nurse
- Health support worker
- Health care assistant
- Occupational therapist
- Dietician
- Fitness instructor/trainer
- Pharmacist
- Other

If other, please give details

How many years have you been involved in the care of people with respiratory problems?

Please enter a whole number (integer).

What is your role in pulmonary rehabilitation? Please select all that apply.

- I manage the PR service
- I manage the PR team
- I run a PR class
- I assist with a PR class
- I work in the admin team and screen the PR referrals
- Other

If other, please give details

How many years have you worked in your pulmonary rehabilitation role?

Please enter a whole number (integer).

What is the WTE (Whole Time Equivalent) of your post?

What band or grade is your post?

Page 6: Please tell us about your pulmonary rehabilitation service

What type of organisation provides your PR service?

- ☐ NHS Trust or health board
- ☐ Community Interest Company (CIC)
- ☐ Private healthcare provider
- ☐ Charity
- ☐ Research
- ☐ Other

If other, please give details

Is your service open to referrals all year round?

- ○ Yes
- ○ No

Do you accept referrals from

- ○ Primary care
- ○ Secondary care
- ○ Primary and secondary care
- ○ Self-referral
- ○ Other

If other, please give details

What type of PR programmes do you offer?

- ☐ Rolling
- ☐ Cohort
- ☐ Rolling and cohort
- ☐ Other

If rolling, how many rolling programmes per year?

If cohort, how many cohorts per year?

If other, please give details

Do you offer group-based and/or one-to-one programmes?

- ☐ Group-based
- ☐ One-to-one
- ☐ Group-based and one-to-one
- ☐ Other

If other, please give details

In which settings does your service hold classes? Tick all that apply.

- ☐ Community settings
- ☐ Hospital settings
- ☐ Primary care settings
- ☐ Home settings
- ☐ Other

If other, please give details

At how many sites does your service offer PR?

Please enter a whole number (integer).

Do you offer initial assessment before enrolment on to PR?

- ○ Yes
- ○ No

How many exercise sessions do you offer for each course of PR (excluding assessment visits)?

Please enter a whole number (integer).

Page 7: Please tell us about the funding and capacity of your pulmonary rehabilitation service

How is your PR service funded?

- ☐ CCG commissioned
- ☐ Hospital funded
- ☐ Post-discharge rehabilitation tarrif
- ☐ Other

If other, please give details

Does your PR service have a fixed term of funding?

- ○ Yes
- ○ No
- ○ Don't know

If yes, how many years future funding does the service have? Please leave blank if you are not sure.

How many available places did your service have last year?

Please enter a number.

How many places did you fill in the last year (2016/17)?

Please enter a number.

What is the maximum number of places (across all sites) that your PR service can offer in the current financial year (2017-18) based on your staffing levels?

Please tick here if you are not sure

- ○ I'm not sure what the number of places is
- ○ I have given an estimate below

Estimated number of places

Please add any comments for clarification regarding funding or capacity

Page 8: Your experience of the pulmonary rehabilitation referral process (part i)

I understand the eligibility criteria for PR contained in the BTS Guideline on Pulmonary Rehabilitation in Adults

Please don't select more than 1 answer(s) per row.

	0	1	2	3	4	5	6	7	8	9	10	
Not at all	☐	☐	☐	☐	☐	☐	☐	☐	☐	☐	☐	Completely

The process of accepting patients onto a PR programme following referral is:

Please don't select more than 1 answer(s) per row.

	0	1	2	3	4	5	6	7	8	9	10	
Very difficult	☐	☐	☐	☐	☐	☐	☐	☐	☐	☐	☐	Very easy

I feel adequately prepared to accept patient referrals to PR

Please don't select more than 1 answer(s) per row.

	0	1	2	3	4	5	6	7	8	9	10	
Not at all	☐	☐	☐	☐	☐	☐	☐	☐	☐	☐	☐	Completely

Page 9: Your experience of the PR referral process (part ii)

Thinking of your work at the moment how strongly do you agree with the following statements?

Please don't select more than 1 answer(s) per row.

	Strongly disagree	Disagree	Not sure	Agree	Strongly agree
Staff in my organisation have a shared understanding of the purpose of PR	☐	☐	☐	☐	☐
There are key people in my organisation who promote PR	☐	☐	☐	☐	☐
I am open to working with colleagues in new ways to enable PR referral to happen	☐	☐	☐	☐	☐
I have confidence in clinicians' ability to refer patients to PR	☐	☐	☐	☐	☐
Work involved in processing PR referrals is assigned to people in my organisation who have the appropriate skills	☐	☐	☐	☐	☐
I feel I have had sufficient training to process referrals to PR	☐	☐	☐	☐	☐
I feel that sufficient resources are available in my organisation to support the processing of PR referrals	☐	☐	☐	☐	☐
My organisation provides adequate support for the PR referral process to enable it to be effective	☐	☐	☐	☐	☐
I am aware of audits or feedback reports within my organisation about the effectiveness of the PR referral process	☐	☐	☐	☐	☐
We adapt PR referral processes in light of feedback we receive	☐	☐	☐	☐	☐

Page 10: Your experience of the PR referral process (part iii)

What do you think are the main reasons that patients take up a referral to PR?

What do you think are the main reasons that patients who have been referred do not attend an assessment appointment?

Page 11: Communication and processes in pulmonary rehabilitation referral

Do you receive adequate communication from referrers about patients who have been referred to your service?

- ○ Yes
- ○ No

Please add comments to clarify if necessary

Which communication methods are effective for you when communicating with referrers? Select all that apply.

- ☐ Email
- ☐ Letter
- ☐ Telephone
- ☐ Other

If other, please give details

What do you think is important for effective communication between primary care and PR providers?

Do you send written reports to referrers about patients who have been referred to your service?

- ○ Yes
- ○ No

Please add comments to clarify if necessary

What tools, systems or processes are available in your organisation to support patients in their decision-making about whether to attend a PR assessment?

- ☐ Information about PR for patients
- ☐ Shared decision-making tools
- ☐ Other

If other please give details

Page 12: Improving referral and uptake to pulmonary rehabilitation

What do you think would make it easier for clinicians to refer patients to PR?

What do you think could be done to encourage patients to attend an assessment appointment following referral?

Page 13: Developing the pulmonary rehabilitation toolkit

Our research study aims to design and build a collection of resources (a 'toolkit') for use in primary care in order to support successful referral to PR. What do you think are the most important things that such a toolkit should contain or do in order for it to be acceptable and useful?

Page 14: Getting in touch

We are keen to gather examples of existing resources that may be useful to share through the toolkit. Do you have any resources that support the processing of PR referrals which you would be willing to share with the research team?

- Yes
- No

Would you like to enter the prize draw to win £200 of online shopping vouchers?

- Yes
- No

Thank you for completing this survey. All responses are confidential and results will be reported anonymously. Would you like us to share the results with you?

- Yes
- No

If you answered 'yes' to any of the above questions please leave your contact details here or, if you prefer to keep your responses anonymous, please email the research team directly: frances.early@addenbrookes.nhs.uk

Any other comments?

Page 15: The survey is now complete. Thank you for your time.

Key for selection options

3 - I confirm my agreement to the above statements

Yes
No
