# Supplementary material for: Developing an intervention to increase REferral and uptake TO pulmonary REhabilitation in primary care in patients with chronic obstructive pulmonary disease (the REsTORE study): mixed methods study protocol
Source: BMJ Open. 2019 Jan 21;9(1):e024806. doi: 10.1136/bmjopen-2018-024806 (PMC6347857; doi:10.1136/bmjopen-2018-024806)
Supplement: Supplementary data [file bmjopen-2018-024806supp003.pdf]

Supplement 3
REsTORE study focus group / interview schedule for HCPs in primary care

Perception of PR

1. How much do you know about PR?
2. How important is PR as treatment for COPD?
   a) Do you think PR is useful?
3. Do you think there are any differences in how you view PR and how other doctors, nurses and physiotherapists view it?
4. Is PR different from other parts of the COPD pathway? If yes – how?
   a) For example -Do you perceive it as being integral to the pathway?
   b) Are some components of the pathway are more important / effective / useful / acceptable?

Referral in practice

5. To what extent is PR referral part of your role?
6. What criteria do you use when referring people to COPD?
   a) How do you judge if it is worthwhile referring someone?
7. Can you describe what you do to refer someone to PR?
   a) Would you say there are differences in referral practice?
   b) Does everyone do it the same way?
   c) What tools/processes etc. are in place in your practice to ensure that PR referrals are made efficiently?
8. How would you describe the communication between you and the PR providers?
   a) For example, what types do you use and what is most effective - written, electronic/ telephone/person
9. How confident are you that referrals will be efficiently acted upon by other people in the pathway?
   a) Are there any efficiency standards for this?
10. Why do you think patients do or do not take up an offer?
11. Why do you think some HCPs might refer more people to PR than others?
    a) For example, differences between professionals or differences between practices i.e. variation within or between practices

Improving referral

12. What would make you more likely to refer to PR?
13. What would make it easier for you to refer to PR?
14. What would make it easier for patients to take up a referral?

Organisational support

15. Who drives PR referral forwards in your practice?
    a) What would make it easier to sustain levels of referrals?

Toolkit

16. Do you think a PR tool kit would be useful?
17. How would you judge whether a PR toolkit was worthwhile?
