# Supplementary material for: Developing an intervention to increase REferral and uptake TO pulmonary REhabilitation in primary care in patients with chronic obstructive pulmonary disease (the REsTORE study): mixed methods study protocol
Source: BMJ Open. 2019 Jan 21;9(1):e024806. doi: 10.1136/bmjopen-2018-024806 (PMC6347857; doi:10.1136/bmjopen-2018-024806)
Supplement: Supplementary data [file bmjopen-2018-024806supp004.pdf]

Supplement 4
REsTORE study focus group schedule for patients who have accepted a referral to PR

Introductions and welcome

1. **Do you know what Chronic Obstructive Pulmonary Disease (COPD) is?**
   a) Clarify understanding if patient is not sure.
   b) Offer COPD information leaflet.

2. **What is it like to live with COPD?**
   a) Can you tell me what you know about COPD and treatments?
   b) What do you have to do to manage your COPD, e.g. appointments, treatments, self-care…?
   c) How do you manage these things? Who or what helps you?
   d) Thinking about the demands of managing your COPD, what effect has this had on you and your life?

3. **Can you describe how you were referred to PR?**
   a) Who referred you?
   b) What did they tell you about the referral process or about PR, e.g. what PR is, why it might be a good idea for you? (PPI feedback indicated that this is a key question)
   c) How much did you know about PR before you attended?

4. **Why did you choose to attend PR?**
   a) Thinking about other people who could benefit from PR, what do you think they might need in order for them to be able to attend?
   b) Thinking about other people could benefit from PR, what do you think would make them want to attend?

5. **What things made it difficult for you to attend PR?**
   a) Challenges could be in their own life or in the healthcare system
   b) What do you have to do to be able to attend, e.g. change to routines, being open to trying something new…?
   c) How do you manage these things?
   d) Who or what helps you to attend PR?
   e) What would make attending PR easier?

6. **How has PR helped you compared to other treatments you are receiving for COPD?**
   a) How has attending PR affected you or your life (positive or negative)?
