# Supplementary material for: Developing an intervention to increase REferral and uptake TO pulmonary REhabilitation in primary care in patients with chronic obstructive pulmonary disease (the REsTORE study): mixed methods study protocol
Source: BMJ Open. 2019 Jan 21;9(1):e024806. doi: 10.1136/bmjopen-2018-024806 (PMC6347857; doi:10.1136/bmjopen-2018-024806)
Supplement: Supplementary data [file bmjopen-2018-024806supp005.pdf]

Supplement 5
REsTORE study focus group schedule for patients who have declined a referral to PR

1. **Do you know what Chronic Obstructive Pulmonary Disease (COPD) is?**
   a) Clarify understanding if patient is not sure.
   b) Offer COPD information leaflet.

2. **What is it like to live with COPD?**
   a) Can you tell me what you know about COPD and treatments?
   b) What do you have to do to manage your COPD, e.g. appointments, treatments, self-care…?
   c) How do you manage these things? Who or what helps you?
   d) Thinking about the demands of managing your COPD, what effect has this had on you and your life?

3. **Do you know what pulmonary rehabilitation (PR) is?**
   a) Clarify understanding if patient is not sure.
   b) Offer PR information leaflet.

4. **Can you describe how you were referred to PR?**
   a) Who referred you?
   b) What did they tell you about the referral process or about PR, e.g. what PR is, why it might be a good idea for you? (PPI feedback indicated that this is a key question)
   c) How much were you told about what happens in the PR classes?
   d) How much were you told about what you would need to do if you attended PR?

5. **Can you tell me the reasons why chose not to attend PR?**
   a) How did you decide whether or not it would help you?

6. **Were there any difficulties that put you off attending or made it impossible for you to attend PR?**
   a) Challenges could be in their own life or in the healthcare system
   b) What would you have had to do in order to attend, e.g. changed a routine, tried something unfamiliar …?

7. **What would make attending PR easier / what would help?**
   a) What would make attending PR more attractive?
   b) What kind of support might you need to attend PR?
   c) How would PR need to be different for you to want to attend?

8. **What do you know about how helpful PR could be compared to other treatments for COPD?**
   a) How important do you think PR is as a treatment?

Or, if interviewee believes that PR will be of no help to them:

9. **What other treatments are you on for COPD?**
   a) What do you think makes these more helpful in managing your COPD compared to PR?
