# Supplementary material for: Developing an intervention to increase REferral and uptake TO pulmonary REhabilitation in primary care in patients with chronic obstructive pulmonary disease (the REsTORE study): mixed methods study protocol
Source: BMJ Open. 2019 Jan 21;9(1):e024806. doi: 10.1136/bmjopen-2018-024806 (PMC6347857; doi:10.1136/bmjopen-2018-024806)
Supplement: Supplementary data [file bmjopen-2018-024806supp006.pdf]

Supplement 6
REsTORE study focus group schedule for patients who have not been referred to PR

1. Do you know what Chronic Obstructive Pulmonary Disease (COPD) is?

a) Clarify understanding if patient is not sure.
b) Offer COPD information leaflet.

2. What is it like to live with COPD?

a) Can you tell me what you know about COPD and treatments?
b) What do you have to do to manage your COPD, e.g. appointments, treatments, self-care…?
c) How do you manage this, who or what helps you?
d) Thinking about the demands of managing your COPD, what effect has this had on you and your life?

3. Do you know what pulmonary rehabilitation (PR) is?

a) Clarify understanding if patient is not sure.
b) Offer PR information leaflet.

4. Have you had any conversations with your nurse or GP about PR?

a) Who was the conversation with?
b) What was said?
c) What did they tell you about the referral process or about PR, e.g. what PR is, why it might be a good idea for you? (PPI feedback indicated that this is a key question)
d) What do you know about what PR involves?
e) If patient does not know about PR, offer information about PR (British Lung Foundation leaflets)

5. Would you like to be referred to PR?

If YES or NOT SURE go to question 6. If NO go to question 8.

6. If yes or not sure to question 5: What do you know about how helpful PR could be compared to other treatments for COPD?

a) How important do you think PR is as a treatment for COPD? [Note that this question may depend on why there were not referred]
b) What might you want from a programme like PR (i.e. an exercise and education programme) that would help you to manage your COPD?

7. What sort of problems or obstacles, if any, would you need to overcome to be able to attend a PR course?

a) Challenges could be in their own life or in the healthcare system
b) What do you think you would have to do in order to be able to attend, e.g. change routines, try something unfamiliar…?

8. What would make attending PR easy / what would help?

a) What would make attending PR attractive?
b) What kind of support might you need to attend PR?

END OF QUESTIONS

9. If no to question 5: Can you tell me the reasons why you would not want to be referred?

a) Can you tell me about whether you think it would or would not help you?

10. Are there any difficulties that would put you off attending or make it impossible for you to attend PR?

a) Challenges could be in their own life or in the healthcare system
b) What do you think you would have to do in order to be able to attend, e.g. change a routine, try something unfamiliar…?
c) What would make attending PR easy / what would help?
d) What would make attending PR attractive?
e) What kind of support might you need to attend PR?
f) How would PR need to be different for you to want to be referred?

END OF QUESTIONS
