# Supplementary material for: Developing an intervention to increase REferral and uptake TO pulmonary REhabilitation in primary care in patients with chronic obstructive pulmonary disease (the REsTORE study): mixed methods study protocol
Source: BMJ Open. 2019 Jan 21;9(1):e024806. doi: 10.1136/bmjopen-2018-024806 (PMC6347857; doi:10.1136/bmjopen-2018-024806)
Supplement: Supplementary data [file bmjopen-2018-024806supp007.pdf]

Supplement 7
REsTORE study focus group schedule for PR providers

Perception of PR

1. What do you think is the value of PR as a treatment for COPD?
   a) Explore any differences in how its value is perceived
   b) Why do you think some patients take up an offer but others don't?

Referral in practice

2. Can you describe what you do to process a PR referral?
   a) What tools/processes etc. are in place in your organisation to ensure that PR referrals are processed efficiently?
   b) What would make it easier for you to efficiently process referrals?
   c) What could be done in your organisation to make efficient processing of PR referral easier to sustain?

3. How effective is communication between referrers and PR providers?

4. In your experience why do you think some HCPs refer more patients to PR than others?

Improving referral

5. What could be done to increase referral to PR?
6. What could be done to increase uptake to PR?

Toolkit (Introduce what a toolkit is)

7. How would you judge whether a PR referral toolkit in primary care was worthwhile?
