# Supplementary material for: Developing an intervention to increase REferral and uptake TO pulmonary REhabilitation in primary care in patients with chronic obstructive pulmonary disease (the REsTORE study): mixed methods study protocol
Source: BMJ Open. 2019 Jan 21;9(1):e024806. doi: 10.1136/bmjopen-2018-024806 (PMC6347857; doi:10.1136/bmjopen-2018-024806)
Supplement: Supplementary data [file bmjopen-2018-024806supp008.pdf]

Supplement 8
REsTORE study interview schedule for commissioners

Perception of PR

1. How much do you know about PR?
   a) How valuable do you think PR is as a treatment for COPD?

2. Is PR different from other parts of the COPD pathway? If yes, how?

Referral in practice and how you support PR providers in making referrals

3. How confident are you as a commissioner that the PR referral pathway is performing efficiently?
   a) What tools or processes are in place in your organisation to support practices in making sure that PR referrals are made efficiently?

4. To what extent is it part of your role to support PR referral and utilisation in primary care?
   a) How do commissioners support practitioners in their assessment of whether or not to refer people to PR?

5. Why do you think some HCPs might refer more people to PR than others?
6. Why do you think patients do or don't take up an offer of PR?

Improving referral

7. What could commissioners do to make it easier for practices to successfully refer to PR?
8. What would make it easier for patients to take up a referral?

Support within your organisation

9. Who or what drives the requirement for PR utilisation in primary care forwards in your organisation?
10. In your organisation, what would make support for PR utilisation easier to sustain?

Toolkit

11. Do you think a PR referral toolkit would be useful?
12. How would you judge whether a PR referral toolkit was worthwhile?
