# Supplementary material for: Developing an intervention to increase REferral and uptake TO pulmonary REhabilitation in primary care in patients with chronic obstructive pulmonary disease (the REsTORE study): mixed methods study protocol
Source: BMJ Open. 2019 Jan 21;9(1):e024806. doi: 10.1136/bmjopen-2018-024806 (PMC6347857; doi:10.1136/bmjopen-2018-024806)
Supplement: Author's manuscript [file bmjopen-2018-024806.draft_revisions.pdf]

BMJ Open is committed to open peer review. As part of this commitment we make the peer review history of every article we publish publicly available.

When an article is published we post the peer reviewers' comments and the authors' responses online. We also post the versions of the paper that were used during peer review. These are the versions that the peer review comments apply to.

The versions of the paper that follow are the versions that were submitted during the peer review process. They are not the versions of record or the final published versions. They should not be cited or distributed as the published version of this manuscript.

BMJ Open is an open access journal and the full, final, typeset and author-corrected version of record of the manuscript is available on our site with no access controls, subscription charges or pay-per-view fees (http://bmjopen.bmj.com).

If you have any questions on BMJ Open's open peer review process please email info.bmjopen@bmj.com

BMJ Open

Developing an intervention to increase REferral and uptake TO pulmonary REhabilitation in primary care in patients with Chronic Obstructive Pulmonary Disease (the REsTORE study): mixed methods study protocol

Journal:	*BMJ Open*
Manuscript ID	bmjopen-2018-024806
Article Type:	Protocol
Date Submitted by the Author:	14-Jun-2018
Complete List of Authors:	Early, Frances; Cambridge University Hospitals NHS Foundation Trust, Box 146<br>Wilson, Patricia; University of Kent, Kent Academic Primary Care Unit<br>Deaton, Christi; University of Cambridge, Cambridge Institute of Public Health, School of Clinical Medicine<br>Wellwood, Ian; University of Cambridge, Cambridge Institute of Public Health, School of Clinical Medicine<br>Dickerson, Terry; University of Cambridge, Engineering Design Centre<br>Ward, James; University of Cambridge, Engineering<br>Jongepier, Lianne; NHS North East Essex Clinical Commissioning Group<br>Barlow, Ruth; Provide Community Interest Company<br>Singh, Sally; University Hospitals of Leicester NHS Trust, Cardiac/Pulmonary Rehabilitation<br>Benson, John; University of Cambridge, GP and Primary Care Research Unit<br>Brimicombe, James; University of Cambridge, Primary Care Unit, Department of Public Health and Primary Care; University of Cambridge<br>Kim, Lois; University of Cambridge Department of Public Health and Primary Care<br>Haque, Hena; Cambridge University Hospitals NHS Foundation Trust, Centre for Self Management Support<br>Fuld, Jonathan; Cambridge University Hospitals NHS Foundation Trust, Centre for Self Management Support
Keywords:	Quality in health care < HEALTH SERVICES ADMINISTRATION & MANAGEMENT, PRIMARY CARE, QUALITATIVE RESEARCH, REHABILITATION MEDICINE, RESPIRATORY MEDICINE (see Thoracic Medicine), Chronic airways disease < THORACIC MEDICINE

SCHOLARONE™
Manuscripts

Developing an intervention to increase REferral and uptake TO pulmonary REhabilitation in primary care in patients with Chronic Obstructive Pulmonary Disease (the REsTORE study): mixed methods study protocol

Corresponding author:

Dr Frances Early, Centre for Self Management Support, Box 146, Cambridge University Hospitals NHS Foundation Trust, Cambridge Biomedical Campus, Hills Road, Cambridge CB2 0QQ. Email: Frances.early@addenbrookes.nhs.uk. Tel: 01223 274858.

Authors:

Frances Early (corresponding author)

Patricia Wilson, Centre for Health Services Studies, University of Kent, Canterbury CT2 7NF. UK.

Christi Deaton, Department of Public Health and Primary Care, University of Cambridge School of Clinical Medicine, Box 111 Cambridge Biomedical Campus, Cambridge, CB2 0SP. UK

Ian Wellwood, Department of Public Health and Primary Care, University of Cambridge School of Clinical Medicine, Box 111 Cambridge Biomedical Campus, Cambridge, CB2 0SP. UK

Terry Dickerson, Department of Engineering, University of Cambridge, Trumpington Street, Cambridge, CB2 1PZ. UK

James Ward, Department of Engineering, University of Cambridge, Trumpington Street, Cambridge, CB2 1PZ. UK

Lianne Jongepier, NHS North East Essex Clinical Commissioning Group, Aspen House, Stephenson Road, Severalls Business Park, Colchester, CO4 9QR. UK

Ruth Barlow, Provide Community Interest Company, 900 The Crescent, Colchester Business Park, Colchester, Essex, CO4 9YQ. UK

Sally Singh, University Hospitals of Leicester NHS Trust, Cardiac/Pulmonary Rehabilitation Glenfield Hospital, Groby Rd, Leicester, LE3 9QP. UK

John Benson, Department of Public Health and Primary Care, University of Cambridge School of Clinical Medicine, Box 111 Cambridge Biomedical Campus, Cambridge, CB2 0SP. UK

James Brimicombe, Department of Public Health and Primary Care, University of Cambridge School of Clinical Medicine, Box 111 Cambridge Biomedical Campus, Cambridge, CB2 0SP. UK

Lois Kim, Department of Public Health and Primary Care, University of Cambridge School of Clinical Medicine, Box 111 Cambridge Biomedical Campus, Cambridge, CB2 0SP. UK

Hena Haque, Centre for Self Management Support, Box 146, Cambridge University Hospitals NHS Foundation Trust, Cambridge Biomedical Campus, Hills Road, Cambridge CB2 0QQ. UK

Jonathan Fuld, Centre for Self Management Support, Box 146, Cambridge University Hospitals NHS Foundation Trust, Cambridge Biomedical Campus, Hills Road, Cambridge CB2 0QQ. UK

Word count: 3,452

For peer review only - http://bmjopen.bmj.com/site/about/guidelines.xhtml

Keywords

Quality in healthcare, primary care, qualitative research, rehabilitation medicine, respiratory medicine, chronic airways disease

For peer review only

For peer review only - http://bmjopen.bmj.com/site/about/guidelines.xhtml

Abstract

Introduction: Chronic Obstructive Pulmonary Disease (COPD) is a progressive lung disease associated with breathlessness, inability to exercise, frequent infections, hospitalisation and reduced quality of life. Pulmonary rehabilitation (PR), providing supervised exercise and education, is an effective and cost-effective treatment for COPD but is significantly underutilised. Interventions to improve referral and uptake have been tested and some positive results reported. However, interventions are diverse and no clear recommendations for practice can be made. This study aims to understand the challenges to referral and uptake in primary care, where most referrals originate, and to develop a flexible toolkit of resources to support referral and uptake to PR in primary care.

Methods and analysis: This is a mixed methods study informed by Normalisation Process Theory. In the first phase GPs, practice nurses and PR providers will be invited to complete an online survey to inform a broad exploration of the topic areas. In phase 2 interviews and focus groups will be conducted with patients, healthcare professionals (HCPs) in primary care, PR providers and commissioners to gain an in-depth understanding of the issues and needs. Toolkit development in phase 3 will draw together the learning from phases 1 and 2 and employ an iterative development process to build the toolkit jointly with patients and HCPs. It will be tested in primary care for usability and acceptability.

Ethics and dissemination: The study has ethical and Health Research Authority approval (Research Ethics Committee reference number 17/EE/0136). It is registered with the International Standard Registered Clinical/soCial sTudy Number (ISRCTN) registry (trial ID: ISRCTN20669629). Dissemination will be aimed at patients, carers/families, service providers, commissioners and national interest groups. Methods will include conferences, presentations, academic publications and plain English reports and will be supported by the British Lung Foundation.

Strengths and limitations of this study

- The study explicitly uses Normalisation Process Theory to inform data collection methods, data analysis and toolkit development which will maximise potential for the toolkit to be successfully implemented in practice.
- Patients who are unable to communicate in English, Hindi or Urdu will be excluded from the study.
- Patients who have declined PR may be challenging to engage and so we will work closely with primary care to recruit this group.
- An iterative design process enables rapid development of the toolkit in a way that readily allows it to be refined and tailored to the needs of users and the overall system, resulting in a design of higher quality which is more likely to stand the test of time.
- Outputs from survey and qualitative data will support identification of design priorities at the start of development which will provide focus for the iterative process.

For peer review only - http://bmjopen.bmj.com/site/about/guidelines.xhtml

INTRODUCTION

Chronic Obstructive Pulmonary Disease (COPD) is a progressive lung disease associated with breathlessness, inability to exercise, frequent infections and hospitalisation. Approximately 1.2 million people were living with diagnosed COPD in England in 2012[1] and annual direct healthcare costs of COPD in England have been estimated to increase from £1.50 billion in 2011 to £2.32 billion in 2030.[2] Pulmonary rehabilitation (PR), providing supervised exercise and education, is an effective treatment for COPD that leads to clinically significant improvements in exercise capacity, symptoms and health-related quality of life in patients who experience disabling breathlessness.[3] PR also supports self-management skills[4] and results in fewer and shorter hospital admissions[5] and readmissions.[6] It is a cost-effective treatment[7] recommended for patients who are functionally disabled by COPD.[8]

However, despite published guidelines,[8-9] PR is significantly underutilised. In England and Wales in 2013/14 the estimated prevalence of COPD patients eligible for PR was 446,000 but only 68,000 referrals were received by PR programmes. Of those patients referred, 31% did not attend for assessment.[10] There is an urgent need to review PR referral pathways, healthcare professional (HCP) training, information for patients and referrers, and barriers to patient access, particularly in primary care where 51% of referrals originate.[10]

Referral rates are impacted by difficult referral processes, lack of information about PR and unclear roles and responsibilities regarding referral.[11-24] Uptake is impacted by the quality of the HCP conversation about PR and patients' beliefs about the benefits, as well as timing, location and transport.[25-27] Interventions to improve referral and uptake have been tested[28-41] and some positive results reported[33-34,36,38-40] although study designs are diverse and there are no clear recommendations for practice. This paper presents the protocol for a mixed methods study to support the improvement of referral and uptake to PR from primary care. The study runs from August 2017 to December 2018.

Aims and objectives

The aim is to understand the needs of HCPs and patients in general practice concerning the challenges to referral and uptake, and then use this understanding to develop a flexible toolkit of resources to support referral and uptake of PR within primary care.

The research questions (RQ) are:

1. From HCPs and patients' perspectives how can barriers to PR referral and uptake be overcome and facilitators of utilisation be supported?
2. What components do HCPs and patients believe should be included in a toolkit to increase PR referral and uptake?
3. How acceptable and usable is the toolkit for HCPs and patients as a means to increase referral and uptake of PR?

Objectives to address the research questions are:

a) Conduct empirical research to identify factors that HCPs and patients believe will overcome barriers and facilitate referral and uptake to PR in primary care.
b) Synthesise evidence from empirical research and our systematic literature review to develop the toolkit collaboratively with patients and providers.

For peer review only - http://bmjopen.bmj.com/site/about/guidelines.xhtml

c) Conduct exploratory efficacy testing of the toolkit in a sample of primary care practices to assess performance and gather data to provide a basis for future economic analysis.

Guiding theory

The research design is informed by Normalisation Process Theory (NPT),[42-43] an established theoretical framework for understanding implementation processes through multiple stakeholder perspectives. NPT will provide the theoretical underpinning for data collection, analysis and interpretation, and toolkit development. The four key constructs of NPT will be applied to the PR context to understand how PR referral becomes embedded in clinical practice:

a) Coherence: how PR is understood by clinicians.
b) Cognitive participation: clinicians' buy-in and support for PR.
c) Collective action: the operational work required for PR referral.
d) Reflexive monitoring: how PR is appraised by clinicians and how work is reconfigured to enable it to happen.

Closely aligned to NPT, Burden of Treatment (BoT) Theory will be used to understand patients' expeiences.[44] BoT provides a structural model of the relationship between treatment burden and patients'/carers' capacity to undertake work required to manage their condition, and helps to understand variations in healthcare utilisation and adherence.[45] Health service responses to reduce this burden will be understood through the Minimally Disruptive Medicine (MDM) model, a patient-centred approach in which patients and professionals work together to advance patient goals whilst imposing the smallest possible treatment burden.[46] Key elements of BoT and MDM mirror those of NPT and will inform understanding of how patients:

a) Make sense of their health conditions, tests and treatments.
b) Enrol support, plan to attend healthcare visits and enact self-care activities.
c) Operationalise the work of attending visits and self-managing their care.
d) Monitor, appraise and evaluate the worth of the work they are doing.

METHODS AND ANALYSIS

Study overview

The study comprises three phases:

Phase 1: Online survey of HCPs in general practice and online survey of PR providers
Phase 2: Qualitative research with patients, HCPs and commissioners
Phase 3: Toolkit development research

Figure 1 shows how the phases map to the research questions.

Phases 1 and 2 will address the following topic areas to identify facilitators and barriers to referral and uptake and explore current practice and ideas for improvement:

- Influences on HCP decisions to refer to PR
- Reasons why patients take up or decline a referral

For peer review only - http://bmjopen.bmj.com/site/about/guidelines.xhtml

- Methods to make it easier to refer to PR and take up a referral
- Tools/strategies in use to prompt referral and uptake
- Support or training that would improve counselling of patients regarding PR
- Ways to ensure effective communication between primary care and PR providers regarding referrals
- Priorities and design considerations that the toolkit should address

Findings from Phases 1 and 2 will inform toolkit development in Phase 3. The final study output will be a toolkit tested in clinical practice for usability and acceptability which, if validated, will be ready for wider testing.

Phase 1: Online surveys of HCPs in general practice and of PR providers

The surveys enable a broad exploration of the topic areas. Pulmonary disease or long-term condition leads in all GP practices in the East of England (EoE) (n = approximately 455) will be invited to complete the general practice survey by the Clinical Research Network (CRN) Eastern. The study team will send invitations to all PR providers (n = 21) in the EoE PR Network to complete the PR provider survey.

Survey design is informed by NPT and includes items adapted from the NoMAD tool.[47] We sought input to the design from practice nurses and PR providers and piloted each survey. Items are dichotomous, multiple response or Likert scales, supplemented by free text questions [see supplements 1 and 2]. We will invite HCPs to upload relevant resources that they recommend. Surveys will be implemented on the Online Surveys platform[48] and a prize draw of £200 in online shopping vouchers will incentivise returns.

Quantitative data will be uploaded to SPSS (v 25) for descriptive analysis including frequencies and comparison of responses by different healthcare providers and levels of experience. General practice data will be compared by Clinical Commissioning Group (CCG) area and mapped onto referral and uptake data from PR services where this is available.

Free text responses will be imported into NVivo 12 software. Analysis will follow a Framework approach.[49] NPT constructs will inform a deductive coding structure supplemented by inductive coding to allow unexpected findings to emerge. Codes will be grouped into categories and a working analytical framework developed which will be applied to all data. Data will be summarised by category in framework matrices on which the final interpretation will be based.

Phase 2: Qualitative research with patients, HCPs and commissioners

The purpose of the qualitative research is to enable an in-depth exploration of the topic areas with the following groups:

1. Patients who have accepted a PR referral (focus groups)
2. Patients who have declined a PR referral (interviews)
3. Patients who have not been referred to PR (interviews)
4. HCPs in primary care who refer patients to PR (interviews and/or focus groups)
5. Commissioners of PR services (interviews)
6. Physiotherapists and/or nurses who deliver PR (focus group)

Inclusion criteria

For peer review only - http://bmjopen.bmj.com/site/about/guidelines.xhtml

Patients will be eligible if they are resident in the East of England, have a diagnosis of COPD with stable disease, are eligible for PR as defined by guideline recommendations[8] and able to read and write in English. Patients who have accepted a PR referral will be eligible if they have attended only one programme. In addition, we will conduct interviews and focus groups in two geographical areas specifically targeting patients of South Asian heritage. For these activities patients will be eligible if they do not have the ability to read or write in English but can communicate, read and write in Hindi or Urdu.

Sampling

Patients and HCPs: We will identify four PR services within the CRN Eastern area based on utilisation of commissioned places in 2014/15, selecting the two services with the highest utilisation rates and the two with the lowest utilisation rates. We will then use convenience sampling within the PR services and the general practices that refer to them to recruit patients who have accepted a PR referral, patients who have declined or not been referred to PR and HCPs in primary care who refer patients to PR. We will also select two geographical areas in the East of England that have populations of South Asian heritage. We will use convenience sampling within the PR services and general practices to identify patients of South Asian heritage who have accepted a PR referral, patients who have declined or not been referred to PR and HCPs in primary care who refer patients to PR.

Commissioners: We will use convenience sampling to identify a commissioner of each of the four PR services.

PR providers: We will use convenience sampling within the EoE PR Network to identify PR providers across the region.

To enhance validity, the final sample size will be determined by the need to reach data saturation. In a study of patients referred to PR Arnold et al.[50] achieved saturation with 24 patients. Table 1 shows an estimation of the sample sizes to be aimed for. If necessary more participants will be sought in order to achieve data saturation or the final number may be lower if saturation is achieved with a lower number.

**Participant group**	**Estimated sample size**
Patients who have accepted a PR referral (focus groups)	Up to 48
Patients who have declined a PR referral (interviews)	Up to 9
Patients who have not been referred to PR (interviews)	Up to 9
HCPs in primary care who refer patients to PR (interviews and/or focus groups)	Up to 34
Commissioners of PR services (interviews)	4
Physiotherapists and/or nurses who deliver PR (focus group)	Up to 10

Table 1: Estimated sample sizes for each group of participants

Recruitment

Patients who have accepted a PR referral: Each PR service will identify eligible patients, issue the invitation to attend the focus group and confirm attendance.

Patients who have declined a PR referral and who have not been referred to PR: General practices will identify patients from their COPD register and the invitation will be made by a clinician known to the

For peer review only - http://bmjopen.bmj.com/site/about/guidelines.xhtml

patient. Telephone interviews, home visits or an interview on general practice premises will be offered to encourage participation. Interested patients will agree to be contacted by the study team who will make arrangements for interview.

HCPs in primary care: CRN Eastern will invite HCPs to participate. Interested practices will return an expression of interest form to CRN. CRN will inform the study team who will arrange the interview or focus group.

Commissioners of PR services: The study team will identify and invite commissioners with support from CRN Eastern.

Physiotherapists/nurses who deliver PR: The study team will invite participation through the EoE PR network.

For peer review only

Informed consent

Study team researchers will take written informed consent prior to commencing interviews or focus groups. Consent will also be taken from carers, family members or friends who accompany participants as they may provide information about the participant during the course of the interview or focus group. Participants will be free to withdraw from the study at any time.

Data collection

Question guides for interviews and focus groups [supplements 3-8] are developed from the relevant topic areas. Patient focus groups will be held at community or health care venues and interviews will be at the patient's home, a health care venue or conducted by telephone. Focus groups in general practice may be challenging to organise due to competing work schedules and geographical location so we will offer one-to-one interviews as well, on general practice premises or by telephone. The PR provider focus group will be held at a health care venue and commissioner interviews at the commissioner's place of work or by telephone. Interviews and focus groups will be audio-recorded and transcribed verbatim.

Data analysis

Transcriptions and field notes will be imported into NVivo 12 software for data management and analysis. Following a Framework approach[49] NPT constructs will inform a deductive coding structure supplemented by inductive coding to allow unexpected findings to emerge. Scripts will be read, re-read and coded by two researchers. Codes will be grouped into categories and a working analytical framework developed which will be applied to all transcripts. Data will be summarised by category in framework matrices on which the final interpretation will be based.

Quality criteria

Structured and standardised question guides will ensure consistency. Transcripts will be coded independently by two researchers. Reviewing and revising the analysis with a patient reference group will provide a credibility and reliability check.

Phase 3: Toolkit Development Research

The purpose of this phase is to synthesise findings from Phases 1 and 2 and incorporate the learning into the toolkit development.

For peer review only - http://bmjopen.bmj.com/site/about/guidelines.xhtml

User group

A user group of five HCPs and five patients recruited from research participants, volunteers from PPI events and British Lung Foundation (BLF) networks will work with the study team, a web developer and an expert in design and development from the Engineering Design Centre at the University of Cambridge to develop the toolkit. We will offer a range of ways to participate in testing and feedback, e.g. online, face-to-face meetings, visiting members, holding satellite meetings at professional conferences or service/training meetings. Written informed consent will be taken for all participants.

The design process

A rigorous, iterative design process (Explore, Create, Evaluate)[51] (figure 2) will track the user journey from initial concept to building the toolkit, including acceptability and usability testing.

To explore potential toolkit content and structure the needs, a review will take place of the summaries of the themes identified from the survey and qualitative research in phase 2. This will be underpinned by NPT and BoT constructs, and will be supported further by the outputs from the literature review and work by other expert groups e.g. the British Thoracic Society PR Quality Improvement Group. The toolkit will be simple and practical with information about ways to impact referral and uptake (examples may include key messages, electronic prompts/templates, links to guidelines and decision-support tools and printing options). A consensus method adapted from nominal group technique[52] will be used to consider and vote on priorities. Design will initially be solution-neutral and will then be translated into practical solutions. Practical resource constraints will be considered and any user-defined needs that could not be addressed will be recorded. The group will also agree initial assessment criteria for usability and acceptability testing.

Agile methods[53] will be used to create the toolkit. The study team will build initial web pages in the live location to develop concepts and stimulate ideas. Early and subsequent releases of the toolkit will be continuously explored, created, evaluated and fed back upon with the user group, alongside continuous web development in short time cycles over six months. Usability and acceptability assessment criteria will determine the feedback requested and with each round of feedback the criteria will be open to further refinement as users explore and identify their practical needs. The toolkit will be built on a WordPress platform, a free open source website infrastructure and content management system, and hosted on University of Cambridge web servers by University Information Systems. A web development expert from the University of Cambridge will lead the technical development. It will be designed to interface with working systems (in ways to be identified during the research, e.g. linked through SystmOne,[54] integrated into annual review) and a flexible design will enable on-going development.

Data collection

During toolkit development subjective and objective data will be gathered from user group participants to assess the user-defined usability and acceptability criteria. Examples may include user observation, think aloud technique, questionnaires and interviews.

Following development we will train practice nurses in five general practices to use the toolkit for four weeks in a clinical setting. Feedback will be sought relating to the user-defined assessment criteria plus user experience, perceived understanding of PR and referral criteria, technical issues and nurse time to implement the toolkit. Each nurse will complete a short questionnaire following use of

For peer review only - http://bmjopen.bmj.com/site/about/guidelines.xhtml

the toolkit with patients and an interview at the end of the four week period. Patients with whom the nurse has used the toolkit will complete a short evaluation questionnaire to assess the impact on the patient experience. This will be returned directly to the study team with a stamped addressed envelope. Questionnaires will reflect the evaluation criteria. Written informed consent will be taken from nurses and patients. During this testing period we will collect usage metrics from the system and data on referral numbers and uptake compared to baseline.

Data analysis

Quantitative data will be uploaded into Excel and descriptive analysis will be conducted to identify frequencies. Interviews will be audio recorded, transcribed and uploaded to NVivo 12 software for data management and analysis. Data analysis will follow procedures as described in Phase 2 with the addition that the user-defined usability and acceptability criteria, together with NPT constructs, will inform the development of a deductive coding structure.

PATIENT AND PUBLIC INVOLVEMENT

We involved a range of people in PPI activities to inform the study design and plain English communications. Forty-seven people from PR programmes and patient support groups attended across six meetings, we consulted four members of the Cambridge University Hospitals PPI panel and noted feedback from patient participants in meetings about the future of services throughout the Cambridgeshire and Peterborough healthcare system. A need for increased access to PR and the value of locating the study in primary care were seen as important. As a result we engaged a GP co-applicant to the study. PPI input is ongoing and we have established a PPI group, supported by a member of the study team, who will review research materials, feedback on the research analysis and support dissemination. We offer travel expenses to PPI meetings. The British Lung Foundation will also support active engagement of their patient members.

We also sought feedback on the research priorities from practice nurses at professional development meetings. They wanted easy access to good quality information and methods to engage patients, and recommended an emphasis on patient benefit, rather than cost savings, to encourage practice nurses to participate.

ETHICS AND DISSEMINATION

The study has been given a favourable opinion by the Cambridge Central Research Ethics Committee (REC reference number 17/EE/0136) and has been granted approval by the Health Research Authority. It is registered with the ISRCTN registry (trial ID: ISRCTN20669629).

The study involves minimal risks to patients; however patients may be vulnerable due to age or frailty and the nature of COPD symptoms, including breathlessness and fatigue. Participants will be able to take breaks and terminate an activity if they are unable to continue. We will enquire about special support needs and participants can bring a friend or carer to any activity. Experienced researchers with appropriate training will undertake interviews and focus groups.

Dissemination plan

We aim for timely dissemination, parallel to formal evaluation and clarifying how the outcomes will be of practical benefit to patients. Audiences include: patients and carers/families; HCPs who refer to

For peer review only - http://bmjopen.bmj.com/site/about/guidelines.xhtml

For peer review only

PR and general practice managers; PR providers and service managers; PR commissioners; national interest groups, e.g. British Thoracic Society (BTS); NHS England and NHS Improvement.

We will use a range of channels, working closely with our co-applicant the British Lung Foundation to utilise patient networks, publications, online information, service development and HCP engagement. We will use routes that are accessible to patient audiences, e.g. Breathe Easy patient newsletters, INVOLVE website, posters in GP waiting rooms and PR classes, village newsletters. PPI volunteers will disseminate to patient groups and networks.

Methods will include: presentations and written information for HCP forums and professional networks; presentations at regional, national and international academic and professional conferences; open access academic papers in peer-reviewed journals; web and social media updates; written feedback to participants; broadcast and print media, e.g. radio and newspapers, may be considered if appropriate.

Data deposition and curation

The study complies with the General Data Protection Regulation (GDPR) (2018). Data will be anonymised and entered onto secure NHS computers and University of Cambridge computers for data analysis. Signed, paper consent forms containing participants' names will be stored in a locked filing cabinet on NHS premises. Only the study team will have access to participants' data. Electronic files will be password protected. Data sharing and storage will meet the requirements of the National Institutes of Health Research. Data will be securely stored in the University of Cambridge Research Repository.

For peer review only

For peer review only - http://bmjopen.bmj.com/site/about/guidelines.xhtml

ACKNOWLEDGEMENTS

We would like to thank the patients and their families who contributed to the PPI work that supported the development of this study. Thanks to Jenna Stockwell from the British Lung Foundation who is supporting the study and its implementation. Delivery of this work was supported by the Cambridge Biomedical Research Centre.

COMPETING INTERESTS STATEMENT

JW reports grants from National Institute for Health Research during the conduct of the study.

No further disclosures.

FUNDING STATEMENT

This paper presents independent research funded by the National Institute for Health Research (NIHR) under its Research for Patient Benefit (RfPB) Programme (Grant Reference Number PB-PG-1215-20034). The views expressed are those of the authors and not necessarily those of the NHS, the NIHR or the Department of Health.

For peer review only

AUTHOR STATEMENT

JF led the research team, supervised the development of the protocol, contributed to the design of the study and development of the protocol, and revised the draft manuscript. He is a guarantor for the work.

FE contributed to the design of the study and development of the protocol, and drafted and revised the manuscript.

PW contributed to the design of the study and development of the protocol, proposed the theoretical basis for the design and supervised development of the associated methodology and revised the draft manuscript.

CD contributed to the design of the study and development of the protocol and revised the draft manuscript. She is a guarantor for the work.

IW contributed to the design of the study and development of the protocol and revised the draft manuscript.

TD contributed to the design of the study and development of the protocol and proposed the toolkit development methodology.

JW contributed to the design of the study and development of the protocol, proposed the toolkit development methodology and revised the draft manuscript.

LJ contributed to the design of the study and development of the protocol.

RB contributed to the design of the study and development of the protocol.

SS contributed to the design of the study and development of the protocol.

JBenson contributed to the design of the study and development of the protocol.

For peer review only - http://bmjopen.bmj.com/site/about/guidelines.xhtml

JBrimicombe provided technical advice regarding building and using the platform for developing the toolkit and revised the draft manuscript.

LK contributed to the design of the study and development of the protocol.

HH contributed to the design of the study and development of the protocol and revised the draft manuscript.

For peer review only

For peer review only - http://bmjopen.bmj.com/site/about/guidelines.xhtml

REFERENCES

1. British Lung Foundation. Chronic Obstructive Pulmonary Disease (COPD) statistics http://statistics.blf.org.uk/copd. Accessed June 2018.
2. McLean S, Hoogendoorn M, Hoogenveen RT, et al. Projecting the COPD population and costs in England and Scotland: 2011 to 2030. *Sci Rep*. 2016;6:31893.doi:10.1038/srep31893.
3. McCarthy B, Casey D, Devane D et al. Pulmonary rehabilitation for chronic obstructive pulmonary disease (Review). *Cochrane Database Syst Rev* 2015(2):p.Art.No.:CD003793.doi:10.1002/14651858.CD003793.pub3.
4. Singh SJ, Zuwallack R, Garvey C et al. Learn from the past and create the future: the 2013 ATS/ERS statement on pulmonary rehabilitation. *Eur Respir J* 2013;42(5):1169-74.doi:10.1183/09031936.00207912.
5. Griffiths TL, Burr ML, Campbell IA et al. Results at 1 year of outpatient multidisciplinary pulmonary rehabilitation: a randomised controlled trial. *Lancet* 2000;355:362-368.doi:10.1016/S0140-6736(99)07042-7.
6. Seymour JM, Moore L, Jolley CJ et al. Outpatient pulmonary rehabilitation following acute exacerbations of COPD. *Thorax* 2010;65(5):423-428.doi:10.1136/thx.2009.124164.
7. Griffiths TL, Phillips CJ, Davies S, et al Cost effectiveness of an outpatient multidisciplinary pulmonary rehabilitation programme. *Thorax* 2001;56:779-784.doi :10.1136/thorax.56.10.779
8. National Institute for Health and Clinical Excellence. COPD Guideline CG101 2010 http://www.nice.org.uk/guidance/CG101. Accessed June 2018.
9. Bolton CE, Bevan-Smith EF, Blakey JD et al. The BTS Guideline on Pulmonary Rehabilitation in Adults. *Thorax* 2013;68:ii1-ii30.
10. Steiner M, Holzhauer-Barrie J, Lowe D, et al. Pulmonary Rehabilitation: Time to breathe better. National Chronic Obstructive Pulmonary Disease (COPD) Audit Programme: Resources and organisation of Pulmonary Rehabilitation services in England and Wales 2015. National organisational audit report. London: RCP, November 2015.
11. Barr RG, Celli BR, Martinez FJ et al. Physician and patient perceptions in COPD: the COPD Resource Network Needs Assessment Survey. *The American Journal Of Medicine* 2005;118(12):1415-1415.doi:10.1016/j.amjmed.2005.07.059.
12. Harris D, Hayter M, Allender S. Factors affecting the offer of pulmonary rehabilitation to patients with chronic obstructive pulmonary disease by primary care professionals: a qualitative study. *Prim Health Care Res Dev* 2008;9:280-290.doi:10.1017/S1463423608000832.
13. Yawn BP, Wollan PC. Knowledge and attitudes of family physicians coming to COPD continuing medical education. *Int J Chron Obstruct Pulmon Dis* 2008;3(2):311-317.doi:10.2147/COPD.S2486.
14. Johnston CL, Maxwell LJ, Maguire GP et al. How prepared are rural and remote health care practitioners to provide evidence-based management for people with chronic lung disease? *Aust J Rural Health* 2012;20(4):200-207.doi:10.1111/j.1440-1584.2012.01288.x
15. Johnston K, Grimmer-Somers K, Young M et al. Which chronic obstructive pulmonary disease care recommendations have low implementation and why? A pilot study. *BMC Res Notes* 2012;5:652-652.doi:10.1186/1756-0500-5-652
16. Perez X, Wisnivesky JP, Lurslurchachai L et al. Barriers to adherence to COPD guidelines among primary care providers. *Respir Med* 2012;106(3):374-381.doi:/10.1016/j.rmed.2011.09.010
17. Boulet L-P, Bourbeau J, Skomro R et al. Major care gaps in asthma, sleep and chronic obstructive pulmonary disease: a road map for knowledge translation. *Can Respir J* 2013;20(4):265-269.doi:10.1155/2013/496923
18. Desalu OO, Onyedum CC, Adeoti AO et al. Guideline-based COPD management in a resource limited setting - physicians' understanding, adherence and barriers: a cross-sectional survey of internal and family medicine hospital-based physicians in Nigeria. *Prim Care Respir J* 2013;22(1):79-85.doi:10.4104/pcrj.2013.00014
19. Hernandez P, Balter SM, Bourbeau J. et al. Canadian practice assessment in chronic obstructive pulmonary disease: respiratory specialist physician perception versus patient reality. *Can Respir J* 2013;20(2):97-105.doi:10.1155/2013/369019

For peer review only - http://bmjopen.bmj.com/site/about/guidelines.xhtml

20. Johnston K, Young M, Grimmer K et al. Frequency of referral to and attendance at a pulmonary rehabilitation programme amongst patients admitted to a tertiary hospital with chronic obstructive pulmonary disease. *Respirology* 2013;18(7):1089-1094.
21. van der Wees PJ, Zagers KAM, de Die SE et al. Developing a questionnaire to identify perceived barriers for implementing the Dutch physical therapy COPD clinical practice guideline. *BMC Health Serv Res* 2013;13:159-159.doi:10.1186/1472-6963-13-159
22. Walsh JR, McKeough ZJ, Morris NR et al. Performance-based criteria are used in participant selection for pulmonary rehabilitation programs. *Aust Health Rev* 2013;37(3):331-336.
23. Yohannes, A, Stone R, Lowe D et al. Pulmonary rehabilitation in the United Kingdom. *Chron Respir Dis* 2011;8(3): 193-199.doi:10.1071/AH12192
24. Jones RCM, Dickson-Spillman M, Mather MJC et al. Accuracy of diagnostic registers and management of chronic obstructive pulmonary disease: the Devon primary care audit. *Respir Res* 2008;9:62-62.doi:10.1186/1465-9921-9-62
25. Keating A, Lee A, Holland AE. What prevents people with chronic obstructive pulmonary disease from attending pulmonary rehabilitation? A systematic review. *Chron Respir Dis* 2011;8(2):89-99.doi:10.1177/1479972310393756
26. Thorpe O, Johnston K, Kumar S. Barriers and Enablers to Physical Activity Participation in Patients With COPD. A systematic review. *J Cardiopulm Rehab Prev* 2012;32:359-369.
27. Mathar H, Fastholme P, Hansen IR et al. Why Do Patients with COPD Decline Rehabilitation. *Scand J Caring Sci* 2015 DOI:10.1111/scs.12268.doi:10.1111/scs.12268
28. Angus RM, Thompson EB, Davies L et al. Feasibility and impact of a computer-guided consultation on guideline-based management of COPD in general practice. *Prim Care Respir J* 2012;21(4):425-430.doi:10.4104/pcrj.2012.00088
29. Foster F, Piggott R, Riley L, et al. Working with primary care clinicians and patients to introduce strategies for increasing referrals for pulmonary rehabilitation. *Prim Health Care Res Dev* 2016;17(3):226-37.doi:10.1017/S1463423615000286
30. Graves J, Sandrey V, Graves T, et al. Effectiveness of a group opt-in session on uptake and graduation rates for pulmonary rehabilitation. *Chron Respir Dis* 2010;7(3):159-64.doi:10.1177/1479972310379537
31. Hopkinson NS, Englebretsen C, Cooley N et al. Designing and implementing a COPD discharge care bundle. *Thorax*. 2012;67:90-92.doi:10.1136/thoraxjnl-2011-200233
32. Hull S, Mathur R, Lloyd-Owen S, et al. Improving outcomes for people with COPD by developing networks of general practices: evaluation of a quality improvement project in east London. *NPJ Prim Care Respir Med* 2014;24:14082.doi:10.1038/npjpcrm.2014.82
33. Roberts CM, Gungor G, Parker M, et al. Impact of a patient-specific co-designed COPD care scorecard on COPD care quality: a quasi-experimental study. *NPJ Prim Care Respir Med* 2015;25:15017.doi:10.1038/npjpcrm.2015.17
34. Lange P, Rasmussen FV, Borgeskov H et al. The quality of COPD care in general practice in Denmark: the KVASIMODO study. *Prim Care Resp J* 2007;16(3):174-181.doi:10.3132/pcrj.2007.00030
35. Lange P, Andersen KK, Munch E et al. Quality of COPD care in hospital outpatient clinics in Denmark: the KOLIBRI study. *Resp Med* 2009;103:1657-1662.doi:10.1016/j.rmed.2009.05.010
36. Tottenborg SS, Thomsen RW, Nielsen H, et al. Improving quality of care among COPD outpatients in Denmark 2008-2011. *Clin Resp J* 2013;7:319-327.doi:10.1111/crj.12009
37. Ulrik CS, Hansen EF, Jensen MS et al. Management of COPD in general practice in Denmark – participating in an educational program substantially improves adherence to guidelines. *Int J COPD* 2010;5:73-79.
38. Harris M, Smith BJ, Veale AJ, et al. Providing reviews of evidence to COPD patients: controlled prospective 12-month trial. *Chron Respir Dis* 2009;6(3):165-73.doi:10.1177/1479972309106577
39. Zwar NA, Hermiz O, Comino E et al. Care of patients with a diagnosis of chronic obstructive pulmonary disease: a cluster randomised controlled trial. *Med J Aust* 2012Oct 1;197(7):394-8.doi:10.5694/mja12.10813
40. Deprez R, Kinner A, Millard P, et al. Improving Quality of Care for Patients with Chronic Obstructive Pulmonary Disease. *Popul Health Manage* 2009;12(4):209-215.doi:10.1089/pop.2008.0043

For peer review only - http://bmjopen.bmj.com/site/about/guidelines.xhtml

41. Jones AW, Taylor A, Gowler H, et al. Systematic review of interventions to improve patient uptake and completion of pulmonary rehabilitation in COPD. *ERJ Open Res* 2017;3:00089-2016.doi:10.1183/23120541.00089-2016
42. May C, Finch T. Implementing, Embedding, and Integrating Practices: An Outline of Normalization Process Theory. *Sociology* 2009; 43(3):535-554.doi:10.1177/0038038509103208
43. May C, Finch T, Ballini L, et al. Evaluating complex interventions and health technologies using normalization process theory: development of a simplified approach and web-enabled toolkit. *BMC Health Serv Res* 2011;11(1):245.doi:10.1186/1472-6963-11-245
44. May CR et al. Rethinking the patient: using Burden of Treatment Theory to understand the changing dynamics of illness. *BMC Health Serv Res* 2014;14:281.doi:10.1186/1472-6963-14-281
45. Demain S, Gonçalves AC, Areia C et al. Living With, Managing and Minimising Treatment Burden in Long Term Conditions: A Systematic Review of Qualitative Research. *PLoS One* 2015;10(5):e0125457.doi:10.1371/journal.pone.0125457.doi:10.1371/journal.pone.0125457
46. Leppin AL, Montori VM, Gionfriddo MR. Minimally Disruptive Medicine: A Pragmatically Comprehensive Model for Delivering Care to Patients with Multiple Chronic Conditions. *Healthcare (Basel)* 2015;3:50-63.doi:10.3390/healthcare3010050
47. NoMAD ref: Finch TL, Girling M, May CR, Mair FS, Murray E, Treweek S, Steen IN, McColl EM, Dickinson C, Rapley T (2015) NoMad: Implementation measure based on Normalisation Process theory [Measurement Instrument]. Retrieved from http://www.normalizationprocess.org/nomad-study/. Accessed June 2018.
48. Online Surveys. https://www.onlinesurveys.ac.uk/ Accessed June 2018.
49. Ritchie J, Lewis J, McNaughton Nicholls C, Ormston R: Qualitative research practice: a guide for social science students and researchers. 2nd ed. London: Sage; 2014
50. Arnold E, Bruton A, Ellis-Hill C. Adherence to pulmonary rehabilitation: A qualitative study. *Respir Med* 2006;100(10):1716-1723.doi:10.1016/j.rmed.2006.02.007
51. Clarkson PJ, Coleman R, Hosking I, Waller SD, 2011. Inclusive Design Toolkit, second ed. www.inclusivedesigntoolkit.com. Accessed May 2018.
52. Delbecq AL, van de Ven AH. A group process model for problem identification and program planning. *J Appl Behav Sci* 1971;7(4):466–91.doi.org/10.1177/002188637100700404
53. Agile manifesto. http://agilemanifesto.org/principles.html Accessed June 2018.
54. SystmeOne. https://www.tpp-uk.com/products/systmone Accessed June 2018.

For peer review only - http://bmjopen.bmj.com/site/about/guidelines.xhtml

Figures

Figure 1: Mapping of research questions and phases

Figure 2: The fundamental questions of design are solved through successive cycles of exploring needs, creating concepts and evaluating options, guided by project management.[51]

For peer review only

For peer review only - http://bmjopen.bmj.com/site/about/guidelines.xhtml

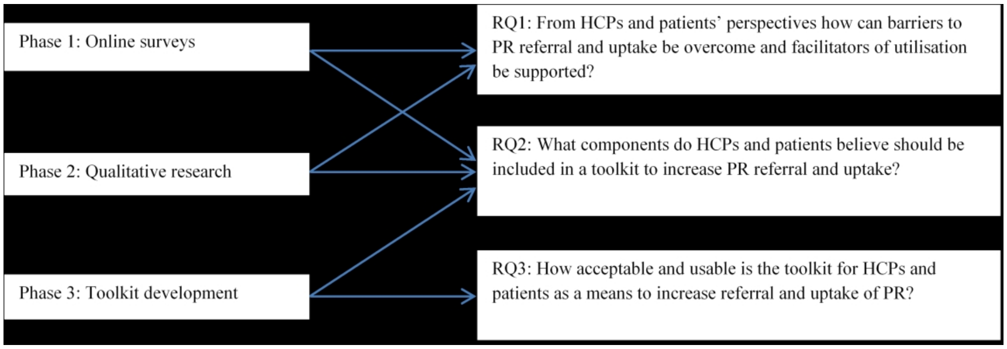

1084x370mm (96 x 96 DPI)

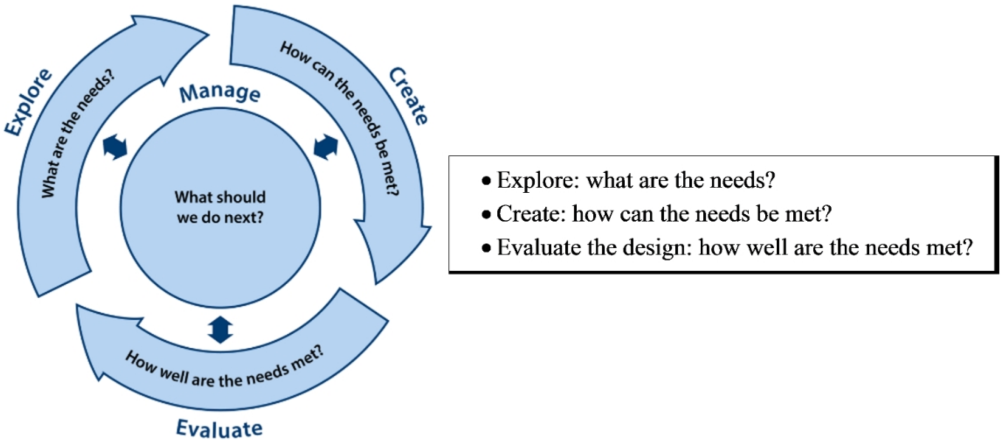

173x75mm (220 x 220 DPI)

Cambridge University Hospitals NHS
NHS Foundation Trust

The REsTORE project: Increasing REferral and uptake TO Pulmonary REhabilitation (general practice survey)

Page 1: Introduction

Thank you for taking part in this survey. The aim is to understand blocks and facilitators to referral and uptake of pulmonary rehabilitation (PR) in primary care and how referral and uptake can be improved.

The survey is part of a research study funded by the National Institute for Health Research (Research for Patient Benefit Programme) that aims to increase the number of people taking up PR. The study also includes interviews and focus groups with patients and clinicians to help us understand what happens during the referral process. We will use what we have learned to develop a toolkit of resources to support referral in primary care, working collaboratively with patients and clinicians.

We request that the survey is completed by a member of clinical staff in your practice who is involved with PR referrals.

The survey should take about 10 to 20 minutes. There are four parts:

- Your role
- Your experience of the PR referral process
- Improving referral and uptake of PR
- Getting in touch

There are no right or wrong answers. All responses and comments are valuable to us. At the end you will be able to leave any additional comments you wish to make.

Your response is valuable to us and so your practice will be paid £10 for the clinical staff

For peer review only - http://bmjopen.bmj.com/site/about/guidelines.xhtml

time needed to complete it. In addition, we invite you to enter a prize draw to win £200 in online shopping vouchers as an incentive to take part. The project is registered on the NIHR CRN Portfolio and completion will count as an accrual for your practice.

On the next two pages you will find participant information about the survey and a consent form for your participation. Please complete the consent form before continuing.

Please complete the survey as soon as possible.

Thank you for your participation.

I confirm that I am a member of clinical staff in my practice who is involved with referral to pulmonary rehabilitation ✱ *Required*

○ Yes

For peer review only - http://bmjopen.bmj.com/site/about/guidelines.xhtml

Page 2: Participant Information

You are invited to take part in an online survey as part of a project to help increase referral and uptake to pulmonary rehabilitation for people with Chronic Obstructive Pulmonary Disease (COPD).

1. What is the purpose of the study?

Our study aims to increase the number of people with COPD who attend pulmonary rehabilitation by developing an online toolkit to help GP practices refer patients successfully. Pulmonary rehabilitation offers supervised exercise and education and helps people to manage their COPD symptoms, improve their quality of life and avoid admission to hospital. It is recommended by NICE. However, the National COPD Audit Programme reported that in 2013/14 an estimated 446,000 COPD patients in England and Wales were eligible for pulmonary rehabilitation but that only 68,000 referrals were made. Of those, only 69% took up the referral offer.

This survey of healthcare professionals (HCPs) in primary care who care for people with COPD is the first step in developing the toolkit. We are also surveying pulmonary rehabilitation providers. Following the survey we will hold interviews and focus groups with patients and HCPs to gather ideas about how to make referral easier and how to help patients make informed decisions about whether to attend pulmonary rehabilitation. We will combine these ideas with evidence from published research and then work with patients and HCPs to assemble the information in an online toolkit. The toolkit could contain, for example, patient-friendly information, electronic reminders on patient records or simplified referral processes. It will be designed to integrate with primary care working practices and to be used, for example, during an annual COPD review. We will test the toolkit in primary care to ensure it is practical for the NHS and has the potential to make a difference.

2. Why have I been chosen to take part in the survey?

We are inviting members of clinical staff in general practices in the East of England who are involved in referral to pulmonary rehabilitation.

3. What will I need to do?

The survey will take about 10 to 20 minutes to complete and is designed to be quick and easy. It will ask about your experiences of referring patients to pulmonary rehabilitation and for your ideas about how to improve referral and uptake. At the end of the survey, we will invite you to provide your contact details if you would like to take part in a subsequent interview or focus group on this topic.

For peer review only - http://bmjopen.bmj.com/site/about/guidelines.xhtml

4. How will the findings be used?

The survey results will be reported in a way that preserves confidentiality and you will not be identified in any way. The results may be published on the Addenbrooke's Hospital intranet, in peer reviewed medical journals, and used for medical presentations, conferences or presentations to patient groups. If you would like to be kept informed of the results please contact the Research Manager, Dr Frances Early, using the details below.

5. What will happen to the information I provide?

All information that is collected is strictly confidential. Only members of the research team will have access to the information. Where data are referred to in published material participants will not be identifiable. Data will be held in secure storage and destroyed after five years. Any information that you give will be used for research purposes only and you may ask to see your personal information at any time.

6. Can I withdraw from the project?

You may withdraw from the study at any time without giving a reason. If you withdraw we will only retain and use any personal information you have provided up to that point if you give us permission to do so.

7. Who has organised the research?

The Lead Investigator for the study is Dr Jonathan Fuld at Cambridge University Hospitals NHS Foundation Trust. Other members of the research team are:

Dr Frances Early (Cambridge University Hospitals NHS Foundation Trust)

Professor Christi Deaton, Dr Ian Wellwood, Dr John Benson, Dr Lois Kim (Cambridge Institute of Public Health)

Ruth Barlow, Lianne Jongepier (East of England Pulmonary Rehabilitation Network)

Professor Patricia Wilson (University of Kent)

Professor Sally Singh (University Hospitals of Leicester NHS Foundation Trust)

The British Lung Foundation

8. How has the study been funded?

The research is funded by the National Institute for Health Research as part of their Research for Patient Benefit programme (award no: PB-PG-1215-20034).

For peer review only - http://bmjopen.bmj.com/site/about/guidelines.xhtml

9. Further information and contact details

If you would like more information please contact Dr Frances Early, Box 146, Cambridge University Hospitals NHS Foundation Trust, Hills Road, Cambridge, CB2 0QQ. Tel 01223 274858. Email: frances.early@addenbrookes.nhs.uk

Ref (WP2 A2 PIS Survey Primary Care v1.1 260617) IRAS ID 209597

For peer review only

Page 3: Consent Form

Please tick each box below to indicate that you understand and agree with the statements

- ☐ I confirm that I have read and understood the information sheet on page 2 of this survey. I have had the opportunity to consider the information, ask questions and have had questions answered satisfactorily
- ☐ I understand my participation is voluntary and I may withdraw at any time without giving any reason
- ☐ I agree that the information I provide can be used solely for the purpose of this reasearch
- ☐ I agree to the use of anonymised data in publications
- ☐ I understand that all personal information will remain confidential
- ☐ I agree to take part in the above study

I confirm my agreement to the above statements ✱ *Required*

For peer review only - http://bmjopen.bmj.com/site/about/guidelines.xhtml

Page 4: Your role

Which best describes your professional job category?

- Practice nurse
- Nurse practitioner
- Advanced nurse practitioner
- Nurse consultant
- Specialist respiratory nurse
- Postgraduate diploma in respiratory care
- GP
- Other

If other, please give details

What responsibilities do you have for the care of people with COPD? Tick as many as apply.

- Annual reviews
- Urgent assessments
- Ongoing management
- Spirometry
- Oxygen therapy
- Admission prevention
- Early discharge facilitation
- Prescribing
- Medication checks
- Opportunistic non-urgent care
- Other

If other, please give details

For peer review only - http://bmjopen.bmj.com/site/about/guidelines.xhtml

How many years' experience do you have of caring for people with respiratory problems?

Please enter a whole number (integer).

How many years have you worked in primary care?

Please enter a whole number (integer).

Please tell us the name and postcode of your general practice so that we can manage the payment to your practice and identify the list size and COPD register details from public sources

For peer review only - http://bmjopen.bmj.com/site/about/guidelines.xhtml

Page 5: The pulmonary rehabilitation (PR) referral process (i)

To which PR services does your general practice refer patients? Tick as many as apply.

- ☐ Norfolk Community (Norfolk Community Health and Care NHS Trust)
- ☐ West Norfolk (BOC Clinical Services)
- ☐ Norfolk & Norwich (Norfolk & Norwich University Hospital NHS Trust)
- ☐ Suffolk (Suffolk Community Healthcare)
- ☐ Great Yarmouth & Waveney: BEET (James Paget University Hospitals NHS FT)
- ☐ West Essex (Essex Partnership University NHS FT)
- ☐ South West Essex (NE London NHS FT)
- ☐ Mid Essex (Provide)
- ☐ North East Essex (Anglian Community Enterprise)
- ☐ Southend (Southend University Hospital NHS FT)
- ☐ Colchester Hospital (Colchester Hospital University NHS FT)
- ☐ Huntingdon & Brookfield (Cambridgeshire & Peterborough NHS FT)
- ☐ Cambridgeshire & Peterborough (Provide)
- ☐ Peterborough and Stamford (Peterborough and Stamford Hospitals NHS FT)
- ☐ Papworth Hospital (Papworth Hospital NHS FT)
- ☐ Luton & Dunstable (Luton & Dunstable University Hospital NHS FT)
- ☐ Luton Community (Cambridgeshire Community NHS FT)
- ☐ Bedford Hospital (Bedford Hospital NHS Trust)
- ☐ East & North Herts (Hertfordshire Community NHS Trust)
- ☐ West Herts (Central London Community Healthcare NHS Trust)
- ☐ Other

If other, please give details

For peer review only - http://bmjopen.bmj.com/site/about/guidelines.xhtml

I feel sure that I understand the eligibility criteria for PR contained in the BTS Guideline on Pulmonary Rehabilitation in Adults

Not at all	☐	☐	☐	☐	☐	☐	☐	☐	☐	☐	☐	Completely

I feel adequately prepared to refer patients to PR

Not at all	☐	☐	☐	☐	☐	☐	☐	☐	☐	☐	☐	Completely

Referring patients to PR is currently a normal part of my work

Not at all	☐	☐	☐	☐	☐	☐	☐	☐	☐	☐	☐	Completely

I find the process of referring patients to PR is easy

Not at all easy	☐	☐	☐	☐	☐	☐	☐	☐	☐	☐	☐	Very easy

What resources (e.g. tools, systems or processes) are available in your general practice to support clinicians in making PR referrals? Tick all that apply.

- ☐ Prompts in clinical system
- ☐ Information about PR for clinicians
- ☐ Clear referral criteria

For peer review only - http://bmjopen.bmj.com/site/about/guidelines.xhtml

☐ Electronic referral ☐ Skills training for communicating effectively with patients ☐ Other

If other, please give details

What resources (e.g. tools, systems or processes) are available in your general practice to support patients in deciding whether to take up a referral? Tick all that apply.

☐ Information about PR for patients ☐ Shared decision-making tools ☐ Other

If other, please give details

For peer review only - http://bmjopen.bmj.com/site/about/guidelines.xhtml

Page 6: The pulmonary rehabilitation (PR) referral process (ii)

What factors influence your decision to refer a patient to PR?

	No influence	Some influence	Strong influence
Increasing shortness of breath	☐	☐	☐
MRC Dyspnoea Scale ≥3	☐	☐	☐
Mobility is affected by breathlessness	☐	☐	☐
Decreasing activity levels	☐	☐	☐
Frequent exacerbations	☐	☐	☐
Recent hospital discharge after exacerbation	☐	☐	☐
Patient is deconditioned	☐	☐	☐
Low exercise tolerance	☐	☐	☐
Patient anxiety	☐	☐	☐
Poor self-management	☐	☐	☐
Easy to use referral process	☐	☐	☐
Other	☐	☐	☐

If other, please give details

What factors might influence your decision *not* to refer an eligible patient to PR?

	No influence	Some influence	A strong influence
I don't have enough information about PR	☐	☐	☐

For peer review only - http://bmjopen.bmj.com/site/about/guidelines.xhtml

I'm uncertain that PR is worthwhile	☐	☐	☐
Patient has doubts that PR is worthwhile	☐	☐	☐
Patient refuses referral	☐	☐	☐
Patient co-morbidities	☐	☐	☐
Poor mobility	☐	☐	☐
Patient does not fit acceptance criteria for a programme	☐	☐	☐
Patient has attended PR previously	☐	☐	☐
Transportation problems	☐	☐	☐
Distance of class from patient's home	☐	☐	☐
Lack of time to make referral	☐	☐	☐
Timing of classes not convenient for patient	☐	☐	☐
I'm unaware of how to refer	☐	☐	☐
Difficult referral process	☐	☐	☐
Other	☐	☐	☐

If other, please give details

For peer review only - http://bmjopen.bmj.com/site/about/guidelines.xhtml

Page 7: The pulmonary rehabilitation (PR) referral process (iii)

For each statement please select the answer that best suits your experience

	Strongly disagree	Disagree	Not sure	Agree	Strongly agree
Staff in my general practice involved in the care of people with respiratory problems have a shared understanding of the purpose of PR	☐	☐	☐	☐	☐
I can see the potential value of referring patients to PR	☐	☐	☐	☐	☐
In my general practice there are key people who drive PR referral forwards and get others involved	☐	☐	☐	☐	☐
I believe that referring patients to PR is a legitimate part of my role	☐	☐	☐	☐	☐
I'm open to working with colleagues in new ways to enable PR referral to happen	☐	☐	☐	☐	☐

For peer review only - http://bmjopen.bmj.com/site/about/guidelines.xhtml

I can easily integrate PR referral into my work	☐	☐	☐	☐	☐
I have confidence in the ability of other colleagues who care for people with respiratory problems to refer patients to PR	☐	☐	☐	☐	☐
In my general practice PR referral is assigned to those with appropriate skills	☐	☐	☐	☐	☐
Sufficient training is provided in my practice so that staff know who to refer to PR	☐	☐	☐	☐	☐
Sufficient training is provided in my practice so that staff know how to refer to PR	☐	☐	☐	☐	☐
Sufficient resources are available to support PR referral in my practice	☐	☐	☐	☐	☐
I am aware of reports in my practice about the outcomes for patients following a PR referral	☐	☐	☐	☐	☐

For peer review only - http://bmjopen.bmj.com/site/about/guidelines.xhtml

The staff in my practice agree that referring patients to PR is worthwhile	☐	☐	☐	☐	☐
I am able to overcome any difficulties I have with regard to referring patients to PR	☐	☐	☐	☐	☐

For peer review only

Page 8: The pulmonary rehabilitation (PR) referral process (iv)

In your experience what are the main reasons that patients take up a referral to PR?

In your experience what are the main reasons why patients decline an offer of PR and do not wish to be referred?

In your experience what are the main reasons that patients referred to PR do not attend their assessment?

For peer review only - http://bmjopen.bmj.com/site/about/guidelines.xhtml

Page 9: Improving referral and uptake of pulmonary rehabilitation (PR)

What would make it easier for you to refer patients to PR?

What could be done to encourage more patients to take up a referral to PR?

Do you receive adequate communication from providers of PR about their PR programmes?

- Yes
- No

Please add comments to clarify if necessary

What do you think is important for effective communication between PR providers

For peer review only - http://bmjopen.bmj.com/site/about/guidelines.xhtml

and primary care?

Which communication methods are effective for you when communicating with PR providers? Select all that apply.

- ☐ Email
- ☐ Letter
- ☐ Telephone
- ☐ Other

If other, please give details

This research project aims to design and build an online toolkit of resources for use in primary care to support referral to PR. What do you think such a toolkit would need to contain or do in order to be successfully adopted by clinicians in primary care?

For peer review only - http://bmjopen.bmj.com/site/about/guidelines.xhtml

Page 10: Other comments

Please leave any other comments you would like to add

For peer review only

For peer review only - http://bmjopen.bmj.com/site/about/guidelines.xhtml

Page 11: Getting in touch

Do you have any resources that support PR referral or uptake that you would be willing to share with the research team?

- ◯ Yes
- ◯ No

Would you like to hear about other ways to get involved in this research study, e.g. developing the toolkit or testing it in practice?

- ◯ Yes
- ◯ No

Thank you for completing this survey. All responses are confidential and results will be reported anonymously. Would you like us to share the results with you?

- ◯ Yes
- ◯ No

Would you like to be entered for the prize draw to win £200 of online shopping vouchers?

- ☐ Yes
- ☐ No

If you answered 'yes' to any of the above questions please leave your contact details here or, if you prefer to keep your responses anonymous, please email the research team directly: frances.early@addenbrookes.nhs.uk

For peer review only - http://bmjopen.bmj.com/site/about/guidelines.xhtml

For peer review only

For peer review only - http://bmjopen.bmj.com/site/about/guidelines.xhtml

Page 12: The survey is now complete. Thank you for your time.

Key for selection options

3 - I confirm my agreement to the above statements

Yes
No

For peer review only

For peer review only - http://bmjopen.bmj.com/site/about/guidelines.xhtml

Cambridge University Hospitals NHS
NHS Foundation Trust

The REsTORE project: Increasing REferral and uptake TO pulmonary REhabilitation (PR provider survey)

Page 1: Introduction

Thank you for taking part in this survey of pulmonary rehabilitation (PR) providers. The aims are to understand the PR referral process, the experiences of PR providers, barriers and facilitators to referral and uptake of PR from primary care and how patients can be encouraged to take up a referral.

The survey is part of a research study funded by the National Institute for Health Research (Research for Patient Benefit Programme) that aims to increase the number of people taking up PR. The study also includes a survey of general practices, and interviews and focus groups with patients and clinicians to help us understand what happens during the referral process. We will use what we have learned to develop a set of resources (a 'toolkit') to support referral in primary care, working collaboratively with patients and clinicians.

We request that the survey is completed by a member of clinical staff in your organisation who is most involved with receiving referrals and assessing respiratory patients.

Your responses are valuable to us and so your service will be paid £10 for the clinical staff time to complete it. In addition, we invite you to enter a prize draw to win £200 in online shopping vouchers as an incentive to take part. The project is registered on the NIHR CRN Portfolio and completion will count as an accrual for your organisation if you are within the NHS.

The survey should take about 10 to 20 minutes. Items include:

- Participant information and consent
- Your role
- Your pulmonary rehabilitation service
- Your experience of the PR referral process
- Improving referral and uptake of PR
- Getting in touch

There are no right or wrong answers. All responses and comments are helpful. At the end you will be able to leave any additional comments you wish to make.

On the next two pages you will find participant information about the survey and a consent form for your participation. Please complete the consent form before continuing.

Thank you for your participation.

I confirm that I am a member of clinical staff involved in receiving referrals and assessing patients for pulmonary rehabilitation

○ Yes

For peer review only - http://bmjopen.bmj.com/site/about/guidelines.xhtml

Page 2: Participant Information

You are invited to take part in an online survey as part of a project to help increase referral and uptake to Pulmonary Rehabilitation (PR) for people with Chronic Obstructive Pulmonary Disease (COPD).

1. What is the purpose of the study?

Our study aims to increase the number of people with COPD who attend PR by developing an online toolkit to help GP practices refer patients successfully. The National COPD Audit Programme reported that in 2013/14 an estimated 446,000 COPD patients in England and Wales were eligible for PR but that only 68,000 referrals were made. Of those, only 69% took up the referral offer.

This survey of PR providers is the first step in developing the toolkit. We are also surveying healthcare professionals (HCPs) in primary care who care for people with COPD. Following the survey we will hold interviews and focus groups with patients and HCPs to gather ideas about how to make referral easier and how to help patients make informed decisions about whether to attend PR. We will combine these ideas with evidence from published research and then work with patients and HCPs to assemble the information in an online toolkit. The toolkit could contain, for example, patient-friendly information, electronic reminders on patient records or simplified referral processes. It will be designed to integrate with primary care working practices and to be used, for example, during an annual COPD review. We will test the toolkit in primary care to ensure it is practical for the NHS and has the potential to make a difference.

2. Why have I been chosen?

We are inviting PR providers in the East of England to take part in the survey.

3. What will I need to do?

The survey will take about 10 to 20 minutes to complete and is designed to be quick and easy. It will ask about your experiences of referring patients to PR and for your ideas about how to improve referral and uptake. At the end of the survey, we will invite you to provide your contact details if you would like to take part in a subsequent interview or focus group on this topic.

4. How will the findings be used?

The survey results will be reported in a way that preserves confidentiality and you will not be identified in any way. The results may be published on the Addenbrooke's Hospital intranet, in peer reviewed medical journals, and used for medical presentations, conferences or presentations to patient groups. If you would like to be kept informed of the results please contact the Research Manager, Dr Frances Early, using the details below.

5. What will happen to the information I provide?

All information that is collected is strictly confidential. Only members of the research team will have access to the information. Where data are referred to in published material participants will not be identifiable. Data will be held in secure storage and destroyed after five years. Any information that you give will be used for research purposes only and you may ask to see your personal information at any time.

6. Can I withdraw from the project?

You may withdraw from the study at any time without giving a reason. If you withdraw we will only retain and use any personal information you have provided up to that point if you give us permission to do so.

7. Who has organised the research?

The Lead Investigator for the study is Dr Jonathan Fuld at Cambridge University Hospitals NHS Foundation Trust. Other members of the research team are:

Dr Frances Early (Cambridge University Hospitals NHS Foundation Trust)

Professor Christi Deaton, Dr Ian Wellwood, Dr John Benson, Dr Lois Kim (Cambridge Institute of Public Health)

Ruth Barlow, Lianne Jongepier (East of England Pulmonary Rehabilitation Network)

Professor Patricia Wilson (University of Kent)

Professor Sally Singh (University Hospitals of Leicester NHS Foundation Trust)

The British Lung Foundation

8. How has the study been funded?

The research is funded by the National Institute for Health Research as part of their Research for Patient Benefit programme (award no: PB-PG-1215-20034).

9. Further information and contact details

If you would like more information please contact Dr Frances Early, Box 146, Cambridge University Hospitals NHS Foundation Trust, Hills Road, Cambridge, CB2 0QQ. Tel 01223 274858. Email@ frances.early@addenbrookes.nhs.uk

(Participant information sheet WP2 A4 PIS Survey Pul Rehab v1.1 090717) IRAS ID 209597

For peer review only - http://bmjopen.bmj.com/site/about/guidelines.xhtml

Page 3: Participant Consent

Please tick each box below to indicate that you understand and agree with the statements

- ☐ I confirm that I have read and understood the above information sheet (WP2 A4 PIS Survey Pul Rehab v1.1 090717). I have had the opportunity to consider the information, ask questions and have had questions answered satisfactorily.
- ☐ I understand my participation is voluntary and I may withdraw at any time without giving any reason
- ☐ I agree that the information I provide can be used solely for the purpose of this research
- ☐ I agree to the use of the anonymised data in publications
- ☐ I understand that all personal information will remain confidential
- ☐ I agree to take part in the above study

I confirm my agreement to the above statements ✱ *Required*

Page 4: Your organisation

Please tell us the name and contact details of your PR provider organisation so that we can manage the payment for completion of this survey

For peer review only - http://bmjopen.bmj.com/site/about/guidelines.xhtml

Page 5: Please tell us about your role so that we can understand who is involved in delivering pulmonary rehabilitation

What best describes your professional job category?

- ○ Physiotherapist
- ○ Nurse
- ○ Health support worker
- ○ Health care assistant
- ○ Occupational therapist
- ○ Dietician
- ○ Fitness instructor/trainer
- ○ Pharmacist
- ○ Other

If other, please give details

How many years have you been involved in the care of people with respiratory problems?

Please enter a whole number (integer).

What is your role in pulmonary rehabilitation? Please select all that apply.

- ☐ I manage the PR service
- ☐ I manage the PR team
- ☐ I run a PR class
- ☐ I assist with a PR class
- ☐ I work in the admin team and screen the PR referrals
- ☐ Other

If other, please give details

How many years have you worked in your pulmonary rehabilitation role?

Please enter a whole number (integer).

What is the WTE (Whole Time Equivalent) of your post?

What band or grade is your post?

For peer review only - http://bmjopen.bmj.com/site/about/guidelines.xhtml

For peer review only - http://bmjopen.bmj.com/site/about/guidelines.xhtml

Page 6: Please tell us about your pulmonary rehabilitation service

What type of organisation provides your PR service?

- ☐ NHS Trust or health board
- ☐ Community Interest Company (CIC)
- ☐ Private healthcare provider
- ☐ Charity
- ☐ Research
- ☐ Other

If other, please give details

Is your service open to referrals all year round?

- ○ Yes
- ○ No

Do you accept referrals from

- ○ Primary care
- ○ Secondary care
- ○ Primary and secondary care
- ○ Self-referral
- ○ Other

If other, please give details

What type of PR programmes do you offer?

- ☐ Rolling
- ☐ Cohort
- ☐ Rolling and cohort
- ☐ Other

If rolling, how many rolling programmes per year?

If cohort, how many cohorts per year?

If other, please give details

Do you offer group-based and/or one-to-one programmes?

- ☐ Group-based
- ☐ One-to-one
- ☐ Group-based and one-to-one
- ☐ Other

If other, please give details

In which settings does your service hold classes? Tick all that apply.

- ☐ Community settings
- ☐ Hospital settings
- ☐ Primary care settings
- ☐ Home settings
- ☐ Other

If other, please give details

At how many sites does your service offer PR?

Please enter a whole number (integer).

Do you offer initial assessment before enrolment on to PR?

- ○ Yes
- ○ No

How many exercise sessions do you offer for each course of PR (excluding assessment visits)?

Please enter a whole number (integer).

Page 7: Please tell us about the funding and capacity of your pulmonary rehabilitation service

How is your PR service funded?

- ☐ CCG commissioned
- ☐ Hospital funded
- ☐ Post-discharge rehabilitation tarrif
- ☐ Other

If other, please give details

Does your PR service have a fixed term of funding?

- ○ Yes
- ○ No
- ○ Don't know

If yes, how many years future funding does the service have? Please leave blank if you are not sure.

How many available places did your service have last year?

Please enter a number.

How many places did you fill in the last year (2016/17)?

Please enter a number.

What is the maximum number of places (across all sites) that your PR service can offer in the current financial year (2017-18) based on your staffing levels?

Please tick here if you are not sure

- ○ I'm not sure what the number of places is
- ○ I have given an estimate below

Estimated number of places

Please add any comments for clarification regarding funding or capacity

For peer review only - http://bmjopen.bmj.com/site/about/guidelines.xhtml

Page 8: Your experience of the pulmonary rehabilitation referral process (part i)

I understand the eligibility criteria for PR contained in the BTS Guideline on Pulmonary Rehabilitation in Adults

Please don't select more than 1 answer(s) per row.

	0	1	2	3	4	5	6	7	8	9	10	
Not at all	☐	☐	☐	☐	☐	☐	☐	☐	☐	☐	☐	Completely

The process of accepting patients onto a PR programme following referral is:

Please don't select more than 1 answer(s) per row.

	0	1	2	3	4	5	6	7	8	9	10	
Very difficult	☐	☐	☐	☐	☐	☐	☐	☐	☐	☐	☐	Very easy

I feel adequately prepared to accept patient referrals to PR

Please don't select more than 1 answer(s) per row.

	0	1	2	3	4	5	6	7	8	9	10	
Not at all	☐	☐	☐	☐	☐	☐	☐	☐	☐	☐	☐	Completely

Page 9: Your experience of the PR referral process (part ii)

Thinking of your work at the moment how strongly do you agree with the following statements?

Please don't select more than 1 answer(s) per row.

	Strongly disagree	Disagree	Not sure	Agree	Strongly agree
Staff in my organisation have a shared understanding of the purpose of PR	☐	☐	☐	☐	☐
There are key people in my organisation who promote PR	☐	☐	☐	☐	☐
I am open to working with colleagues in new ways to enable PR referral to happen	☐	☐	☐	☐	☐
I have confidence in clinicians' ability to refer patients to PR	☐	☐	☐	☐	☐
Work involved in processing PR referrals is assigned to people in my organisation who have the appropriate skills	☐	☐	☐	☐	☐
I feel I have had sufficient training to process referrals to PR	☐	☐	☐	☐	☐
I feel that sufficient resources are available in my organisation to support the processing of PR referrals	☐	☐	☐	☐	☐
My organisation provides adequate support for the PR referral process to enable it to be effective	☐	☐	☐	☐	☐
I am aware of audits or feedback reports within my organisation about the effectiveness of the PR referral process	☐	☐	☐	☐	☐
We adapt PR referral processes in light of feedback we receive	☐	☐	☐	☐	☐

For peer review only - http://bmjopen.bmj.com/site/about/guidelines.xhtml

Page 10: Your experience of the PR referral process (part iii)

What do you think are the main reasons that patients take up a referral to PR?

What do you think are the main reasons that patients who have been referred do not attend an assessment appointment?

Page 11: Communication and processes in pulmonary rehabilitation referral

Do you receive adequate communication from referrers about patients who have been referred to your service?

- Yes
- No

Please add comments to clarify if necessary

Which communication methods are effective for you when communicating with referrers? Select all that apply.

- Email
- Letter
- Telephone
- Other

If other, please give details

What do you think is important for effective communication between primary care and PR providers?

Do you send written reports to referrers about patients who have been referred to your service?

- Yes
- No

Please add comments to clarify if necessary

What tools, systems or processes are available in your organisation to support patients in their decision-making about whether to attend a PR assessment?

- Information about PR for patients
- Shared decision-making tools
- Other

If other please give details

For peer review only - http://bmjopen.bmj.com/site/about/guidelines.xhtml

For peer review only - http://bmjopen.bmj.com/site/about/guidelines.xhtml

Page 12: Improving referral and uptake to pulmonary rehabilitation

What do you think would make it easier for clinicians to refer patients to PR?

What do you think could be done to encourage patients to attend an assessment appointment following referral?

Page 13: Developing the pulmonary rehabilitation toolkit

Our research study aims to design and build a collection of resources (a 'toolkit') for use in primary care in order to support successful referral to PR. What do you think are the most important things that such a toolkit should contain or do in order for it to be acceptable and useful?

For peer review only - http://bmjopen.bmj.com/site/about/guidelines.xhtml

Page 14: Getting in touch

We are keen to gather examples of existing resources that may be useful to share through the toolkit. Do you have any resources that support the processing of PR referrals which you would be willing to share with the research team?

- Yes
- No

Would you like to enter the prize draw to win £200 of online shopping vouchers?

- Yes
- No

Thank you for completing this survey. All responses are confidential and results will be reported anonymously. Would you like us to share the results with you?

- Yes
- No

If you answered 'yes' to any of the above questions please leave your contact details here or, if you prefer to keep your responses anonymous, please email the research team directly: frances.early@addenbrookes.nhs.uk

Any other comments?

Page 15: The survey is now complete. Thank you for your time.

Key for selection options

3 - I confirm my agreement to the above statements

Yes
No

For peer review only - http://bmjopen.bmj.com/site/about/guidelines.xhtml

Supplement 3

REsTORE study focus group / interview schedule for HCPs in primary care

Perception of PR

1. How much do you know about PR?
2. How important is PR as treatment for COPD?
   a) Do you think PR is useful?
3. Do you think there are any differences in how you view PR and how other doctors, nurses and physiotherapists view it?
4. Is PR different from other parts of the COPD pathway? If yes – how?
   a) For example -Do you perceive it as being integral to the pathway?
   b) Are some components of the pathway are more important / effective / useful / acceptable?

Referral in practice

5. To what extent is PR referral part of your role?
6. What criteria do you use when referring people to COPD?
   a) How do you judge if it is worthwhile referring someone?
7. Can you describe what you do to refer someone to PR?
   a) Would you say there are differences in referral practice?
   b) Does everyone do it the same way?
   c) What tools/processes etc. are in place in your practice to ensure that PR referrals are made efficiently?
8. How would you describe the communication between you and the PR providers?
   a) For example, what types do you use and what is most effective - written, electronic/ telephone/person
9. How confident are you that referrals will be efficiently acted upon by other people in the pathway?
   a) Are there any efficiency standards for this?
10. Why do you think patients do or do not take up an offer?
11. Why do you think some HCPs might refer more people to PR than others?
    a) For example, differences between professionals or differences between practices i.e. variation within or between practices

Improving referral

12. What would make you more likely to refer to PR?
13. What would make it easier for you to refer to PR?
14. What would make it easier for patients to take up a referral?

Organisational support

15. Who drives PR referral forwards in your practice?
    a) What would make it easier to sustain levels of referrals?

Toolkit

16. Do you think a PR tool kit would be useful?
17. How would you judge whether a PR toolkit was worthwhile?

30204911_File000014_703069988.doc

For peer review only

Supplement 4
REsTORE study focus group schedule for patients who have accepted a referral to PR

Introductions and welcome

1. Do you know what Chronic Obstructive Pulmonary Disease (COPD) is?

a) Clarify understanding if patient is not sure.
b) Offer COPD information leaflet.

2. What is it like to live with COPD?

a) Can you tell me what you know about COPD and treatments?
b) What do you have to do to manage your COPD, e.g. appointments, treatments, self-care…?
c) How do you manage these things? Who or what helps you?
d) Thinking about the demands of managing your COPD, what effect has this had on you and your life?

3. Can you describe how you were referred to PR?

a) Who referred you?
b) What did they tell you about the referral process or about PR, e.g. what PR is, why it might be a good idea for you? (PPI feedback indicated that this is a key question)
c) How much did you know about PR before you attended?

4. Why did you choose to attend PR?

a) Thinking about other people who could benefit from PR, what do you think they might need in order for them to be able to attend?
b) Thinking about other people could benefit from PR, what do you think would make them want to attend?

5. What things made it difficult for you to attend PR?

a) Challenges could be in their own life or in the healthcare system
b) What do you have to do to be able to attend, e.g. change to routines, being open to trying something new…?
c) How do you manage these things?
d) Who or what helps you to attend PR?
e) What would make attending PR easier?

6. How has PR helped you compared to other treatments you are receiving for COPD?

a) How has attending PR affected you or your life (positive or negative)?

For peer review only

For peer review only - http://bmjopen.bmj.com/site/about/guidelines.xhtml

[Type text]

Supplement 5
REsTORE study focus group schedule for patients who have declined a referral to PR

1. **Do you know what Chronic Obstructive Pulmonary Disease (COPD) is?**
   a) Clarify understanding if patient is not sure.
   b) Offer COPD information leaflet.

2. **What is it like to live with COPD?**
   a) Can you tell me what you know about COPD and treatments?
   b) What do you have to do to manage your COPD, e.g. appointments, treatments, self-care…?
   c) How do you manage these things? Who or what helps you?
   d) Thinking about the demands of managing your COPD, what effect has this had on you and your life?

3. **Do you know what pulmonary rehabilitation (PR) is?**
   a) Clarify understanding if patient is not sure.
   b) Offer PR information leaflet.

4. **Can you describe how you were referred to PR?**
   a) Who referred you?
   b) What did they tell you about the referral process or about PR, e.g. what PR is, why it might be a good idea for you? (PPI feedback indicated that this is a key question)
   c) How much were you told about what happens in the PR classes?
   d) How much were you told about what you would need to do if you attended PR?

5. **Can you tell me the reasons why chose not to attend PR?**
   a) How did you decide whether or not it would help you?

6. **Were there any difficulties that put you off attending or made it impossible for you to attend PR?**
   a) Challenges could be in their own life or in the healthcare system
   b) What would you have had to do in order to attend, e.g. changed a routine, tried something unfamiliar …?
   c) What would make attending PR easier / what would help?
   d) What would make attending PR more attractive?
   e) What kind of support might you need to attend PR?
   f) How would PR need to be different for you to want to attend?

7. **What do you know about how helpful PR could be compared to other treatments for COPD?**
   a) How important do you think PR is as a treatment?

Or, if interviewee believes that PR will be of no help to them:

8. **What other treatments are you on for COPD?**
   a) What do you think makes these more helpful in managing your COPD compared to PR?

For peer review only - http://bmjopen.bmj.com/site/about/guidelines.xhtml

Supplement 6

REsTORE study focus group schedule for patients who have not been referred to PR

1. **Do you know what Chronic Obstructive Pulmonary Disease (COPD) is?**
   a) Clarify understanding if patient is not sure.
   b) Offer COPD information leaflet.

2. **What is it like to live with COPD?**
   a) Can you tell me what you know about COPD and treatments?
   b) What do you have to do to manage your COPD, e.g. appointments, treatments, self-care…?
   c) How do you manage this, who or what helps you?
   d) Thinking about the demands of managing your COPD, what effect has this had on you and your life?

3. **Do you know what pulmonary rehabilitation (PR) is?**
   a) Clarify understanding if patient is not sure.
   b) Offer PR information leaflet.

4. **Have you had any conversations with your nurse or GP about PR?**
   a) Who was the conversation with?
   b) What was said?
   c) What did they tell you about the referral process or about PR, e.g. what PR is, why it might be a good idea for you? (PPI feedback indicated that this is a key question)
   d) What do you know about what PR involves?
   e) If patient does not know about PR, offer information about PR (British Lung Foundation leaflets)

5. **Would you like to be referred to PR?**

   If YES or NOT SURE go to question 6. If NO go to question 8.

6. **If yes or not sure to question 5: What do you know about how helpful PR could be compared to other treatments for COPD?**
   a) How important do you think PR is as a treatment for COPD? [Note that this question may depend on why there were not referred]
   b) What might you want from a programme like PR (i.e. an exercise and education programme) that would help you to manage your COPD?

7. **What sort of problems or obstacles, if any, would you need to overcome to be able to attend a PR course?**
   a) Challenges could be in their own life or in the healthcare system
   b) What do you think you would have to do in order to be able to attend, e.g. change routines, try something unfamiliar…?
   c) What would make attending PR easy / what would help?
   d) What would make attending PR attractive?
   e) What kind of support might you need to attend PR?

   END OF QUESTIONS

8. **If no to question 5: Can you tell me the reasons why you would not want to be referred?**
   a) Can you tell me about whether you think it would or would not help you?

9. **Are there any difficulties that would put you off attending or make it impossible for you to attend PR?**

For peer review only - http://bmjopen.bmj.com/site/about/guidelines.xhtml

a) Challenges could be in their own life or in the healthcare system
b) What do you think you would have to do in order to be able to attend, e.g. change a routine, try something unfamiliar…?
c) What would make attending PR easy / what would help?
d) What would make attending PR attractive?
e) What kind of support might you need to attend PR?
f) How would PR need to be different for you to want to be referred?

END OF QUESTIONS

For peer review only

For peer review only - http://bmjopen.bmj.com/site/about/guidelines.xhtml

Supplement 7
REsTORE study focus group schedule for PR providers

Perception of PR

1. What do you think is the value of PR as a treatment for COPD?
   a) Explore any differences in how its value is perceived
   b) Why do you think some patients take up an offer but others don't?

Referral in practice

2. Can you describe what you do to process a PR referral?
   a) What tools/processes etc. are in place in your organisation to ensure that PR referrals are processed efficiently?
   b) What would make it easier for you to efficiently process referrals?
   c) What could be done in your organisation to make efficient processing of PR referral easier to sustain?

3. How effective is communication between referrers and PR providers?

4. In your experience why do you think some HCPs refer more patients to PR than others?

Improving referral

5. What could be done to increase referral to PR?
6. What could be done to increase uptake to PR?

Toolkit (Introduce what a toolkit is)

7. How would you judge whether a PR referral toolkit in primary care was worthwhile?

For peer review only - http://bmjopen.bmj.com/site/about/guidelines.xhtml

Supplement 8

REsTORE study interview schedule for commissioners

Perception of PR

1. How much do you know about PR?
   a) How valuable do you think PR is as a treatment for COPD?

2. Is PR different from other parts of the COPD pathway? If yes, how?

Referral in practice and how you support PR providers in making referrals

3. How confident are you as a commissioner that the PR referral pathway is performing efficiently?
   a) What tools or processes are in place in your organisation to support practices in making sure that PR referrals are made efficiently?

4. To what extent is it part of your role to support PR referral and utilisation in primary care?
   a) How do commissioners support practitioners in their assessment of whether or not to refer people to PR?

5. Why do you think some HCPs might refer more people to PR than others?
6. Why do you think patients do or don't take up an offer of PR?

Improving referral

7. What could commissioners do to make it easier for practices to successfully refer to PR?
8. What would make it easier for patients to take up a referral?

Support within your organisation

9. Who or what drives the requirement for PR utilisation in primary care forwards in your organisation?
10. In your organisation, what would make support for PR utilisation easier to sustain?

Toolkit

11. Do you think a PR referral toolkit would be useful?
12. How would you judge whether a PR referral toolkit was worthwhile?

For peer review only

30204911_File000019_703070272.docx

BMJ Open

Developing an intervention to increase REferral and uptake TO pulmonary REhabilitation in primary care in patients with Chronic Obstructive Pulmonary Disease (the REsTORE study): mixed methods study protocol

Journal:	*BMJ Open*
Manuscript ID	bmjopen-2018-024806.R1
Article Type:	Protocol
Date Submitted by the Author:	29-Aug-2018

Complete List of Authors:	Early, Frances; Cambridge University Hospitals NHS Foundation Trust, Box 146<br>Wilson, Patricia; University of Kent, Kent Academic Primary Care Unit<br>Deaton, Christi; University of Cambridge, Cambridge Institute of Public Health, School of Clinical Medicine<br>Wellwood, Ian; University of Cambridge, Cambridge Institute of Public Health, School of Clinical Medicine<br>Dickerson, Terry; University of Cambridge, Engineering Design Centre<br>Ward, James; University of Cambridge, Engineering<br>Jongepier, Lianne; NHS North East Essex Clinical Commissioning Group<br>Barlow, Ruth; Provide Community Interest Company<br>Singh, Sally; University Hospitals of Leicester NHS Trust, Cardiac/Pulmonary Rehabilitation<br>Benson, John; University of Cambridge, GP and Primary Care Research Unit<br>Brimicombe, James; University of Cambridge, Primary Care Unit, Department of Public Health and Primary Care; University of Cambridge<br>Kim, Lois; University of Cambridge Department of Public Health and Primary Care<br>Haque, Hena; Cambridge University Hospitals NHS Foundation Trust, Centre for Self Management Support<br>Fuld, Jonathan; Cambridge University Hospitals NHS Foundation Trust, Centre for Self Management Support

**Primary Subject Heading**:	Respiratory medicine
Secondary Subject Heading:	Health services research, Qualitative research
Keywords:	Quality in health care < HEALTH SERVICES ADMINISTRATION & MANAGEMENT, PRIMARY CARE, QUALITATIVE RESEARCH, REHABILITATION MEDICINE, RESPIRATORY MEDICINE (see Thoracic Medicine), Chronic airways disease < THORACIC MEDICINE

For peer review only - http://bmjopen.bmj.com/site/about/guidelines.xhtml

Developing an intervention to increase REferral and uptake TO pulmonary REhabilitation in primary care in patients with Chronic Obstructive Pulmonary Disease (the REsTORE study): mixed methods study protocol

Corresponding author:

Dr Frances Early, Centre for Self Management Support, Box 146, Cambridge University Hospitals NHS Foundation Trust, Cambridge Biomedical Campus, Hills Road, Cambridge CB2 0QQ. Email: Frances.early@addenbrookes.nhs.uk. Tel: 01223 274858.

Authors:

Frances Early (corresponding author)

Patricia Wilson, Centre for Health Services Studies, University of Kent, Canterbury CT2 7NF. UK.

Christi Deaton, Department of Public Health and Primary Care, University of Cambridge School of Clinical Medicine, Box 111 Cambridge Biomedical Campus, Cambridge, CB2 0SP. UK

Ian Wellwood, Department of Public Health and Primary Care, University of Cambridge School of Clinical Medicine, Box 111 Cambridge Biomedical Campus, Cambridge, CB2 0SP. UK

Terry Dickerson, Department of Engineering, University of Cambridge, Trumpington Street, Cambridge, CB2 1PZ. UK

James Ward, Department of Engineering, University of Cambridge, Trumpington Street, Cambridge, CB2 1PZ. UK

Lianne Jongepier, NHS North East Essex Clinical Commissioning Group, Aspen House, Stephenson Road, Severalls Business Park, Colchester, CO4 9QR. UK

Ruth Barlow, Provide Community Interest Company, 900 The Crescent, Colchester Business Park, Colchester, Essex, CO4 9YQ. UK

Sally Singh, University Hospitals of Leicester NHS Trust, Cardiac/Pulmonary Rehabilitation Glenfield Hospital, Groby Rd, Leicester, LE3 9QP. UK

John Benson, Department of Public Health and Primary Care, University of Cambridge School of Clinical Medicine, Box 111 Cambridge Biomedical Campus, Cambridge, CB2 0SP. UK

James Brimicombe, Department of Public Health and Primary Care, University of Cambridge School of Clinical Medicine, Box 111 Cambridge Biomedical Campus, Cambridge, CB2 0SP. UK

Lois Kim, Department of Public Health and Primary Care, University of Cambridge School of Clinical Medicine, Box 111 Cambridge Biomedical Campus, Cambridge, CB2 0SP. UK

Hena Haque, Centre for Self Management Support, Box 146, Cambridge University Hospitals NHS Foundation Trust, Cambridge Biomedical Campus, Hills Road, Cambridge CB2 0QQ. UK

Jonathan Fuld, Centre for Self Management Support, Box 146, Cambridge University Hospitals NHS Foundation Trust, Cambridge Biomedical Campus, Hills Road, Cambridge CB2 0QQ. UK

Word count: 4,294

For peer review only - http://bmjopen.bmj.com/site/about/guidelines.xhtml

Keywords

Quality in healthcare, primary care, qualitative research, rehabilitation medicine, respiratory medicine, chronic airways disease

For peer review only

For peer review only - http://bmjopen.bmj.com/site/about/guidelines.xhtml

Abstract

Introduction: Chronic Obstructive Pulmonary Disease (COPD) is a progressive lung disease associated with breathlessness, inability to exercise, frequent infections, hospitalisation and reduced quality of life. Pulmonary rehabilitation (PR), providing supervised exercise and education, is an effective and cost-effective treatment for COPD but is significantly underutilised. Interventions to improve referral and uptake have been tested and some positive results reported. However, interventions are diverse and no clear recommendations for practice can be made. This study aims to understand the challenges to referral and uptake in primary care, where most referrals originate, and to develop a flexible toolkit of resources to support referral and uptake to PR in primary care in the UK.

Methods and analysis: This is a mixed methods study informed by Normalisation Process Theory and Burden of Treatment theory. In the first phase GPs, practice nurses and PR providers will be invited to complete an online survey to inform a broad exploration of the topic areas. In phase 2 interviews and focus groups will be conducted with patients, healthcare professionals (HCPs) in primary care, PR providers and commissioners to gain an in-depth understanding of the issues and needs. Toolkit development in phase 3 will draw together the learning from phases 1 and 2 and employ an iterative development process to build the toolkit jointly with patients and HCPs. It will be tested in primary care for usability and acceptability.

Ethics and dissemination: The study has ethical and Health Research Authority approval (Research Ethics Committee reference number 17/EE/0136). It is registered with the International Standard Registered Clinical/soCial sTudy Number (ISRCTN) registry (trial ID: ISRCTN20669629, assignment date 20th March 2018, trial start date 1st April 2016). Dissemination will be aimed at patients, carers/families, service providers, commissioners and national interest groups. Methods will include conferences, presentations, academic publications and plain English reports and will be supported by the British Lung Foundation.

Strengths and limitations of this study

- The study explicitly uses Normalisation Process Theory to inform data collection methods, data analysis and toolkit development which will maximise potential for the toolkit to be successfully implemented in practice.
- Patients who are unable to communicate in English, Hindi or Urdu will be excluded from the study.
- Patients who have declined PR may be challenging to engage and so we will work closely with primary care to recruit this group.
- An iterative design process enables rapid development of the toolkit in a way that readily allows it to be refined and tailored to the needs of users and the overall system, resulting in a design of higher quality which is more likely to stand the test of time.
- Outputs from survey and qualitative data will support identification of design priorities at the start of development which will provide focus for the iterative process.

For peer review only - http://bmjopen.bmj.com/site/about/guidelines.xhtml

INTRODUCTION

Chronic Obstructive Pulmonary Disease (COPD) is a progressive lung disease associated with breathlessness, inability to exercise, frequent infections and hospitalisation. Approximately 1.2 million people were living with diagnosed COPD in England in 2012[1] and annual direct healthcare costs of COPD in England have been estimated to increase from £1.50 billion in 2011 to £2.32 billion in 2030.[2] Pulmonary rehabilitation (PR), providing supervised exercise and education, is an effective treatment for COPD that leads to clinically significant improvements in exercise capacity, symptoms and health-related quality of life in patients who experience disabling breathlessness.[3] PR also supports self-management skills[4] and results in fewer and shorter hospital admissions[5] and readmissions.[6] It is a cost-effective treatment[7] recommended for patients who are functionally disabled by COPD.[8]

However, despite published guidelines,[8-9] PR is significantly underutilised. In England and Wales in 2013/14 the estimated prevalence of COPD patients eligible for PR was 446,000 but only 68,000 referrals were received by PR programmes. Of those patients referred, 31% did not attend for assessment.[10] There is an urgent need to review PR referral pathways, healthcare professional (HCP) training, information for patients and referrers, and barriers to patient access, particularly in primary care in England and Wales where 51% of referrals originate.[10]

Referral rates are impacted by a range of factors including difficult referral processes, lack of information about PR and unclear roles and responsibilities regarding referral.[11-24] A recent systematic scoping review of barriers and enablers to PR referral identified the most common barriers to be low knowledge of, or disbelief in, the benefits of PR and low knowledge of the referral process; other frequently identified barriers were low knowledge of the eligibility criteria, low awareness of PR and a belief that gaining behaviour change in reluctant patients would be too difficult.[25] The same review reported that the most frequently identified enablers of PR referral were PR training, mentoring or experience in PR; other enablers were PR awareness events, reminders and a streamlined referral process. Uptake by patients is impacted by the quality of the HCP conversation about PR and patients' beliefs about the benefits, as well as timing, location and transport.[26-28]

Interventions to improve referral and uptake have been reported[29-42] and the results of these have been summarised in a systematic review.[43] In this review four out of 10 studies that measured referral reported statistically significant increases in referral: the interventions included a patient-held care quality scorecard [Roberts], clinician education [35,41] and mandatory monitoring of quality indicators in a hospital setting.[37] Two out of four studies measuring uptake reported statistically significant increases in uptake: a patient manual summarising evidence of COPD treatments [39] and individualised care planning supported by nurses and general practitioners (GPs).[40] Most of the studies measured referral or uptake to PR in the context of multifaceted evidence-based management of COPD. Clear recommendations for practice could not be made due to the diverse range of study designs and most study designs carrying a high risk of bias.[43]

This paper presents the protocol for a mixed methods study to support the improvement of referral and uptake to PR from primary care. The study runs from April 2016 to December 2018.

Aims and objectives

For peer review only - http://bmjopen.bmj.com/site/about/guidelines.xhtml

The aim is to understand the needs of HCPs and patients in general practice concerning the challenges to referral and uptake, and then use this understanding to develop a flexible toolkit of resources to support referral and uptake of PR within primary care.

The research questions are:

1. From HCPs and patients' perspectives how can barriers to PR referral and uptake be overcome and facilitators of utilisation be supported?
2. What components do HCPs and patients believe should be included in a toolkit to increase PR referral and uptake?
3. How acceptable and usable is the toolkit for HCPs and patients as a means to increase referral and uptake of PR?

Objectives to address the research questions are:

a) Conduct empirical research to identify factors that HCPs and patients believe will overcome barriers and facilitate referral and uptake to PR in primary care.
b) Synthesise evidence from empirical research and our systematic literature review to develop the toolkit collaboratively with patients and providers.
c) Conduct exploratory efficacy testing of the toolkit in a sample of primary care practices to assess performance and gather data to provide a basis for future economic analysis.

Guiding theory

The research design is informed by Normalisation Process Theory,[44-45] an established theoretical framework for understanding implementation processes through multiple stakeholder perspectives. Normalisation Process Theory will provide the theoretical underpinning for data collection, analysis and interpretation, and toolkit development. The four key constructs of Normalisation Process Theory will be applied to the PR context to understand how PR referral becomes embedded in clinical practice:

a) Coherence: how PR is understood by clinicians.
b) Cognitive participation: clinicians' buy-in and support for PR.
c) Collective action: the operational work required for PR referral.
d) Reflexive monitoring: how PR is appraised by clinicians and how work is reconfigured to enable it to happen.

Closely aligned to Normalisation Process Theory, Burden of Treatment Theory will be used to understand patients' experiences.[46] This theory provides a structural model of the relationship between treatment burden and patients'/carers' capacity to undertake work required to manage their condition, and helps to understand variations in healthcare utilisation and adherence.[47] Health service responses to reduce this burden will be understood through the Minimally Disruptive Medicine model, a patient-centred approach in which patients and professionals work together to advance patient goals whilst imposing the smallest possible treatment burden.[48] Key elements of Burden of Treatment Theory and Minimally Disruptive Medicine mirror those of Normalisation Process Theory and will inform understanding of how patients:

a) Make sense of their health conditions, tests and treatments.
b) Enrol support, plan to attend healthcare visits and enact self-care activities.
c) Operationalise the work of attending visits and self-managing their care.
d) Monitor, appraise and evaluate the worth of the work they are doing.

For peer review only - http://bmjopen.bmj.com/site/about/guidelines.xhtml

METHODS AND ANALYSIS

Study overview

The study comprises three phases:

Phase 1: Online survey of HCPs in general practice and online survey of PR providers
Phase 2: Qualitative research with patients, HCPs and commissioners
Phase 3: Toolkit development research

Figure 1 shows how the phases map to the research questions.

Phases 1 and 2 will address the topic areas listed below to identify facilitators and barriers to referral and uptake and explore current practice and ideas for improvement. The data collection and analysis during these phases will be informed by Normalisation Process Theory in order to explicate how current ways of working, and the impact of barriers and facilitators, are embedded and sustained in practice. The application of Normalisation Process Theory will also inform understanding of how ideas for improvement can be embedded and sustained. We will sample from four participant groups (patients, HCPs, PR providers and commissioners) in order to compare and contrast the perspectives and needs of these groups in relation to PR referral. The topic areas are:

- Influences on HCP decisions to refer to PR
- Reasons why patients take up or decline a referral
- Methods to make it easier to refer to PR and take up a referral
- Tools/strategies in use to prompt referral and uptake
- Support or training that would improve counselling of patients regarding PR
- Ways to ensure effective communication between primary care and PR providers regarding referrals
- Priorities and design considerations that the toolkit should address

Findings from Phases 1 and 2 will inform toolkit development in Phase 3. The final study output will be a toolkit tested in clinical practice for usability and acceptability which, if validated, will be ready for wider testing.

Phase 1: Online surveys of HCPs in general practice and of PR providers

The surveys enable a broad exploration of the topic areas. Pulmonary disease or long-term condition leads in all GP practices in the East of England (n = approximately 455) will be invited to complete the general practice survey by the Clinical Research Network Eastern. The study team will send invitations to all PR providers (n = 21) in the East of England PR Network to complete the PR provider survey.

Survey design is informed by Normalisation Process Theory and includes items adapted from the NoMAD tool.[49] We sought input to the design from practice nurses and PR providers and piloted each survey. Items are dichotomous, multiple response or Likert scales, supplemented by free text questions [see supplements 1 and 2]. We will invite HCPs to upload relevant resources that they

For peer review only - http://bmjopen.bmj.com/site/about/guidelines.xhtml

recommend. Surveys will be implemented on the Online Surveys platform[50] and a prize draw of £200 in online shopping vouchers will incentivise returns.

Quantitative data will be uploaded to SPSS (v 25) for descriptive analysis including frequencies and comparison of responses by different healthcare providers and levels of experience. General practice data will be compared by Clinical Commissioning Group area and mapped onto referral and uptake data from PR services where this is available.

Free text responses will be imported into NVivo12 software. Analysis will follow a 'framework' approach as described by Ritchie et al.[51] Constructs from Normalisation Process Theory will inform a deductive coding structure supplemented by inductive coding to allow unexpected findings to emerge. Codes will be grouped into categories and a working analytical framework developed which will be applied to all data. Data will be summarised by category in framework matrices on which the final interpretation will be based.

Phase 2: Qualitative research with patients, HCPs and commissioners

The purpose of the qualitative research is to enable an in-depth exploration of the topic areas with the following groups:

1. Patients who have accepted a PR referral (focus groups)
2. Patients who have declined a PR referral (interviews)
3. Patients who have not been referred to PR (interviews)
4. HCPs in primary care who refer patients to PR (interviews and/or focus groups)
5. Commissioners of PR services (interviews). NHS Commissioners are responsible for planning and purchasing healthcare services for their local populations.
6. Physiotherapists and/or nurses who deliver PR (focus group)

Inclusion criteria

Patients will be eligible if they are resident in the East of England, have a diagnosis of COPD with stable disease, are eligible for PR as defined by guideline recommendations[8] and able to read and write in English. Patients who have accepted a PR referral will be eligible if they have attended only one programme. In addition, we will conduct interviews and focus groups in two geographical areas specifically targeting patients of South Asian heritage. For these activities patients will be eligible if they do not have the ability to read or write in English but can communicate, read and write in Hindi or Urdu.

Sampling

We will begin by identifying four PR services within the East of England based on utilisation of commissioned places in 2014/15. We will select the two services with the highest utilisation rates and the two with the lowest utilisation rates and then apply convenience sampling to identify participants as follows:

Patients and HCPs: Following convenience sampling within the PR services and the general practices that refer to them, we will recruit patients who have accepted a PR referral, patients who have declined or not been referred to PR and HCPs in primary care who refer patients to PR. We will aim to achieve a gender balance amongst patients.

For peer review only - http://bmjopen.bmj.com/site/about/guidelines.xhtml

Commissioners: We will use convenience sampling to identify a commissioner of each of the four PR services.

PR providers: We will use convenience sampling within the East of England PR Network to identify PR providers across the region.

We will also select two geographical areas in the East of England that have populations of South Asian heritage. We will use convenience sampling within the PR services and general practices to identify patients of South Asian heritage who have accepted a PR referral, patients who have declined or not been referred to PR and HCPs in primary care who refer patients to PR. We will aim for gender balance amongst patients.

We envisage that it may be challenging to recruit some participants, including patients who have declined PR and clinicians in primary care with busy and demanding schedules, and so convenience sampling will allow us flexibility in achieving the required numbers of participants. To enhance validity, the final sample size will be determined by the need to reach data saturation. In a study of patients referred to PR Arnold et al.[52] achieved saturation with 24 patients. Table 1 shows an estimation of the sample sizes to be aimed for. If necessary more participants will be sought in order to achieve data saturation or the final number may be lower if saturation is achieved with a lower number.

**Participant group**	**Estimated sample size**
Patients who have accepted a PR referral (8 focus groups of up to 8 participants)	Up to 48
Patients who have declined a PR referral (interviews)	Up to 9
Patients who have not been referred to PR (interviews)	Up to 9
HCPs in primary care who refer patients to PR (interviews and/or focus groups)	Up to 34
Commissioners of PR services (interviews)	4
Physiotherapists and/or nurses who deliver PR (focus group)	Up to 10

Table 1: Estimated sample sizes for each group of participants

Focus groups have the benefit of drawing out shared experiences among participants and we have planned focus groups where we have a reasonable expectation of being able to convene sufficient numbers of participants. Where we do not expect this to be the case we have planned for interviews.

Recruitment `

Patients who have accepted a PR referral: Each PR service will identify eligible patients, issue the invitation to attend the focus group and confirm attendance.

Patients who have declined a PR referral or who have not been referred to PR: General practices will identify patients from their COPD register and the invitation will be made by a clinician known to the patient. Telephone interviews, home visits or an interview on general practice premises will be offered to encourage participation. Interested patients will agree to be contacted by the study team who will make arrangements for interview.

HCPs in primary care: Clinical Research Network Eastern will invite HCPs to participate. Interested practices will return an expression of interest form to the Clinical Research Network who will inform the study team. The team will then arrange the interview or focus group.

For peer review only - http://bmjopen.bmj.com/site/about/guidelines.xhtml

Commissioners of PR services: The study team will identify and invite commissioners with support from Clinical Research Network Eastern.

Physiotherapists/nurses who deliver PR: The study team will invite participation through the East of England PR network.

Informed consent

Study team researchers will take written informed consent prior to commencing interviews or focus groups. Consent will also be taken from carers, family members or friends who accompany participants as they may provide information about the participant during the course of the interview or focus group. Participants will be free to withdraw from the study at any time.

Data collection

Question guides for interviews and focus groups [supplements 3-8] are developed from the relevant topic areas. Patient focus groups will be held at community or health care venues and interviews will be at the patient's home, a health care venue or conducted by telephone. Focus groups in general practice may be challenging to organise due to competing work schedules and geographical location so we will offer one-to-one interviews as well, on general practice premises or by telephone. The PR provider focus group will be held at a health care venue and commissioner interviews at the commissioner's place of work or by telephone. Interviews and focus groups will be audio-recorded and transcribed verbatim.

Data analysis

Transcriptions and field notes will be imported into NVivo12 software for data management and analysis. Analysis will follow a 'framework' approach[51] Normalisation Process Theory constructs will inform a deductive coding structure supplemented by inductive coding to allow unexpected findings to emerge. Scripts will be read, re-read and coded by two researchers. Codes will be grouped into categories and a working analytical framework developed which will be applied to all transcripts. Data will be summarised by category in framework matrices on which the final interpretation will be based.

Quality criteria

Structured and standardised question guides will ensure consistency. Transcripts will be coded independently by two researchers. Reviewing and revising the analysis with a patient reference group will provide a credibility and reliability check.

Phase 3: Toolkit Development Research

The purpose of this phase is to synthesise findings from Phases 1 and 2 and incorporate the learning into the toolkit development.

User group

A user group of five HCPs and five patients recruited from research participants, volunteers from PPI events and British Lung Foundation networks will work with the study team, a web developer and an expert in design and development from the Engineering Design Centre at the University of Cambridge to develop the toolkit. We will offer a range of ways to participate in testing and feedback, e.g.

For peer review only - http://bmjopen.bmj.com/site/about/guidelines.xhtml

online, face-to-face meetings, visiting members, holding satellite meetings at professional conferences or service/training meetings. Written informed consent will be taken for all participants.

The design process

A rigorous, iterative design process (Explore, Create, Evaluate)[53] (figure 2) will track the user journey from initial concept to building the toolkit, including the development of criteria for acceptability and usability testing. To explore potential toolkit content and structure the needs, a review will take place of the summaries of themes identified from the survey and qualitative research. This will be underpinned by Normalisation Process Theory and Burden of Treatment theory constructs, and will be supported further by the outputs from the literature review and work by other expert groups e.g. the British Thoracic Society PR Quality Improvement Group. A consensus method adapted from nominal group technique[54] will be used to consider and vote on priorities. Content and media will be developed during the user-centred design process but we are aiming for a simple and practical toolkit with information about ways to impact referral and uptake. The toolkit is intended for direct use by HCPs in primary care with the aim of enhancing their understanding of PR, providing resources to support a meaningful and motivational conversation about PR with the patient and to facilitate the referral process. Examples could include key messages about the benefits of PR, electronic prompts/templates to prompt referral, links to guidelines and decision-support tools and printing options). It is important that we do not pre-empt the design process, in terms of content or medium, as this must be co-produced with users. However, based on what we know from the literature about barriers to referral and uptake we would envisage important features to include: clear information about PR and its benefits and eligibility criteria; prompts and tools to facilitate the referral process; tools to support an exploration of the patient's needs and beliefs about PR; tools and information to support the HCP to motivate patients and encourage uptake. The toolkit may also include information for patients that can be shared with them by the HCP. It is not intended that patients will access the toolkit directly. Design will initially be solution-neutral and will then be translated into practical solutions. Practical resource constraints will be considered and any user-defined needs that could not be addressed will be recorded. The group will also agree initial assessment criteria for usability and acceptability testing.

Agile methods[55] will be used to create the toolkit. The study team will build initial web pages in the live location to develop concepts and stimulate ideas. Early and subsequent releases of the toolkit will be continuously explored, created, evaluated and fed back upon with the user group, alongside continuous web development in short time cycles over six months. Usability and acceptability assessment criteria will determine the feedback requested and with each round of feedback the criteria will be open to further refinement as users explore and identify their practical needs. The toolkit will be built on a WordPress platform, a free open source website infrastructure and content management system, and hosted on University of Cambridge web servers by University Information Systems. A web development expert from the University of Cambridge will lead the technical development. It will be designed to interface with working systems (in ways to be identified during the research, e.g. linked through SystmOne,[56] integrated into annual review) and a flexible design will enable on-going development.

Data collection

During toolkit development subjective and objective data will be gathered from user group participants to assess the user-defined usability and acceptability criteria. The methods of testing acceptability and usability will be those best suited to measure those criteria. Therefore, at this stage,

For peer review only - http://bmjopen.bmj.com/site/about/guidelines.xhtml

we cannot pre-determine what those methods will be. However, we envisage considering a range of approaches including retrospective methods, e.g. written feedback and interviews, and real time methods, e.g. thinking aloud techniques and objective methods, e.g. user observation. Testing will take place after each iteration of the design process.

Following development we will work with five general practices to identify the HCP most involved in PR referrals and train them to use the toolkit for four weeks in a clinical setting. Feedback will be sought relating to the user-defined assessment criteria plus user experience, perceived understanding of PR and referral criteria, technical issues and nurse time to implement the toolkit. Each HCP will complete a short questionnaire following use of the toolkit with patients and an interview at the end of the four week period. Patients with whom the HCP has used the toolkit will complete a short evaluation questionnaire to assess the impact on the patient experience [supplement 9]. This will be returned directly to the study team with a stamped addressed envelope. Questionnaires will reflect the evaluation criteria. Written informed consent will be taken from HCPs and patients. During this testing period we will collect usage metrics from the system and data on referral numbers and uptake compared to baseline.

Data analysis

Quantitative data will be uploaded into Excel and descriptive analysis will be conducted to identify frequencies. Interviews will be audio recorded, transcribed and uploaded to NVivo 12 software for data management and analysis. Data analysis will follow procedures as described in Phase 2 with the addition that the user-defined usability and acceptability criteria, together with Normalisation Process Theory constructs, will inform the development of a deductive coding structure.

PATIENT AND PUBLIC INVOLVEMENT

We involved a range of people in patient and public involvement (PPI) activities to inform the study design and plain English communications. Forty-seven people from PR programmes and patient support groups attended across six meetings, we consulted four members of the Cambridge University Hospitals PPI panel and noted feedback from patient participants in meetings about the future of services throughout the Cambridgeshire and Peterborough healthcare system. A need for increased access to PR and the value of locating the study in primary care were seen as important. As a result we engaged a GP co-applicant to the study. PPI input is ongoing and we have established a PPI group, supported by a member of the study team, who will review research materials, feedback on the research analysis and support dissemination. We offer travel expenses to PPI meetings. The British Lung Foundation will also support active engagement of their patient members.

We also sought feedback on the research priorities from practice nurses at professional development meetings. They wanted easy access to good quality information and methods to engage patients, and recommended an emphasis on patient benefit, rather than cost savings, to encourage practice nurses to participate.

ETHICS AND DISSEMINATION

The study has been given a favourable opinion by the Cambridge Central Research Ethics Committee (REC reference number 17/EE/0136) and has been granted approval by the Health Research Authority. It is registered with the ISRCTN registry (trial ID: ISRCTN20669629, assignment date 20th March 2018, trial start date 1st April 2016).

For peer review only - http://bmjopen.bmj.com/site/about/guidelines.xhtml

The study involves minimal risks to patients; however patients may be vulnerable due to age or frailty and the nature of COPD symptoms, including breathlessness and fatigue. Participants will be able to take breaks and terminate an activity if they are unable to continue. We will enquire about special support needs and participants can bring a friend or carer to any activity. Experienced researchers with appropriate training will undertake interviews and focus groups.

Dissemination plan

We aim for timely dissemination, parallel to formal evaluation and clarifying how the outcomes will be of practical benefit to patients. Audiences include: patients and carers/families; HCPs who refer to PR and general practice managers; PR providers and service managers; PR commissioners; national interest groups, e.g. British Thoracic Society; NHS England and NHS Improvement.

We will use a range of channels, working closely with the British Lung Foundation to utilise patient networks, publications, online information, service development and HCP engagement. We will use routes that are accessible to patient audiences, e.g. Breathe Easy patient newsletters, INVOLVE website, posters in GP waiting rooms and PR classes, village newsletters. PPI volunteers will disseminate to patient groups and networks.

Methods will include: presentations and written information for HCP forums and professional networks; presentations at regional, national and international academic and professional conferences; open access academic papers in peer-reviewed journals; web and social media updates; written feedback to participants; broadcast and print media, e.g. radio and newspapers, may be considered if appropriate.

Data deposition and curation

The study complies with the General Data Protection Regulation (GDPR) (2018). Data will be anonymised and entered onto secure NHS computers and University of Cambridge computers for data analysis. Signed, paper consent forms containing participants' names will be stored in a locked filing cabinet on NHS premises. Only the study team will have access to participants' data. Electronic files will be password protected. Data sharing and storage will meet the requirements of the National Institutes of Health Research. Data will be securely stored in the University of Cambridge Research Repository.

For peer review only

For peer review only - http://bmjopen.bmj.com/site/about/guidelines.xhtml

ACKNOWLEDGEMENTS

We would like to thank the patients and their families who contributed to the PPI work that supported the development of this study. Thanks to Jenna Stockwell from the British Lung Foundation who is supporting the study and its implementation. Delivery of this work was supported by the Cambridge Biomedical Research Centre.

COMPETING INTERESTS STATEMENT

JW reports grants from National Institute for Health Research during the conduct of the study.

No further disclosures.

FUNDING STATEMENT

This paper presents independent research funded by the National Institute for Health Research (NIHR) under its Research for Patient Benefit (RfPB) Programme (Grant Reference Number PB-PG-1215-20034). The views expressed are those of the authors and not necessarily those of the NHS, the NIHR or the Department of Health.

AUTHOR STATEMENT

JF led the research team, supervised the development of the protocol, contributed to the design of the study and development of the protocol, and revised the draft manuscript. He is a guarantor for the work.

FE contributed to the design of the study and development of the protocol, and drafted and revised the manuscript.

PW contributed to the design of the study and development of the protocol, proposed the theoretical basis for the design and supervised development of the associated methodology and revised the draft manuscript.

CD contributed to the design of the study and development of the protocol and revised the draft manuscript. She is a guarantor for the work.

IW contributed to the design of the study and development of the protocol and revised the draft manuscript.

TD contributed to the design of the study and development of the protocol and proposed the toolkit development methodology.

JW contributed to the design of the study and development of the protocol, proposed the toolkit development methodology and revised the draft manuscript.

LJ contributed to the design of the study and development of the protocol.

RB contributed to the design of the study and development of the protocol.

SS contributed to the design of the study and development of the protocol.

JBenson contributed to the design of the study and development of the protocol.

For peer review only - http://bmjopen.bmj.com/site/about/guidelines.xhtml

JBrimicombe provided technical advice regarding building and using the platform for developing the toolkit and revised the draft manuscript.

LK contributed to the design of the study and development of the protocol.

HH contributed to the design of the study and development of the protocol and revised the draft manuscript.

For peer review only

For peer review only - http://bmjopen.bmj.com/site/about/guidelines.xhtml

REFERENCES

1. British Lung Foundation. Chronic Obstructive Pulmonary Disease (COPD) statistics http://statistics.blf.org.uk/copd. Accessed June 2018.
2. McLean S, Hoogendoorn M, Hoogenveen RT, et al. Projecting the COPD population and costs in England and Scotland: 2011 to 2030. *Sci Rep*. 2016;6:31893.doi:10.1038/srep31893.
3. McCarthy B, Casey D, Devane D et al. Pulmonary rehabilitation for chronic obstructive pulmonary disease (Review). *Cochrane Database Syst Rev* 2015(2):p.Art.No.:CD003793.doi:10.1002/14651858.CD003793.pub3.
4. Singh SJ, Zuwallack R, Garvey C et al. Learn from the past and create the future: the 2013 ATS/ERS statement on pulmonary rehabilitation. *Eur Respir J* 2013;42(5):1169-74.doi:10.1183/09031936.00207912.
5. Griffiths TL, Burr ML, Campbell IA et al. Results at 1 year of outpatient multidisciplinary pulmonary rehabilitation: a randomised controlled trial. *Lancet* 2000;355:362-368.doi:10.1016/S0140-6736(99)07042-7.
6. Seymour JM, Moore L, Jolley CJ et al. Outpatient pulmonary rehabilitation following acute exacerbations of COPD. *Thorax* 2010;65(5):423-428.doi:10.1136/thx.2009.124164.
7. Griffiths TL, Phillips CJ, Davies S, et al Cost effectiveness of an outpatient multidisciplinary pulmonary rehabilitation programme. *Thorax* 2001;56:779-784.doi :10.1136/thorax.56.10.779
8. National Institute for Health and Clinical Excellence. COPD Guideline CG101 2010 http://www.nice.org.uk/guidance/CG101. Accessed June 2018.
9. Bolton CE, Bevan-Smith EF, Blakey JD et al. The BTS Guideline on Pulmonary Rehabilitation in Adults. *Thorax* 2013;68:ii1-ii30.
10. Steiner M, Holzhauer-Barrie J, Lowe D, et al. Pulmonary Rehabilitation: Time to breathe better. National Chronic Obstructive Pulmonary Disease (COPD) Audit Programme: Resources and organisation of Pulmonary Rehabilitation services in England and Wales 2015. National organisational audit report. London: RCP, November 2015.
11. Barr RG, Celli BR, Martinez FJ et al. Physician and patient perceptions in COPD: the COPD Resource Network Needs Assessment Survey. *The American Journal Of Medicine* 2005;118(12):1415-1415.doi:10.1016/j.amjmed.2005.07.059.
12. Harris D, Hayter M, Allender S. Factors affecting the offer of pulmonary rehabilitation to patients with chronic obstructive pulmonary disease by primary care professionals: a qualitative study. *Prim Health Care Res Dev* 2008;9:280-290.doi:10.1017/S1463423608000832.
13. Yawn BP, Wollan PC. Knowledge and attitudes of family physicians coming to COPD continuing medical education. *Int J Chron Obstruct Pulmon Dis* 2008;3(2):311-317.doi:10.2147/COPD.S2486.
14. Johnston CL, Maxwell LJ, Maguire GP et al. How prepared are rural and remote health care practitioners to provide evidence-based management for people with chronic lung disease? *Aust J Rural Health* 2012;20(4):200-207.doi:10.1111/j.1440-1584.2012.01288.x
15. Johnston K, Grimmer-Somers K, Young M et al. Which chronic obstructive pulmonary disease care recommendations have low implementation and why? A pilot study. *BMC Res Notes* 2012;5:652-652.doi:10.1186/1756-0500-5-652
16. Perez X, Wisnivesky JP, Lurslurchachai L et al. Barriers to adherence to COPD guidelines among primary care providers. *Respir Med* 2012;106(3):374-381.doi:/10.1016/j.rmed.2011.09.010
17. Boulet L-P, Bourbeau J, Skomro R et al. Major care gaps in asthma, sleep and chronic obstructive pulmonary disease: a road map for knowledge translation. *Can Respir J* 2013;20(4):265-269.doi:10.1155/2013/496923
18. Desalu OO, Onyedum CC, Adeoti AO et al. Guideline-based COPD management in a resource limited setting - physicians' understanding, adherence and barriers: a cross-sectional survey of internal and family medicine hospital-based physicians in Nigeria. *Prim Care Respir J* 2013;22(1):79-85.doi:10.4104/pcrj.2013.00014
19. Hernandez P, Balter SM, Bourbeau J. et al. Canadian practice assessment in chronic obstructive pulmonary disease: respiratory specialist physician perception versus patient reality. *Can Respir J* 2013;20(2):97-105.doi:10.1155/2013/369019

For peer review only - http://bmjopen.bmj.com/site/about/guidelines.xhtml

20. Johnston K, Young M, Grimmer K et al. Frequency of referral to and attendance at a pulmonary rehabilitation programme amongst patients admitted to a tertiary hospital with chronic obstructive pulmonary disease. *Respirology* 2013;18(7):1089-1094.
21. van der Wees PJ, Zagers KAM, de Die SE et al. Developing a questionnaire to identify perceived barriers for implementing the Dutch physical therapy COPD clinical practice guideline. *BMC Health Serv Res* 2013;13:159-159.doi:10.1186/1472-6963-13-159
22. Walsh JR, McKeough ZJ, Morris NR et al. Performance-based criteria are used in participant selection for pulmonary rehabilitation programs. *Aust Health Rev* 2013;37(3):331-336.
23. Yohannes, A, Stone R, Lowe D et al. Pulmonary rehabilitation in the United Kingdom. *Chron Respir Dis* 2011;8(3): 193-199.doi:10.1071/AH12192
24. Jones RCM, Dickson-Spillman M, Mather MJC et al. Accuracy of diagnostic registers and management of chronic obstructive pulmonary disease: the Devon primary care audit. *Respir Res* 2008;9:62-62.doi:10.1186/1465-9921-9-62
25. Milner SC, Boruff JT, Beaurepaire C et al. Rate of, and barriers and enablers to, pulmonary rehabilitation referral in COPD: A systematic scoping review. *Respir Med* 2018;137:103-114.
26. Keating A, Lee A, Holland AE. What prevents people with chronic obstructive pulmonary disease from attending pulmonary rehabilitation? A systematic review. *Chron Respir Dis* 2011;8(2):89-99.doi:10.1177/1479972310393756
27. Thorpe O, Johnston K, Kumar S. Barriers and Enablers to Physical Activity Participation in Patients With COPD. A systematic review. *J Cardiopulm Rehab Prev* 2012;32:359-369.
28. Mathar H, Fastholme P, Hansen IR et al. Why Do Patients with COPD Decline Rehabilitation. *Scand J Caring Sci* 2015 DOI:10.1111/scs.12268.doi:10.1111/scs.12268
29. Angus RM, Thompson EB, Davies L et al. Feasibility and impact of a computer-guided consultation on guideline-based management of COPD in general practice. *Prim Care Respir J* 2012;21(4):425-430.doi:10.4104/pcrj.2012.00088
30. Foster F, Piggott R, Riley L, et al. Working with primary care clinicians and patients to introduce strategies for increasing referrals for pulmonary rehabilitation. *Prim Health Care Res Dev* 2016;17(3):226-37.doi:10.1017/S1463423615000286
31. Graves J, Sandrey V, Graves T, et al. Effectiveness of a group opt-in session on uptake and graduation rates for pulmonary rehabilitation. *Chron Respir Dis* 2010;7(3):159-64.doi:10.1177/1479972310379537
32. Hopkinson NS, Englebretsen C, Cooley N et al. Designing and implementing a COPD discharge care bundle. *Thorax*. 2012;67:90-92.doi:10.1136/thoraxjnl-2011-200233
33. Hull S, Mathur R, Lloyd-Owen S, et al. Improving outcomes for people with COPD by developing networks of general practices: evaluation of a quality improvement project in east London. *NPJ Prim Care Respir Med* 2014;24:14082.doi:10.1038/npjpcrm.2014.82
34. Roberts CM, Gungor G, Parker M, et al. Impact of a patient-specific co-designed COPD care scorecard on COPD care quality: a quasi-experimental study. *NPJ Prim Care Respir Med* 2015;25:15017.doi:10.1038/npjpcrm.2015.17
35. Lange P, Rasmussen FV, Borgeskov H et al. The quality of COPD care in general practice in Denmark: the KVASIMODO study. *Prim Care Resp J* 2007;16(3):174-181.doi:10.3132/pcrj.2007.00030
36. Lange P, Andersen KK, Munch E et al. Quality of COPD care in hospital outpatient clinics in Denmark: the KOLIBRI study. *Resp Med* 2009;103:1657-1662.doi:10.1016/j.rmed.2009.05.010
37. Tottenborg SS, Thomsen RW, Nielsen H, et al. Improving quality of care among COPD outpatients in Denmark 2008-2011. *Clin Resp J* 2013;7:319-327.doi:10.1111/crj.12009
38. Ulrik CS, Hansen EF, Jensen MS et al. Management of COPD in general practice in Denmark – participating in an educational program substantially improves adherence to guidelines. *Int J COPD* 2010;5:73-79.
39. Harris M, Smith BJ, Veale AJ, et al. Providing reviews of evidence to COPD patients: controlled prospective 12-month trial. *Chron Respir Dis* 2009;6(3):165-73.doi:10.1177/1479972309106577
40. Zwar NA, Hermiz O, Comino E et al. Care of patients with a diagnosis of chronic obstructive pulmonary disease: a cluster randomised controlled trial. *Med J Aust* 2012Oct 1;197(7):394-8.doi:10.5694/mja12.10813

For peer review only - http://bmjopen.bmj.com/site/about/guidelines.xhtml

41. Deprez R, Kinner A, Millard P, et al. Improving Quality of Care for Patients with Chronic Obstructive Pulmonary Disease. *Popul Health Manage* 2009;12(4):209-215.doi:10.1089/pop.2008.0043
42. Jones AW, Taylor A, Gowler H, et al. Systematic review of interventions to improve patient uptake and completion of pulmonary rehabilitation in COPD. *ERJ Open Res* 2017;3:00089-2016.doi:10.1183/23120541.00089-2016
43. Early F, Wellwood I, Kuhn I et al. Interventions to increase referral and uptake to Pulmonary Rehabilitation in people with Chronic Obstructive Pulmonary Disease (COPD): a systematic review. *Int J COPD* in press.
44. May C, Finch T. Implementing, Embedding, and Integrating Practices: An Outline of Normalization Process Theory. *Sociology* 2009; 43(3):535-554.doi:10.1177/0038038509103208
45. May C, Finch T, Ballini L, et al. Evaluating complex interventions and health technologies using normalization process theory: development of a simplified approach and web-enabled toolkit. *BMC Health Serv Res* 2011;11(1):245.doi:10.1186/1472-6963-11-245
46. May CR et al. Rethinking the patient: using Burden of Treatment Theory to understand the changing dynamics of illness. *BMC Health Serv Res* 2014;14:281.doi:10.1186/1472-6963-14-281
47. Demain S, Gonçalves AC, Areia C et al. Living With, Managing and Minimising Treatment Burden in Long Term Conditions: A Systematic Review of Qualitative Research. *PLoS One* 2015;10(5):e0125457.doi:10.1371/journal.pone.0125457.doi:10.1371/journal.pone.0125457
48. Leppin AL, Montori VM, Gionfriddo MR. Minimally Disruptive Medicine: A Pragmatically Comprehensive Model for Delivering Care to Patients with Multiple Chronic Conditions. *Healthcare (Basel)* 2015;3:50-63.doi:10.3390/healthcare3010050
49. NoMAD ref: Finch TL, Girling M, May CR, Mair FS, Murray E, Treweek S, Steen IN, McColl EM, Dickinson C, Rapley T (2015) NoMad: Implementation measure based on Normalisation Process theory [Measurement Instrument]. Retrieved from http://www.normalizationprocess.org/nomad-study/. Accessed June 2018.
50. Online Surveys. https://www.onlinesurveys.ac.uk/ Accessed June 2018.
51. Ritchie J, Lewis J, McNaughton Nicholls C, Ormston R: Qualitative research practice: a guide for social science students and researchers. 2nd ed. London: Sage; 2014
52. Arnold E, Bruton A, Ellis-Hill C. Adherence to pulmonary rehabilitation: A qualitative study. *Respir Med* 2006;100(10):1716-1723.doi:10.1016/j.rmed.2006.02.007
53. Clarkson PJ, Coleman R, Hosking I, Waller SD, 2011. Inclusive Design Toolkit, second ed. www.inclusivedesigntoolkit.com. Accessed May 2018.
54. Delbecq AL, van de Ven AH. A group process model for problem identification and program planning. *J Appl Behav Sci* 1971;7(4):466–91.doi.org/10.1177/002188637100700404
55. Agile manifesto. http://agilemanifesto.org/principles.html Accessed June 2018.
56. SystmeOne. https://www.tpp-uk.com/products/systmone Accessed June 2018.

For peer review only - http://bmjopen.bmj.com/site/about/guidelines.xhtml

Figures

Figure 1: Mapping of research questions and phases

Figure 2: The fundamental questions of design are solved through successive cycles of exploring needs, creating concepts and evaluating options, guided by project management.[51]

For peer review only

For peer review only - http://bmjopen.bmj.com/site/about/guidelines.xhtml

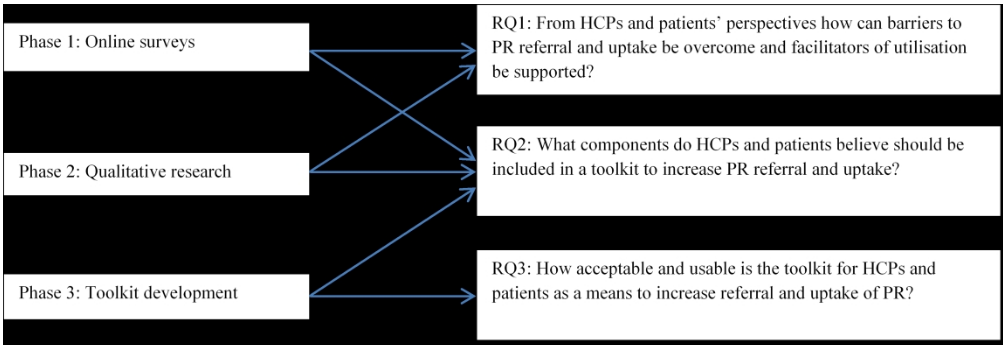

347x118mm (300 x 300 DPI)

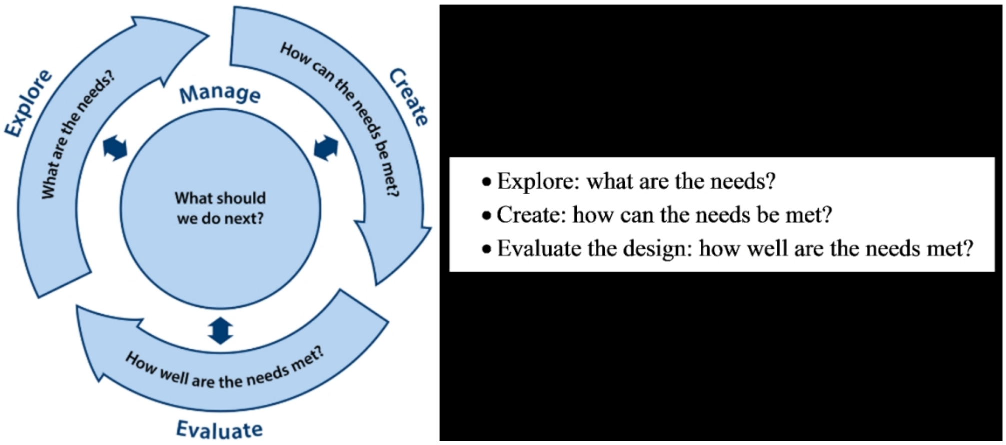

127x55mm (300 x 300 DPI)

Cambridge University Hospitals NHS
NHS Foundation Trust

The REsTORE project: Increasing REferral and uptake TO Pulmonary REhabilitation (general practice survey)

Page 1: Introduction

Thank you for taking part in this survey. The aim is to understand blocks and facilitators to referral and uptake of pulmonary rehabilitation (PR) in primary care and how referral and uptake can be improved.

The survey is part of a research study funded by the National Institute for Health Research (Research for Patient Benefit Programme) that aims to increase the number of people taking up PR. The study also includes interviews and focus groups with patients and clinicians to help us understand what happens during the referral process. We will use what we have learned to develop a toolkit of resources to support referral in primary care, working collaboratively with patients and clinicians.

We request that the survey is completed by a member of clinical staff in your practice who is involved with PR referrals.

The survey should take about 10 to 20 minutes. There are four parts:

- Your role
- Your experience of the PR referral process
- Improving referral and uptake of PR
- Getting in touch

There are no right or wrong answers. All responses and comments are valuable to us. At the end you will be able to leave any additional comments you wish to make.

Your response is valuable to us and so your practice will be paid £10 for the clinical staff

For peer review only - http://bmjopen.bmj.com/site/about/guidelines.xhtml

time needed to complete it. In addition, we invite you to enter a prize draw to win £200 in online shopping vouchers as an incentive to take part. The project is registered on the NIHR CRN Portfolio and completion will count as an accrual for your practice.

On the next two pages you will find participant information about the survey and a consent form for your participation. Please complete the consent form before continuing.

Please complete the survey as soon as possible.

Thank you for your participation.

For peer review only

I confirm that I am a member of clinical staff in my practice who is involved with referral to pulmonary rehabilitation * *Required*

- ○ Yes

For peer review only - http://bmjopen.bmj.com/site/about/guidelines.xhtml

Page 2: Participant Information

You are invited to take part in an online survey as part of a project to help increase referral and uptake to pulmonary rehabilitation for people with Chronic Obstructive Pulmonary Disease (COPD).

1. What is the purpose of the study?

Our study aims to increase the number of people with COPD who attend pulmonary rehabilitation by developing an online toolkit to help GP practices refer patients successfully. Pulmonary rehabilitation offers supervised exercise and education and helps people to manage their COPD symptoms, improve their quality of life and avoid admission to hospital. It is recommended by NICE. However, the National COPD Audit Programme reported that in 2013/14 an estimated 446,000 COPD patients in England and Wales were eligible for pulmonary rehabilitation but that only 68,000 referrals were made. Of those, only 69% took up the referral offer.

This survey of healthcare professionals (HCPs) in primary care who care for people with COPD is the first step in developing the toolkit. We are also surveying pulmonary rehabilitation providers. Following the survey we will hold interviews and focus groups with patients and HCPs to gather ideas about how to make referral easier and how to help patients make informed decisions about whether to attend pulmonary rehabilitation. We will combine these ideas with evidence from published research and then work with patients and HCPs to assemble the information in an online toolkit. The toolkit could contain, for example, patient-friendly information, electronic reminders on patient records or simplified referral processes. It will be designed to integrate with primary care working practices and to be used, for example, during an annual COPD review. We will test the toolkit in primary care to ensure it is practical for the NHS and has the potential to make a difference.

2. Why have I been chosen to take part in the survey?

We are inviting members of clinical staff in general practices in the East of England who are involved in referral to pulmonary rehabilitation.

3. What will I need to do?

The survey will take about 10 to 20 minutes to complete and is designed to be quick and easy. It will ask about your experiences of referring patients to pulmonary rehabilitation and for your ideas about how to improve referral and uptake. At the end of the survey, we will invite you to provide your contact details if you would like to take part in a subsequent interview or focus group on this topic.

For peer review only - http://bmjopen.bmj.com/site/about/guidelines.xhtml

4. How will the findings be used?

The survey results will be reported in a way that preserves confidentiality and you will not be identified in any way. The results may be published on the Addenbrooke's Hospital intranet, in peer reviewed medical journals, and used for medical presentations, conferences or presentations to patient groups. If you would like to be kept informed of the results please contact the Research Manager, Dr Frances Early, using the details below.

5. What will happen to the information I provide?

All information that is collected is strictly confidential. Only members of the research team will have access to the information. Where data are referred to in published material participants will not be identifiable. Data will be held in secure storage and destroyed after five years. Any information that you give will be used for research purposes only and you may ask to see your personal information at any time.

6. Can I withdraw from the project?

You may withdraw from the study at any time without giving a reason. If you withdraw we will only retain and use any personal information you have provided up to that point if you give us permission to do so.

7. Who has organised the research?

The Lead Investigator for the study is Dr Jonathan Fuld at Cambridge University Hospitals NHS Foundation Trust. Other members of the research team are:

Dr Frances Early (Cambridge University Hospitals NHS Foundation Trust)

Professor Christi Deaton, Dr Ian Wellwood, Dr John Benson, Dr Lois Kim (Cambridge Institute of Public Health)

Ruth Barlow, Lianne Jongepier (East of England Pulmonary Rehabilitation Network)

Professor Patricia Wilson (University of Kent)

Professor Sally Singh (University Hospitals of Leicester NHS Foundation Trust)

The British Lung Foundation

8. How has the study been funded?

The research is funded by the National Institute for Health Research as part of their Research for Patient Benefit programme (award no: PB-PG-1215-20034).

For peer review only - http://bmjopen.bmj.com/site/about/guidelines.xhtml

9. Further information and contact details

If you would like more information please contact Dr Frances Early, Box 146, Cambridge University Hospitals NHS Foundation Trust, Hills Road, Cambridge, CB2 0QQ. Tel 01223 274858. Email: frances.early@addenbrookes.nhs.uk

Ref (WP2 A2 PIS Survey Primary Care v1.1 260617) IRAS ID 209597

For peer review only

Page 3: Consent Form

Please tick each box below to indicate that you understand and agree with the statements

- ☐ I confirm that I have read and understood the information sheet on page 2 of this survey. I have had the opportunity to consider the information, ask questions and have had questions answered satisfactorily
- ☐ I understand my participation is voluntary and I may withdraw at any time without giving any reason
- ☐ I agree that the information I provide can be used solely for the purpose of this reasearch
- ☐ I agree to the use of anonymised data in publications
- ☐ I understand that all personal information will remain confidential
- ☐ I agree to take part in the above study

I confirm my agreement to the above statements ✱ *Required*

For peer review only

Page 4: Your role

Which best describes your professional job category?

- ○ Practice nurse
- ○ Nurse practitioner
- ○ Advanced nurse practitioner
- ○ Nurse consultant
- ○ Specialist respiratory nurse
- ○ Postgraduate diploma in respiratory care
- ○ GP
- ○ Other

If other, please give details

What responsibilities do you have for the care of people with COPD? Tick as many as apply.

- ☐ Annual reviews
- ☐ Urgent assessments
- ☐ Ongoing management
- ☐ Spirometry
- ☐ Oxygen therapy
- ☐ Admission prevention
- ☐ Early discharge facilitation
- ☐ Prescribing
- ☐ Medication checks
- ☐ Opportunistic non-urgent care
- ☐ Other

If other, please give details

For peer review only - http://bmjopen.bmj.com/site/about/guidelines.xhtml

How many years' experience do you have of caring for people with respiratory problems?

Please enter a whole number (integer).

How many years have you worked in primary care?

Please enter a whole number (integer).

Please tell us the name and postcode of your general practice so that we can manage the payment to your practice and identify the list size and COPD register details from public sources

For peer review only

Page 5: The pulmonary rehabilitation (PR) referral process (i)

To which PR services does your general practice refer patients? Tick as many as apply.

- ☐ Norfolk Community (Norfolk Community Health and Care NHS Trust)
- ☐ West Norfolk (BOC Clinical Services)
- ☐ Norfolk & Norwich (Norfolk & Norwich University Hospital NHS Trust)
- ☐ Suffolk (Suffolk Community Healthcare)
- ☐ Great Yarmouth & Waveney: BEET (James Paget University Hospitals NHS FT)
- ☐ West Essex (Essex Partnership University NHS FT)
- ☐ South West Essex (NE London NHS FT)
- ☐ Mid Essex (Provide)
- ☐ North East Essex (Anglian Community Enterprise)
- ☐ Southend (Southend University Hospital NHS FT)
- ☐ Colchester Hospital (Colchester Hospital University NHS FT)
- ☐ Huntingdon & Brookfield (Cambridgeshire & Peterborough NHS FT)
- ☐ Cambridgeshire & Peterborough (Provide)
- ☐ Peterborough and Stamford (Peterborough and Stamford Hospitals NHS FT)
- ☐ Papworth Hospital (Papworth Hospital NHS FT)
- ☐ Luton & Dunstable (Luton & Dunstable University Hospital NHS FT)
- ☐ Luton Community (Cambridgeshire Community NHS FT)
- ☐ Bedford Hospital (Bedford Hospital NHS Trust)
- ☐ East & North Herts (Hertfordshire Community NHS Trust)
- ☐ West Herts (Central London Community Healthcare NHS Trust)
- ☐ Other

If other, please give details

For peer review only - http://bmjopen.bmj.com/site/about/guidelines.xhtml

I feel sure that I understand the eligibility criteria for PR contained in the BTS Guideline on Pulmonary Rehabilitation in Adults

Not at all	☐	☐	☐	☐	☐	☐	☐	☐	☐	☐	☐	Completely

I feel adequately prepared to refer patients to PR

Not at all	☐	☐	☐	☐	☐	☐	☐	☐	☐	☐	☐	Completely

Referring patients to PR is currently a normal part of my work

Not at all	☐	☐	☐	☐	☐	☐	☐	☐	☐	☐	☐	Completely

I find the process of referring patients to PR is easy

Not at all easy	☐	☐	☐	☐	☐	☐	☐	☐	☐	☐	☐	Very easy

What resources (e.g. tools, systems or processes) are available in your general practice to support clinicians in making PR referrals? Tick all that apply.

- ☐ Prompts in clinical system
- ☐ Information about PR for clinicians
- ☐ Clear referral criteria

For peer review only - http://bmjopen.bmj.com/site/about/guidelines.xhtml

- [ ] Electronic referral
- [ ] Skills training for communicating effectively with patients
- [ ] Other

If other, please give details

What resources (e.g. tools, systems or processes) are available in your general practice to support patients in deciding whether to take up a referral? Tick all that apply.

- [ ] Information about PR for patients
- [ ] Shared decision-making tools
- [ ] Other

If other, please give details

For peer review only

For peer review only - http://bmjopen.bmj.com/site/about/guidelines.xhtml

Page 6: The pulmonary rehabilitation (PR) referral process (ii)

What factors influence your decision to refer a patient to PR?

	No influence	Some influence	Strong influence
Increasing shortness of breath	☐	☐	☐
MRC Dyspnoea Scale ≥3	☐	☐	☐
Mobility is affected by breathlessness	☐	☐	☐
Decreasing activity levels	☐	☐	☐
Frequent exacerbations	☐	☐	☐
Recent hospital discharge after exacerbation	☐	☐	☐
Patient is deconditioned	☐	☐	☐
Low exercise tolerance	☐	☐	☐
Patient anxiety	☐	☐	☐
Poor self-management	☐	☐	☐
Easy to use referral process	☐	☐	☐
Other	☐	☐	☐

If other, please give details

What factors might influence your decision *not* to refer an eligible patient to PR?

	No influence	Some influence	A strong influence
I don't have enough information about PR	☐	☐	☐

For peer review only - http://bmjopen.bmj.com/site/about/guidelines.xhtml

I'm uncertain that PR is worthwhile	☐	☐	☐
Patient has doubts that PR is worthwhile	☐	☐	☐
Patient refuses referral	☐	☐	☐
Patient co-morbidities	☐	☐	☐
Poor mobility	☐	☐	☐
Patient does not fit acceptance criteria for a programme	☐	☐	☐
Patient has attended PR previously	☐	☐	☐
Transportation problems	☐	☐	☐
Distance of class from patient's home	☐	☐	☐
Lack of time to make referral	☐	☐	☐
Timing of classes not convenient for patient	☐	☐	☐
I'm unaware of how to refer	☐	☐	☐
Difficult referral process	☐	☐	☐
Other	☐	☐	☐

If other, please give details

For peer review only - http://bmjopen.bmj.com/site/about/guidelines.xhtml

Page 7: The pulmonary rehabilitation (PR) referral process (iii)

For each statement please select the answer that best suits your experience

	Strongly disagree	Disagree	Not sure	Agree	Strongly agree
Staff in my general practice involved in the care of people with respiratory problems have a shared understanding of the purpose of PR	☐	☐	☐	☐	☐
I can see the potential value of referring patients to PR	☐	☐	☐	☐	☐
In my general practice there are key people who drive PR referral forwards and get others involved	☐	☐	☐	☐	☐
I believe that referring patients to PR is a legitimate part of my role	☐	☐	☐	☐	☐
I'm open to working with colleagues in new ways to enable PR referral to happen	☐	☐	☐	☐	☐

For peer review only - http://bmjopen.bmj.com/site/about/guidelines.xhtml

I can easily integrate PR referral into my work	☐	☐	☐	☐	☐
I have confidence in the ability of other colleagues who care for people with respiratory problems to refer patients to PR	☐	☐	☐	☐	☐
In my general practice PR referral is assigned to those with appropriate skills	☐	☐	☐	☐	☐
Sufficient training is provided in my practice so that staff know who to refer to PR	☐	☐	☐	☐	☐
Sufficient training is provided in my practice so that staff know how to refer to PR	☐	☐	☐	☐	☐
Sufficient resources are available to support PR referral in my practice	☐	☐	☐	☐	☐
I am aware of reports in my practice about the outcomes for patients following a PR referral	☐	☐	☐	☐	☐

For peer review only - http://bmjopen.bmj.com/site/about/guidelines.xhtml

The staff in my practice agree that referring patients to PR is worthwhile	☐	☐	☐	☐	☐
I am able to overcome any difficulties I have with regard to referring patients to PR	☐	☐	☐	☐	☐

For peer review only

For peer review only - http://bmjopen.bmj.com/site/about/guidelines.xhtml

Page 8: The pulmonary rehabilitation (PR) referral process (iv)

In your experience what are the main reasons that patients take up a referral to PR?

In your experience what are the main reasons why patients decline an offer of PR and do not wish to be referred?

In your experience what are the main reasons that patients referred to PR do not attend their assessment?

For peer review only - http://bmjopen.bmj.com/site/about/guidelines.xhtml

Page 9: Improving referral and uptake of pulmonary rehabilitation (PR)

What would make it easier for you to refer patients to PR?

What could be done to encourage more patients to take up a referral to PR?

Do you receive adequate communication from providers of PR about their PR programmes?

- Yes
- No

Please add comments to clarify if necessary

What do you think is important for effective communication between PR providers

For peer review only - http://bmjopen.bmj.com/site/about/guidelines.xhtml

and primary care?

Which communication methods are effective for you when communicating with PR providers? Select all that apply.

- ☐ Email
- ☐ Letter
- ☐ Telephone
- ☐ Other

If other, please give details

This research project aims to design and build an online toolkit of resources for use in primary care to support referral to PR. What do you think such a toolkit would need to contain or do in order to be successfully adopted by clinicians in primary care?

For peer review only - http://bmjopen.bmj.com/site/about/guidelines.xhtml

Page 10: Other comments

Please leave any other comments you would like to add

For peer review only

For peer review only - http://bmjopen.bmj.com/site/about/guidelines.xhtml

Page 11: Getting in touch

Do you have any resources that support PR referral or uptake that you would be willing to share with the research team?

- ◯ Yes
- ◯ No

Would you like to hear about other ways to get involved in this research study, e.g. developing the toolkit or testing it in practice?

- ◯ Yes
- ◯ No

Thank you for completing this survey. All responses are confidential and results will be reported anonymously. Would you like us to share the results with you?

- ◯ Yes
- ◯ No

Would you like to be entered for the prize draw to win £200 of online shopping vouchers?

- ☐ Yes
- ☐ No

If you answered 'yes' to any of the above questions please leave your contact details here or, if you prefer to keep your responses anonymous, please email the research team directly: frances.early@addenbrookes.nhs.uk

For peer review only - http://bmjopen.bmj.com/site/about/guidelines.xhtml

For peer review only

For peer review only - http://bmjopen.bmj.com/site/about/guidelines.xhtml

Page 12: The survey is now complete. Thank you for your time.

Key for selection options

3 - I confirm my agreement to the above statements

Yes

No

For peer review only

Cambridge University Hospitals NHS
NHS Foundation Trust

The REsTORE project: Increasing REferral and uptake TO pulmonary REhabilitation (PR provider survey)

Page 1: Introduction

Thank you for taking part in this survey of pulmonary rehabilitation (PR) providers. The aims are to understand the PR referral process, the experiences of PR providers, barriers and facilitators to referral and uptake of PR from primary care and how patients can be encouraged to take up a referral.

The survey is part of a research study funded by the National Institute for Health Research (Research for Patient Benefit Programme) that aims to increase the number of people taking up PR. The study also includes a survey of general practices, and interviews and focus groups with patients and clinicians to help us understand what happens during the referral process. We will use what we have learned to develop a set of resources (a 'toolkit') to support referral in primary care, working collaboratively with patients and clinicians.

We request that the survey is completed by a member of clinical staff in your organisation who is most involved with receiving referrals and assessing respiratory patients.

Your responses are valuable to us and so your service will be paid £10 for the clinical staff time to complete it. In addition, we invite you to enter a prize draw to win £200 in online shopping vouchers as an incentive to take part. The project is registered on the NIHR CRN Portfolio and completion will count as an accrual for your organisation if you are within the NHS.

The survey should take about 10 to 20 minutes. Items include:

- Participant information and consent
- Your role
- Your pulmonary rehabilitation service
- Your experience of the PR referral process
- Improving referral and uptake of PR
- Getting in touch

There are no right or wrong answers. All responses and comments are helpful. At the end you will be able to leave any additional comments you wish to make.

On the next two pages you will find participant information about the survey and a consent form for your participation. Please complete the consent form before continuing.

Thank you for your participation.

I confirm that I am a member of clinical staff involved in receiving referrals and assessing patients for pulmonary rehabilitation

○ Yes

For peer review only - http://bmjopen.bmj.com/site/about/guidelines.xhtml

Page 2: Participant Information

You are invited to take part in an online survey as part of a project to help increase referral and uptake to Pulmonary Rehabilitation (PR) for people with Chronic Obstructive Pulmonary Disease (COPD).

1. What is the purpose of the study?

Our study aims to increase the number of people with COPD who attend PR by developing an online toolkit to help GP practices refer patients successfully. The National COPD Audit Programme reported that in 2013/14 an estimated 446,000 COPD patients in England and Wales were eligible for PR but that only 68,000 referrals were made. Of those, only 69% took up the referral offer.

This survey of PR providers is the first step in developing the toolkit. We are also surveying healthcare professionals (HCPs) in primary care who care for people with COPD. Following the survey we will hold interviews and focus groups with patients and HCPs to gather ideas about how to make referral easier and how to help patients make informed decisions about whether to attend PR. We will combine these ideas with evidence from published research and then work with patients and HCPs to assemble the information in an online toolkit. The toolkit could contain, for example, patient-friendly information, electronic reminders on patient records or simplified referral processes. It will be designed to integrate with primary care working practices and to be used, for example, during an annual COPD review. We will test the toolkit in primary care to ensure it is practical for the NHS and has the potential to make a difference.

2. Why have I been chosen?

We are inviting PR providers in the East of England to take part in the survey.

3. What will I need to do?

The survey will take about 10 to 20 minutes to complete and is designed to be quick and easy. It will ask about your experiences of referring patients to PR and for your ideas about how to improve referral and uptake. At the end of the survey, we will invite you to provide your contact details if you would like to take part in a subsequent interview or focus group on this topic.

4. How will the findings be used?

The survey results will be reported in a way that preserves confidentiality and you will not be identified in any way. The results may be published on the Addenbrooke's Hospital intranet, in peer reviewed medical journals, and used for medical presentations, conferences or presentations to patient groups. If you would like to be kept informed of the results please contact the Research Manager, Dr Frances Early, using the details below.

5. What will happen to the information I provide?

All information that is collected is strictly confidential. Only members of the research team will have access to the information. Where data are referred to in published material participants will not be identifiable. Data will be held in secure storage and destroyed after five years. Any information that you give will be used for research purposes only and you may ask to see your personal information at any time.

6. Can I withdraw from the project?

You may withdraw from the study at any time without giving a reason. If you withdraw we will only retain and use any personal information you have provided up to that point if you give us permission to do so.

7. Who has organised the research?

The Lead Investigator for the study is Dr Jonathan Fuld at Cambridge University Hospitals NHS Foundation Trust. Other members of the research team are:

Dr Frances Early (Cambridge University Hospitals NHS Foundation Trust)

Professor Christi Deaton, Dr Ian Wellwood, Dr John Benson, Dr Lois Kim (Cambridge Institute of Public Health)

Ruth Barlow, Lianne Jongepier (East of England Pulmonary Rehabilitation Network)

Professor Patricia Wilson (University of Kent)

Professor Sally Singh (University Hospitals of Leicester NHS Foundation Trust)

The British Lung Foundation

8. How has the study been funded?

The research is funded by the National Institute for Health Research as part of their Research for Patient Benefit programme (award no: PB-PG-1215-20034).

9. Further information and contact details

If you would like more information please contact Dr Frances Early, Box 146, Cambridge University Hospitals NHS Foundation Trust, Hills Road, Cambridge, CB2 0QQ. Tel 01223 274858. Email@ frances.early@addenbrookes.nhs.uk

(Participant information sheet WP2 A4 PIS Survey Pul Rehab v1.1 090717) IRAS ID 209597

For peer review only - http://bmjopen.bmj.com/site/about/guidelines.xhtml

Page 3: Participant Consent

Please tick each box below to indicate that you understand and agree with the statements

- ☐ I confirm that I have read and understood the above information sheet (WP2 A4 PIS Survey Pul Rehab v1.1 090717). I have had the opportunity to consider the information, ask questions and have had questions answered satisfactorily.
- ☐ I understand my participation is voluntary and I may withdraw at any time without giving any reason
- ☐ I agree that the information I provide can be used solely for the purpose of this research
- ☐ I agree to the use of the anonymised data in publications
- ☐ I understand that all personal information will remain confidential
- ☐ I agree to take part in the above study

I confirm my agreement to the above statements ✱ *Required*

Page 4: Your organisation

Please tell us the name and contact details of your PR provider organisation so that we can manage the payment for completion of this survey

For peer review only - http://bmjopen.bmj.com/site/about/guidelines.xhtml

Page 5: Please tell us about your role so that we can understand who is involved in delivering pulmonary rehabilitation

What best describes your professional job category?

- ○ Physiotherapist
- ○ Nurse
- ○ Health support worker
- ○ Health care assistant
- ○ Occupational therapist
- ○ Dietician
- ○ Fitness instructor/trainer
- ○ Pharmacist
- ○ Other

If other, please give details

How many years have you been involved in the care of people with respiratory problems?

Please enter a whole number (integer).

What is your role in pulmonary rehabilitation? Please select all that apply.

- ☐ I manage the PR service
- ☐ I manage the PR team
- ☐ I run a PR class
- ☐ I assist with a PR class
- ☐ I work in the admin team and screen the PR referrals
- ☐ Other

If other, please give details

How many years have you worked in your pulmonary rehabilitation role?

Please enter a whole number (integer).

What is the WTE (Whole Time Equivalent) of your post?

What band or grade is your post?

For peer review only - http://bmjopen.bmj.com/site/about/guidelines.xhtml

For peer review only - http://bmjopen.bmj.com/site/about/guidelines.xhtml

Page 6: Please tell us about your pulmonary rehabilitation service

What type of organisation provides your PR service?

- ☐ NHS Trust or health board
- ☐ Community Interest Company (CIC)
- ☐ Private healthcare provider
- ☐ Charity
- ☐ Research
- ☐ Other

If other, please give details

Is your service open to referrals all year round?

- ○ Yes
- ○ No

Do you accept referrals from

- ○ Primary care
- ○ Secondary care
- ○ Primary and secondary care
- ○ Self-referral
- ○ Other

If other, please give details

What type of PR programmes do you offer?

- ☐ Rolling
- ☐ Cohort
- ☐ Rolling and cohort
- ☐ Other

If rolling, how many rolling programmes per year?

If cohort, how many cohorts per year?

If other, please give details

Do you offer group-based and/or one-to-one programmes?

- ☐ Group-based
- ☐ One-to-one
- ☐ Group-based and one-to-one
- ☐ Other

If other, please give details

In which settings does your service hold classes? Tick all that apply.

- ☐ Community settings
- ☐ Hospital settings
- ☐ Primary care settings
- ☐ Home settings
- ☐ Other

If other, please give details

At how many sites does your service offer PR?

Please enter a whole number (integer).

Do you offer initial assessment before enrolment on to PR?

- ○ Yes
- ○ No

How many exercise sessions do you offer for each course of PR (excluding assessment visits)?

Please enter a whole number (integer).

For peer review only - http://bmjopen.bmj.com/site/about/guidelines.xhtml

Page 7: Please tell us about the funding and capacity of your pulmonary rehabilitation service

How is your PR service funded?

- ☐ CCG commissioned
- ☐ Hospital funded
- ☐ Post-discharge rehabilitation tarrif
- ☐ Other

If other, please give details

Does your PR service have a fixed term of funding?

- ○ Yes
- ○ No
- ○ Don't know

If yes, how many years future funding does the service have? Please leave blank if you are not sure.

How many available places did your service have last year?

Please enter a number.

How many places did you fill in the last year (2016/17)?

Please enter a number.

What is the maximum number of places (across all sites) that your PR service can offer in the current financial year (2017-18) based on your staffing levels?

Please tick here if you are not sure

- ○ I'm not sure what the number of places is
- ○ I have given an estimate below

Estimated number of places

Please add any comments for clarification regarding funding or capacity

For peer review only - http://bmjopen.bmj.com/site/about/guidelines.xhtml

Page 8: Your experience of the pulmonary rehabilitation referral process (part i)

I understand the eligibility criteria for PR contained in the BTS Guideline on Pulmonary Rehabilitation in Adults

Please don't select more than 1 answer(s) per row.

	0	1	2	3	4	5	6	7	8	9	10	
Not at all	☐	☐	☐	☐	☐	☐	☐	☐	☐	☐	☐	Completely

The process of accepting patients onto a PR programme following referral is:

Please don't select more than 1 answer(s) per row.

	0	1	2	3	4	5	6	7	8	9	10	
Very difficult	☐	☐	☐	☐	☐	☐	☐	☐	☐	☐	☐	Very easy

I feel adequately prepared to accept patient referrals to PR

Please don't select more than 1 answer(s) per row.

	0	1	2	3	4	5	6	7	8	9	10	
Not at all	☐	☐	☐	☐	☐	☐	☐	☐	☐	☐	☐	Completely

For peer review only - http://bmjopen.bmj.com/site/about/guidelines.xhtml

Page 9: Your experience of the PR referral process (part ii)

Thinking of your work at the moment how strongly do you agree with the following statements?

Please don't select more than 1 answer(s) per row.

	Strongly disagree	Disagree	Not sure	Agree	Strongly agree
Staff in my organisation have a shared understanding of the purpose of PR	☐	☐	☐	☐	☐
There are key people in my organisation who promote PR	☐	☐	☐	☐	☐
I am open to working with colleagues in new ways to enable PR referral to happen	☐	☐	☐	☐	☐
I have confidence in clinicians' ability to refer patients to PR	☐	☐	☐	☐	☐
Work involved in processing PR referrals is assigned to people in my organisation who have the appropriate skills	☐	☐	☐	☐	☐
I feel I have had sufficient training to process referrals to PR	☐	☐	☐	☐	☐
I feel that sufficient resources are available in my organisation to support the processing of PR referrals	☐	☐	☐	☐	☐
My organisation provides adequate support for the PR referral process to enable it to be effective	☐	☐	☐	☐	☐
I am aware of audits or feedback reports within my organisation about the effectiveness of the PR referral process	☐	☐	☐	☐	☐
We adapt PR referral processes in light of feedback we receive	☐	☐	☐	☐	☐

For peer review only - http://bmjopen.bmj.com/site/about/guidelines.xhtml

Page 10: Your experience of the PR referral process (part iii)

What do you think are the main reasons that patients take up a referral to PR?

What do you think are the main reasons that patients who have been referred do not attend an assessment appointment?

For peer review only - http://bmjopen.bmj.com/site/about/guidelines.xhtml

Page 11: Communication and processes in pulmonary rehabilitation referral

Do you receive adequate communication from referrers about patients who have been referred to your service?

- Yes
- No

Please add comments to clarify if necessary

Which communication methods are effective for you when communicating with referrers? Select all that apply.

- Email
- Letter
- Telephone
- Other

If other, please give details

What do you think is important for effective communication between primary care and PR providers?

Do you send written reports to referrers about patients who have been referred to your service?

- Yes
- No

Please add comments to clarify if necessary

What tools, systems or processes are available in your organisation to support patients in their decision-making about whether to attend a PR assessment?

- Information about PR for patients
- Shared decision-making tools
- Other

If other please give details

For peer review only - http://bmjopen.bmj.com/site/about/guidelines.xhtml

For peer review only - http://bmjopen.bmj.com/site/about/guidelines.xhtml

Page 12: Improving referral and uptake to pulmonary rehabilitation

What do you think would make it easier for clinicians to refer patients to PR?

What do you think could be done to encourage patients to attend an assessment appointment following referral?

For peer review only - http://bmjopen.bmj.com/site/about/guidelines.xhtml

Page 13: Developing the pulmonary rehabilitation toolkit

Our research study aims to design and build a collection of resources (a 'toolkit') for use in primary care in order to support successful referral to PR. What do you think are the most important things that such a toolkit should contain or do in order for it to be acceptable and useful?

For peer review only - http://bmjopen.bmj.com/site/about/guidelines.xhtml

Page 14: Getting in touch

We are keen to gather examples of existing resources that may be useful to share through the toolkit. Do you have any resources that support the processing of PR referrals which you would be willing to share with the research team?

- Yes
- No

Would you like to enter the prize draw to win £200 of online shopping vouchers?

- Yes
- No

Thank you for completing this survey. All responses are confidential and results will be reported anonymously. Would you like us to share the results with you?

- Yes
- No

If you answered 'yes' to any of the above questions please leave your contact details here or, if you prefer to keep your responses anonymous, please email the research team directly: frances.early@addenbrookes.nhs.uk

Any other comments?

Page 15: The survey is now complete. Thank you for your time.

Key for selection options

3 - I confirm my agreement to the above statements

Yes
No

For peer review only - http://bmjopen.bmj.com/site/about/guidelines.xhtml

Supplement 3
REsTORE study focus group / interview schedule for HCPs in primary care

Perception of PR

1. How much do you know about PR?
2. How important is PR as treatment for COPD?
   a) Do you think PR is useful?
3. Do you think there are any differences in how you view PR and how other doctors, nurses and physiotherapists view it?
4. Is PR different from other parts of the COPD pathway? If yes – how?
   a) For example -Do you perceive it as being integral to the pathway?
   b) Are some components of the pathway are more important / effective / useful / acceptable?

Referral in practice

5. To what extent is PR referral part of your role?
6. What criteria do you use when referring people to COPD?
   a) How do you judge if it is worthwhile referring someone?
7. Can you describe what you do to refer someone to PR?
   a) Would you say there are differences in referral practice?
   b) Does everyone do it the same way?
   c) What tools/processes etc. are in place in your practice to ensure that PR referrals are made efficiently?
8. How would you describe the communication between you and the PR providers?
   a) For example, what types do you use and what is most effective - written, electronic/ telephone/person
9. How confident are you that referrals will be efficiently acted upon by other people in the pathway?
   a) Are there any efficiency standards for this?
10. Why do you think patients do or do not take up an offer?
11. Why do you think some HCPs might refer more people to PR than others?
    a) For example, differences between professionals or differences between practices i.e. variation within or between practices

Improving referral

12. What would make you more likely to refer to PR?
13. What would make it easier for you to refer to PR?
14. What would make it easier for patients to take up a referral?

Organisational support

15. Who drives PR referral forwards in your practice?
    a) What would make it easier to sustain levels of referrals?

Toolkit

16. Do you think a PR tool kit would be useful?
17. How would you judge whether a PR toolkit was worthwhile?

For peer review only

For peer review only - http://bmjopen.bmj.com/site/about/guidelines.xhtml

Supplement 4
REsTORE study focus group schedule for patients who have accepted a referral to PR

Introductions and welcome

1. **Do you know what Chronic Obstructive Pulmonary Disease (COPD) is?**
   a) Clarify understanding if patient is not sure.
   b) Offer COPD information leaflet.

2. **What is it like to live with COPD?**
   a) Can you tell me what you know about COPD and treatments?
   b) What do you have to do to manage your COPD, e.g. appointments, treatments, self-care…?
   c) How do you manage these things? Who or what helps you?
   d) Thinking about the demands of managing your COPD, what effect has this had on you and your life?

3. **Can you describe how you were referred to PR?**
   a) Who referred you?
   b) What did they tell you about the referral process or about PR, e.g. what PR is, why it might be a good idea for you? (PPI feedback indicated that this is a key question)
   c) How much did you know about PR before you attended?

4. **Why did you choose to attend PR?**
   a) Thinking about other people who could benefit from PR, what do you think they might need in order for them to be able to attend?
   b) Thinking about other people could benefit from PR, what do you think would make them want to attend?

5. **What things made it difficult for you to attend PR?**
   a) Challenges could be in their own life or in the healthcare system
   b) What do you have to do to be able to attend, e.g. change to routines, being open to trying something new…?
   c) How do you manage these things?
   d) Who or what helps you to attend PR?
   e) What would make attending PR easier?

6. **How has PR helped you compared to other treatments you are receiving for COPD?**
   a) How has attending PR affected you or your life (positive or negative)?

For peer review only - http://bmjopen.bmj.com/site/about/guidelines.xhtml
For peer review only

[Type text]

Supplement 5
REsTORE study focus group schedule for patients who have declined a referral to PR

1. **Do you know what Chronic Obstructive Pulmonary Disease (COPD) is?**
   a) Clarify understanding if patient is not sure.
   b) Offer COPD information leaflet.

2. **What is it like to live with COPD?**
   a) Can you tell me what you know about COPD and treatments?
   b) What do you have to do to manage your COPD, e.g. appointments, treatments, self-care…?
   c) How do you manage these things? Who or what helps you?
   d) Thinking about the demands of managing your COPD, what effect has this had on you and your life?

3. **Do you know what pulmonary rehabilitation (PR) is?**
   a) Clarify understanding if patient is not sure.
   b) Offer PR information leaflet.

4. **Can you describe how you were referred to PR?**
   a) Who referred you?
   b) What did they tell you about the referral process or about PR, e.g. what PR is, why it might be a good idea for you? (PPI feedback indicated that this is a key question)
   c) How much were you told about what happens in the PR classes?
   d) How much were you told about what you would need to do if you attended PR?

5. **Can you tell me the reasons why chose not to attend PR?**
   a) How did you decide whether or not it would help you?

6. **Were there any difficulties that put you off attending or made it impossible for you to attend PR?**
   a) Challenges could be in their own life or in the healthcare system
   b) What would you have had to do in order to attend, e.g. changed a routine, tried something unfamiliar …?

7. **What would make attending PR easier / what would help?**
   a) What would make attending PR more attractive?
   b) What kind of support might you need to attend PR?
   c) How would PR need to be different for you to want to attend?

8. **What do you know about how helpful PR could be compared to other treatments for COPD?**
   a) How important do you think PR is as a treatment?

Or, if interviewee believes that PR will be of no help to them:

9. **What other treatments are you on for COPD?**
   a) What do you think makes these more helpful in managing your COPD compared to PR?

For peer review only - http://bmjopen.bmj.com/site/about/guidelines.xhtml

Supplement 6

REsTORE study focus group schedule for patients who have not been referred to PR

1. Do you know what Chronic Obstructive Pulmonary Disease (COPD) is?

a) Clarify understanding if patient is not sure.
b) Offer COPD information leaflet.

2. What is it like to live with COPD?

a) Can you tell me what you know about COPD and treatments?
b) What do you have to do to manage your COPD, e.g. appointments, treatments, self-care…?
c) How do you manage this, who or what helps you?
d) Thinking about the demands of managing your COPD, what effect has this had on you and your life?

3. Do you know what pulmonary rehabilitation (PR) is?

a) Clarify understanding if patient is not sure.
b) Offer PR information leaflet.

4. Have you had any conversations with your nurse or GP about PR?

a) Who was the conversation with?
b) What was said?
c) What did they tell you about the referral process or about PR, e.g. what PR is, why it might be a good idea for you? (PPI feedback indicated that this is a key question)
d) What do you know about what PR involves?
e) If patient does not know about PR, offer information about PR (British Lung Foundation leaflets)

5. Would you like to be referred to PR?

If YES or NOT SURE go to question 6. If NO go to question 8.

6. If yes or not sure to question 5: What do you know about how helpful PR could be compared to other treatments for COPD?

a) How important do you think PR is as a treatment for COPD? [Note that this question may depend on why there were not referred]
b) What might you want from a programme like PR (i.e. an exercise and education programme) that would help you to manage your COPD?

7. What sort of problems or obstacles, if any, would you need to overcome to be able to attend a PR course?

a) Challenges could be in their own life or in the healthcare system
b) What do you think you would have to do in order to be able to attend, e.g. change routines, try something unfamiliar…?

8. What would make attending PR easy / what would help?

a) What would make attending PR attractive?
b) What kind of support might you need to attend PR?

END OF QUESTIONS

9. If no to question 5: Can you tell me the reasons why you would not want to be referred?

a) Can you tell me about whether you think it would or would not help you?

For peer review only - http://bmjopen.bmj.com/site/about/guidelines.xhtml

10. Are there any difficulties that would put you off attending or make it impossible for you to attend PR?

a) Challenges could be in their own life or in the healthcare system
b) What do you think you would have to do in order to be able to attend, e.g. change a routine, try something unfamiliar…?
c) What would make attending PR easy / what would help?
d) What would make attending PR attractive?
e) What kind of support might you need to attend PR?
f) How would PR need to be different for you to want to be referred?

END OF QUESTIONS

For peer review only

For peer review only - http://bmjopen.bmj.com/site/about/guidelines.xhtml

Supplement 7

REsTORE study focus group schedule for PR providers

Perception of PR

1. What do you think is the value of PR as a treatment for COPD?
    a) Explore any differences in how its value is perceived
    b) Why do you think some patients take up an offer but others don't?

Referral in practice

2. Can you describe what you do to process a PR referral?
    a) What tools/processes etc. are in place in your organisation to ensure that PR referrals are processed efficiently?
    b) What would make it easier for you to efficiently process referrals?
    c) What could be done in your organisation to make efficient processing of PR referral easier to sustain?

3. How effective is communication between referrers and PR providers?

4. In your experience why do you think some HCPs refer more patients to PR than others?

Improving referral

5. What could be done to increase referral to PR?
6. What could be done to increase uptake to PR?

Toolkit (Introduce what a toolkit is)

7. How would you judge whether a PR referral toolkit in primary care was worthwhile?

For peer review only

For peer review only - http://bmjopen.bmj.com/site/about/guidelines.xhtml

Supplement 8
REsTORE study interview schedule for commissioners

Perception of PR

1. How much do you know about PR?
   a) How valuable do you think PR is as a treatment for COPD?

2. Is PR different from other parts of the COPD pathway? If yes, how?

Referral in practice and how you support PR providers in making referrals

3. How confident are you as a commissioner that the PR referral pathway is performing efficiently?
   a) What tools or processes are in place in your organisation to support practices in making sure that PR referrals are made efficiently?

4. To what extent is it part of your role to support PR referral and utilisation in primary care?
   a) How do commissioners support practitioners in their assessment of whether or not to refer people to PR?

5. Why do you think some HCPs might refer more people to PR than others?
6. Why do you think patients do or don't take up an offer of PR?

Improving referral

7. What could commissioners do to make it easier for practices to successfully refer to PR?
8. What would make it easier for patients to take up a referral?

Support within your organisation

9. Who or what drives the requirement for PR utilisation in primary care forwards in your organisation?
10. In your organisation, what would make support for PR utilisation easier to sustain?

Toolkit

11. Do you think a PR referral toolkit would be useful?
12. How would you judge whether a PR referral toolkit was worthwhile?

For peer review only

For peer review only - http://bmjopen.bmj.com/site/about/guidelines.xhtml

UNIVERSITY OF CAMBRIDGE — Cambridge Institute of Public Health
Cambridge University Hospitals NHS Foundation Trust

DRAFT Patient Questionnaire

Thank you for taking part in our research about patients' with breathing problems and their experiences of care in General Practices in Cambridgeshire. In particular we are interested in how you found your consultation e.g. how long it took, if you understood things and if it was helpful. You can also give your comments about things you like, dislike or would like to see improved. Your answers remain anonymous and will not be directly reported to the staff at the practice.

Please answer all the questions and return the questionnaire to the University of Cambridge using the stamped addressed envelope.

Thank you for taking part in the survey

Dr Frances Early, Research & Evaluation Lead, Centre for Self-Management Support ,

contact: Tel: 01223 245151, Ext: 4858 e-mail: frances.early@medschl.cam.ac.uk

Section 1 Some questions about you

1.1 Are you Female / Male / Prefer not to answer (please delete as applicable)

1.2 Are you aged (please tick)

under 40 years ....

40 – 50 years ....

50 – 60 years ....

60 to 70 years ....

70 to 80 years ....

Over 80 years ....

or Prefer not to answer........... (tick if applicable)

1.3 About how many years have you had problems with your breathing? .... years

1.4 In the past have you ever attended Pulmonary Rehabilitation classes? (please delete as applicable)

Yes / No / Don't Know

Supp 9 Toolkit testing patient questionnaire

UNIVERSITY OF CAMBRIDGE Cambridge Institute of Public Health

Cambridge University Hospitals NHS
NHS Foundation Trust

Section 2. Some questions about your experience of the consultation

2.1 Overall, how easy or difficult did you find your consultation? (please tick)

Very easy

Quite easy

Neither easy or difficult

Quite difficult

Very difficult

2.2 Did you find the length of the consultation….(please tick)

Too long……………… About right………… Too short………..

2.3 During the consultation did any of the following happen? (tick those that apply to you)

Had some breathing tests ……….

Had a discussion with the nurse ……….

Watched a short video film ……….

Received a leaflet or written information ………

Received forms to fill out ………

Other (please specify below) ……….

……………………………………………………….

2.4 How helpful did you find the consultation? (please tick)

Very Helpful

Quite helpful

Neither helpful or unhelpful

Quite unhelpful

Very unhelpful

2.5 How easy or difficult was it to understand the consultation? (please tick)

Very easy

Quite easy

Neither easy or difficult

Quite difficult

Very difficult

Supp 9 Toolkit testing patient questionnaire

UNIVERSITY OF CAMBRIDGE Cambridge Institute of Public Health

Cambridge University Hospitals NHS
NHS Foundation Trust

2.6 Do you have any comments about the consultation or our survey? e.g. anything you liked, anything you disliked, any suggestions of what we could do better?

……………………………………………………………………………….

……………………………………………………………………………….

Thank You Again for Taking Part in the Survey

Frances Early, PhD, CPsychol , Research & Evaluation Lead, Centre for Self-Management Support ,

contact: Tel: 01223 245151, Ext: 4858 e-mail: frances.early@medschl.cam.ac.uk

For peer review only - http://bmjopen.bmj.com/site/about/guidelines.xhtml
